# A hypoarousal model of neurological post-COVID syndrome: the relation between mental fatigue, the level of central nervous activation and cognitive processing speed

**DOI:** 10.1007/s00415-023-11819-7

**Published:** 2023-06-25

**Authors:** Eva Maria Martin, Sven Rupprecht, Simon Schrenk, Fabian Kattlun, Isabelle Utech, Monique Radscheidt, Stefan Brodoehl, Matthias Schwab, Philipp A. Reuken, Andreas Stallmach, Thomas Habekost, Kathrin Finke

**Affiliations:** 1 Department of Neurology, Jena University Hospital, Jena, Germany; 2 Interdisciplinary Centre for Sleep and Ventilatory Medicine, Jena University Hospital Jena, Jena, Germany; 3 Department of Internal Medicine IV (Gastroenterology, Hepatology and Infectious Diseases), Jena University Hospital, Jena, Germany; 4 Center of Visual Cognition, University of Copenhagen, Copenhagen, Denmark; 5 Center for Sepsis Control and Care, Jena University Hospital, Jena, Germany; 6 Department of Psychology, Ludwig-Maximilians-University Munich, Munich, Germany

**Keywords:** Post-COVID, Arousal, Tonic alertness, Pupillary unrest, Fatigue, Cognitive dysfunction

## Abstract

**Background:**

Knowledge on the nature of post-COVID neurological sequelae often manifesting as cognitive dysfunction and fatigue is still unsatisfactory.

**Objectives:**

We assumed that cognitive dysfunction and fatigue in post-COVID syndrome are critically linked via hypoarousal of the brain. Thus, we assessed whether tonic alertness as a neurocognitive index of arousal is reduced in these patients and how this relates to the level of central nervous activation and subjective mental fatigue as further indices of arousal.

**Methods:**

40 post-COVID patients with subjective cognitive dysfunction and 40 matched healthy controls underwent a whole-report paradigm of briefly presented letter arrays. Based on report performance and computational modelling according to the theory of visual attention, the parameter visual processing speed (VPS) was quantified as a proxy of tonic alertness. Pupillary unrest was assessed as a measure of central nervous activation. The Fatigue Assessment Scale was applied to assess subjective mental fatigue using the corresponding subscale***.***

**Results:**

VPS was reduced in post-COVID patients compared to controls (*p* = 0.005). In these patients, pupillary unrest (*p* = 0.029) and mental fatigue (*p* = 0.001) predicted VPS, explaining 34% of the variance and yielding a large effect with *f*^2^ = 0.51.

**Conclusion:**

In post-COVID patients with subjective cognitive dysfunction, hypoarousal of the brain is reflected in decreased processing speed which is explained by a reduced level of central nervous activation and a higher level of mental fatigue. In turn, reduced processing speed objectifies mental fatigue as a core subjective clinical complaint in post-COVID patients.

**Supplementary Information:**

The online version contains supplementary material available at 10.1007/s00415-023-11819-7.

## Introduction

Following SARS-CoV2 infection, a high proportion of patients suffer from persisting symptoms, even after an initially mild to moderate-severe course [3, 29]. If these symptoms persist for more than three months unexplained by another condition, they are defined as “post-COVID syndrome” [68]. Around 30% of post-COVD patients show neurological and neuropsychiatric sequelae [74]. Amongst the most prevalent symptoms are fatigue and cognitive dysfunction, often reported in conjunction [4, 17, 61, 71], and seriously impairing quality of life as well as the ability to return to work [29, 31, 41]. To date, there is only unsatisfactory knowledge on the nature of the cognitive deficit in post-COVID patients and its relationship with fatigue and, thus, on promising specific treatment targets.

In the present study, we assumed that a critical link between cognitive dysfunction, often described as “brain fog”, and fatigue in post-COVID syndrome is a reduction in the brain’s general level of arousal. Arousal exists on a behavioral continuum between sleep and alert hypervigilance and fear [65, 75]. Normal waking alertness as a state between the extremes is most optimal for attentional cognitive performance [32, 80]. In various disorders accompanied by fatigue, such as multiple sclerosis [49], Parkinson’s disease [38], post-polio [9] and cancer [50] behavioral, EEG and pupillary indicators of hypoarousal have been found. On a neurocognitive level, the brain’s general arousal level is linked to the degree of tonic attentional alertness, i.e. its endogenous, intrinsic cognitive readiness state [58]. Tonic alertness undergoes rather slow, e.g., circadian changes [30, 72], different from fast changes in “phasic alertness” initiated by external cues or stimuli. Especially in longer or tedious tasks, reduced tonic alertness thus results in difficulties in staying prepared to process and respond [53, 58, 72]. Accordingly, patients with persisting fatigue due to multiple sclerosis [48] and Parkinson’s disease [7]—and also patients with post-COVID syndrome [39, 66]—show slowed reaction times in simple-response tasks. To evaluate the relevance of hypoarousal as an underlying mechanism, we measured indices of potential hypoarousal on three different levels in post-COVID patients.

As a neurocognitive measure of arousal, we quantified tonic alertness using an assessment based on Bundesen’s computational “theory of visual attention” (TVA) [11–13]. TVA-based measurement delivers the parameter visual processing speed (VPS) *C* as a quantifiable proxy of tonic alertness. VPS is estimated quantitatively by modelling the accuracy of verbal report of letter arrays briefly presented on a computer screen. Importantly, TVA-based assessment allows the exact quantification of VPS as a proxy of tonic alertness, mathematically independent from (and controlled for) other attentional parameters, i.e. visual short-term memory (vSTM) capacity, perceptual threshold and top-down control, within the same paradigm. Furthermore, it is independent of motor side effects that might occur in post-COVID patients with their frequently persisting (neuro-)muscular alterations [63, 73].

As a neurophysiological measure of central nervous activation, i.e. the brain’s level of arousal, spontaneous oscillations in pupil diameter in darkness were assessed using the pupillographic sleepiness test (PST) [43, 60, 78]. In an alert state, dark-adapted pupils are large and stable, while, as arousal levels decrease, pupil diameter decreases and there is an increase in slow pupillary oscillations characterized by augmenting amplitude [22, 42, 44]. Such pupillary “fatigue waves” may originate from fluctuations in the activity of noradrenergic neurons in brainstem nuclei like the locus coeruleus (LC) [57] known to also be involved in the neuromodulation of arousal and tonic alertness [45]. The pupillary unrest index (PUI) is determined by absolute values of cumulative dark-adapted pupil size changes on the basis of mean values of consecutive data sequences [43, 77]. Higher PUI values along with lower average pupil diameters reflect lower levels of central nervous activation.

As a measure of the subjective experience of arousal, mental fatigue [61] ratings based on the respective subscale of the Fatigue Assessment Scale (FAS) [46] were used. In multiple sclerosis patients, there is evidence that the subjectively perceived level of fatigue predicts the extent of tonic alertness reduction [76].

We assumed that hypoarousal is a central mechanism underlying symptoms in post-COVID patients complaining of cognitive dysfunction and fatigue. Based on this, we hypothesized that the rate of visual information uptake, i.e. VPS, as a measure of tonic alertness, is reduced in these patients. To test this, we compared post-COVID patients’ VPS to age-, sex- and education-matched healthy control participants. In a second step we explored whether and how a potential VPS reduction indicating reduced arousal at the neurocognitive level is related to further indices  of hypoarousal in post-COVID patients on the neurophysiological and the subjective experience level. We hypothesized that VPS is explained by the level of central nervous activation measured through PUI and by the level of mental fatigue measured through self-rating.

## Method

### General procedure

A priori power analysis was conducted using G*power 3.1.9.6 [27] to estimate the minimum sample size. Expecting a large effect size on VPS in post-COVID patients based on Crivelli et al. [21] with an *α*-level of 0.05 and power of 90%, the minimum total sample size comparing two groups was *n* = 28, and for multiple regression analysis including 6 predictors *n* = 36. A sufficiently large sample of 40 patients fulfilling the NICE criteria for post-COVID syndrome [68] with subjective cognitive dysfunction after polymerase chain reaction confirmed SARS-CoV2 infection seeking treatment at the NeuroCOVID-Centre of the Department of Neurology of Jena University Hospital (JUH) and 40 healthy control participants without prior SARS-CoV2 infection matched for age, gender and education were recruited. Clinical examination and neuropsychological assessment of the patient group were performed at JUH NeuroCOVID-Centre. Pupillography was conducted in the JUH Interdisciplinary Centre for Sleep and Ventilatory Medicine. Due to strict hospital access restrictions during the pandemic, clinical examination and neuropsychological assessment of the healthy control group was performed in an external JUH science laboratory where pupillography could not be assessed. Exclusion criteria for all participants comprised any history of neurological (e.g. epilepsy, multiple sclerosis, stroke), psychiatric (e.g. depression) diseases and non-corrected visual impairment. Intact vision was assessed using the MARS letter contrast sensitivity test [1]. Our study followed the Helsinki II ethics regulations and was approved by the ethics committee of JUH (No. 5082-02/17) and written informed consent was given before inclusion in the study.

### Assessment

The following self-rating questionnaires were completed by participants of both groups:

*Mental fatigue* was assessed by the mental fatigue subscale of the Fatigue Assessment Scale (FAS) [46] which consists of five items on a five-point-scale (1 = never, 5 = always) with higher scores corresponding to higher levels of mental fatigue.

*Depression* was assessed by the depression subscale of the German version of the Hospital Anxiety and Depression Scale (HADS-D) [36, 69] which consists of seven items. Scores range between 0 and 24 with higher scores indicating higher levels of depressive symptoms.

*Sleepiness* was assessed by the German version of the Epiworth Sleepiness Scale (ESS) [6] consisting of eight items asking for the probability of falling asleep from 1 (unlikely) to 3 (very likely) in eight different situations. Scores range between 0 and 24 with higher scores indicating higher sleepiness and a score of ≥ 10 indicating excessive daytime sleepiness.

*Pupillary unrest index* (PUI) was assessed in the post-COVID patient group with the pupillographic sleepiness test (PST) using the AMTech F2D2 pupillograph (AMTech Pupilknowlogy, Dossenheim, Germany). Time of pupillographic assessment was held constant and always took place between 11.00 am and 13.00 p.m. Patients were asked to put on blotted infrared video camera AMTech-goggles in a completely dark room. 90 s were given to adjust to the dark before patients were asked to fixate on two built-in red light diodes (left eye and right eye, wavelength 880 nm). The pupillary behavior was recorded for a period of 11 min using an infrared-light-sensitive video camera at a 25 Hz (40 ms) sampling rate and a resolution of 0.05 mm. Recording took place in complete silence. The goggles were connected to a laptop and the WinPST Version 6.20.2.1 software was used to automatically calculate the PUI. Artifacts resulting from factors like eye movements (greater than 15 degrees of visual angle), head movements, or eye blinks lasting longer than 3 s were identified, removed automatically and linear interpolation was used to substitute these missing data points. The PUI was derived from cumulative changes in pupil diameter. The diameter (in mm) was calculated involving a repetitive circle fitting process that relies on detecting the initial Purkinje reflex on the cornea and identifying the pupil’s edge points [43]. The data was preprocessed by determining the average value for sets of 16 consecutive values before being cumulated. This served as a simple low-pass filter, eliminating high-frequency noise from subsequent calculations. The absolute differences between each 16-value average and the next one were then summarised for each 82.5 s data segment (resulting in 127 differences for one segment). This sum was normalized over a 1-min period and referred to as the PUI (in mm/min) for the corresponding data segment. The final, total PUI value (mm/min) was then averaged over all eight 82.5 s data segments. In essence, the PUI represents the sum of absolute changes in pupil diameter (measured in mm) based on a sampling frequency of 1.5625 Hz (equivalent to 25 Hz divided by 16); see [43] for further detail. A completely stable pupil diameter would result in a value of 0 mm/min, while higher instability of the pupil diameter produces higher values in PUI. In addition, the average pupil diameter in mm (PD) over 11 min was computed by the software.

*VPS and three additional visual attention parameters* were assessed by applying the whole and partial report paradigms based on the mathematical TVA framework [11–13]. TVA is related to the biased-competition view of visual attention [25]. Accordingly, objects in the visual field are processed in parallel and race for selection into a visual short-term (vSTM) store with limited capacity. Only those objects that are processed fastest will win the race and be selected. Processing speed, i.e. the rate of processing an individual object, is decisive for the probability of the object getting selected and depends on the object’s attentional weight. The magnitude of attentional weight allocated to an object is influenced by both bottom-up (such as saliency of an object) and top-down (such as the congruence of the object's features with selection-relevant target features) factors. Once selected, the object is consciously represented and can be verbally reported. If the capacity of the store is reached, the process of selection is terminated. TVA-based whole and partial report paradigms are optimized for generating precise parameters characterizing the individual visual attentional capacity and selectivity.

*General whole and partial report procedure*: Letters were briefly presented on a computer screen. Each trial started with a 1000 ms presentation of a central fixation point. After a 250 ms delay, randomly chosen letters from the alphabet (with the exception of I, Q and Y) appeared in red and/or blue, with each letter being presented only once in a given trial. Exposure durations were determined individually during a pretest to reach an overall performance level optimized for the parameter estimation process. Letters were either followed by a mask for 500 ms or unmasked. Masking served to prevent the effects of visual persistence [70] (see supplement’s section 1.1 for technical details).

**Fig. 1:**
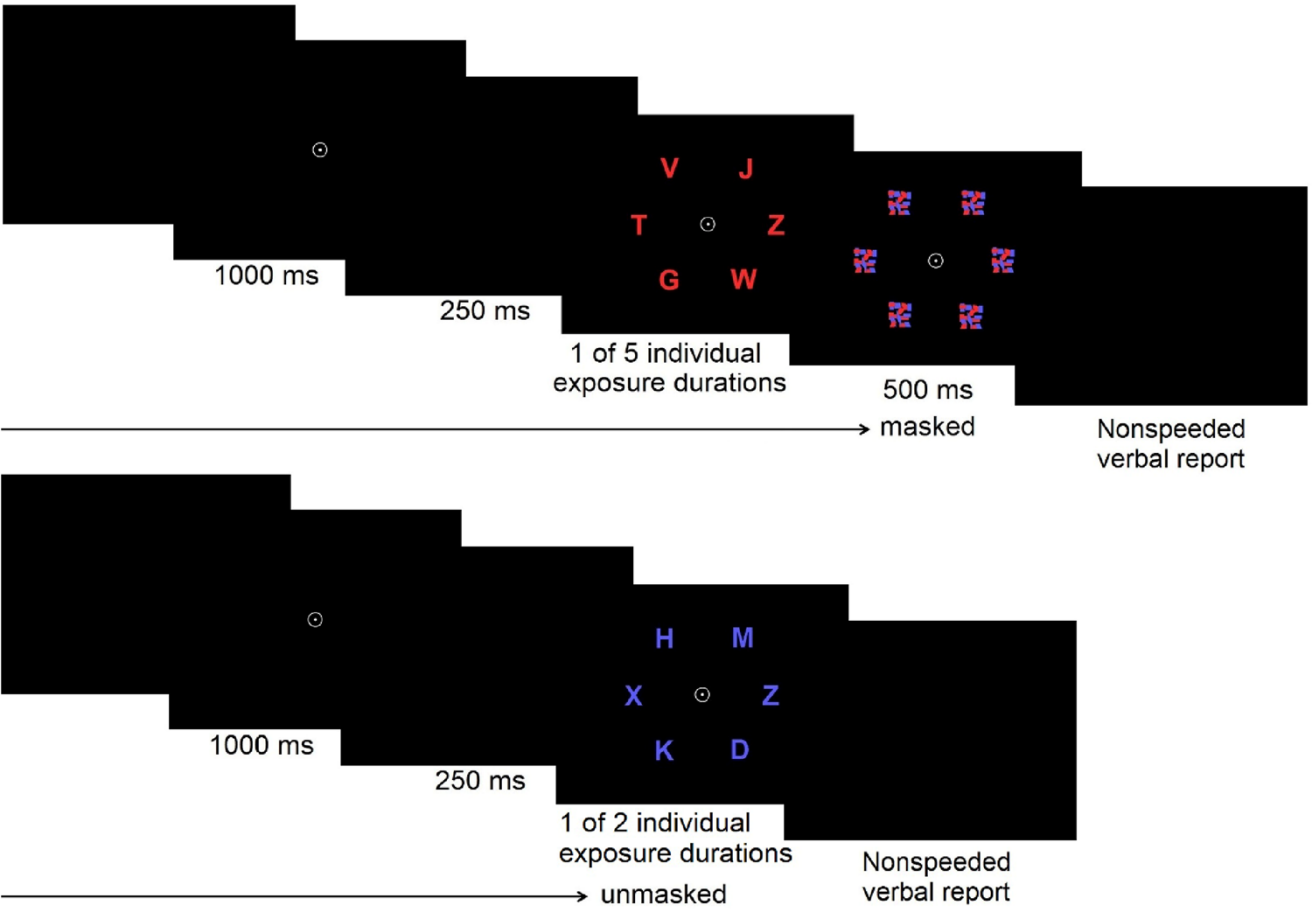
TVA whole report paradigm: The task consisted of verbally reporting as many letters recognized as possible after each trial. In a trial, a central fixation point is presented for 1000 ms. After a brief delay of 250 ms, six letters in red or blue are presented in an imaginary circle for 1 of 5 individually adjusted exposure durations (that were calibrated during the pretest) appeared either masked or unmasked (for the second shortest or the longest individual exposure duration), therefore resulting in 7 effective exposure durations. This was followed by an unspeeded report of all letters seen

*Whole report procedure*: In the whole report paradigm, six letters, either all red or all blue were circled equidistantly 5.73° of visual angle around the fixation point. The task consisted of verbally reporting as many letters as possible. In the pretest phase, five different exposure durations were determined per individual to collect performance at near-threshold and near-maximum levels. See supplement’s Sect. 1.1.1 for technical procedure details. In five conditions, letters were presented for these individually pre-defined exposure durations and were masked (see Fig. 1). Additionally, in two unmasked conditions, letters were presented for the second shortest and the longest exposure duration. Consequently, seven “effective” exposure duration conditions (five masked and two unmasked) were defined. For each of these seven conditions, 20 trials were included resulting in a total of 140 trials presented in four test blocks.

**Fig. 2:**
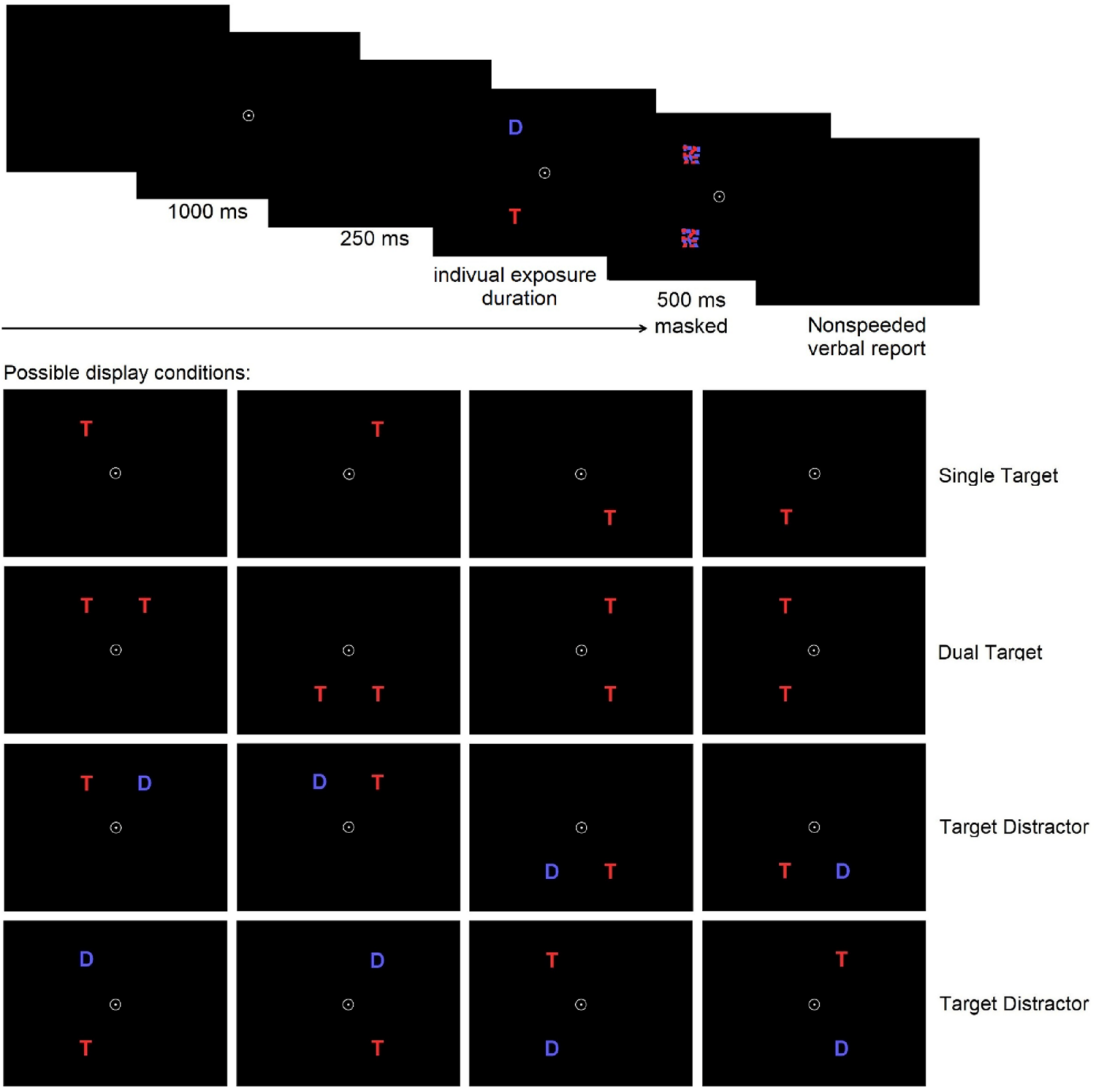
Partial report paradigm: The task consisted of only verbally reporting red letters and ignoring blue letters. In a trial, the central fixation point is presented for 1000 ms. After a brief delay of 250 ms, the letters (T = target = red letters; D = distractor = blue letters) are presented for an individually adjusted exposure duration (calibrated during the pretest) in one of sixteen possible display conditions always masked (500 ms) and followed by an unspeeded report of red letters seen. Targets were presented in the corners of an imaginary square (7.5 cm × 7.5 cm) around the fixation point

*Partial report procedure:* In the partial report paradigm, on each trial, either one or two letters (1 target, 2 targets or a target plus distractor) were briefly presented in the corners of an imaginary square (see Fig. 2). If two letters were presented, they would always appear vertically or horizontally, never diagonally (see Fig. 2). The task consisted of verbally reporting only red letters (= targets) and ignore blue letters (= distractors). Letterd (see Fig. 2) appeared in randomized order and were always masked. The partial-report task consisted of 16 conditions (4 single-target T, 8 target plus distractor T-D, 4 dual-target conditions T-T), counterbalanced across all six blocks (see Fig. 2). In the pretest, 24 calibration trials were used to determine one individually adjusted exposure. During the test phase, 288 trials were then executed with the pre-defined exposure duration split into six test blocks with 48 trials including three trials of the 16 conditions each (see supplement’s Sect. 1.1.2 for further details of calibration and exposure duration adjustment).

*Parameter estimation of VPS and other TVA parameters:* Parameter estimation was conducted according to a maximum likelihood approach based on the underlying estimation algorithms described by Kyllingsbaek [40]. The probability of identifying an object is based on the accuracy of correctly reported letters in relation to exposure duration. Whole report data allow for modelling an exponential growth function based on the following estimates: VPS (visual processing speed *C*) i.e. the total rate of reported letters per second, is represented by the initial slope of the curve. Visual perceptual threshold *t0* is represented by the intercept of the curve with the x-axis indicating the minimal effective exposure duration. vSTM capacity *K* is represented by the curve’s asymptote, indicating the maximum number of letters represented in an instant (see Fig. 4 for a representative healthy control participant and post-COVID patient, respectively). The TVA model explained, on average, 98% (mean *R*^2^) of the variability in the observed mean reported scores across all exposure duration conditions of the whole report for the healthy control group and 96% for the post-COVID patient group, indicating high goodness of fit. Goodness of fit did not differ between groups, *t*(72.79) = − 0.81, *p* = 0.420.

Partial report data allow for derivation of the attentional selectivity parameter estimate top-down control *α*. From the partial report accuracy across different conditions, attentional weights are derived for targets (*w*T) and distractors (*w*D). Parameter *α* is defined as the ratio of distractor to target weights (*w*D/*w*T) and reflects top-down efficacy, i.e., the ability to prioritize task-relevant over task-irrelevant information (see supplement’s Sect. 1.1.3 for further details on parameter α and partial report TVA model fit).

### Statistical analysis

Statistical analysis was run using R [20] version 4.2.1. Patient and healthy control groups were compared in terms of basic socio-demographic and basic clinical information using two-tailed Wilcoxon rank sum tests (*α* = 0.05) and compared in terms of self-reported fatigue and depression as well as VPS and the other TVA parameters (vSTM capacity *K*, visual perceptual threshold *t0* and top-down control *α*) using one-tailed Wilcoxon rank sum tests (= 0.05). For Wilcoxon’s rank sum test, the effect size was determined calculating *r* values. Effect size is medium when 0.3 < *r* < 0.5 and large when *r* > 0.5 [19]. Non-overlap was calculated using resampling via non-parametric bootstrapping (1000 replications) to estimate the non-overlapping area between two kernel density estimations from our data. Associations within the patient group between VPS and PUI, self-rated mental fatigue (mental subscore FAS), depressive symptoms (depression subscore HADS-D), sleepiness (ESS score), age, time from infection (in days) and the other TVA parameters (*K*, *t0*, *α*) were first explored graphically with scatterplots with regression line fit. Then, the conservative Spearman’s rank correlation method to control for outlier effects was applied. Mediation analysis within the patient group was conducted using PROCESS in R [33, 34]. To explore predictors for VPS variance, multiple linear regression analysis within the patient group was conducted. Hereby, PUI and mental fatigue were included as predictors of interest in the first model. Based on findings reported in previous studies, age [26], depressive symptoms [8] and time from infection [8] as well as sleepiness [15] were included as covariates in the second model. Then, the first model was compared with the second model by applying an ANOVA to test whether additionally including the covariates in the model leads to a significant increase in variance explanation. To estimate generalizability, confidence intervals around coefficients as well as confidence intervals of the models were bootstrapped (1000 replications). We controlled for a false discovery rate (FDR) of 5% among all tests by using the Benjamini–Hochberg method [79].

## Results

### Sample description

Basic socio-demographic information for both groups and basic clinical post-COVID patient data are presented in Table 1. In the post-COVID patient group, on average, 1.17 years had passed since acute infection and around a third had been treated at the hospital during acute infection (none of them at the ICU, none of them had received invasive ventilation). There were no significant differences between groups in terms of age (*W* = 632, *z* = − 1.61,* r* = 0.18, *p* = 0.135), education (*W* = 925.5, *z* = − 1.42, *r* = 0.15,* p* = 0.171) or gender ratios, *χ*^2^(1, 80) = 2.257, *p* = 0.125).

**Table 1:** Basic sociodemographic and clinical data by group

	Healthy controls (*n* = 40)	Post-COVID patients (*n* = 40)
Age (years)		
* M (SD*)	44.05 (12.25)	47.95 (8.44)
Range	24.00–68.00	24.00–59.00
Sex		
Female	26 (65%)	32 (80%)
Male	14 (35%)	8 (20%)
Education (school years)		
* M* (*SD*)	11.25 (1.34)	10.75 (1.53)
Range	9.00–13.00	9.00–13.00
Time from infection (days)		
* M (SD*)	–	428 (94.85)
Range		154–744
Hospitalization during infection		
Yes	–	13 (32.5%)
No		27 (67.5%)

### Pupillary unrest, average pupil diameter and comparison of self-rated mental fatigue, depressive symptoms and sleepiness between healthy controls and post-COVID patients

Average PUI and PD values within the Post-COVID group are listed in Table 2. Higher PUI values were correlated with smaller average PD values (*p* = 0.044; see Table 4). Post-COVID patients showed higher fatigue ratings in the mental subscale of the FAS, higher depression ratings in the depression subscale of the HADS-D and higher daytime sleepiness ratings in the ESS compared to healthy controls with large effect sizes with *r* > 0.5 (all *p* < 0.007) (Table 2).

**Table 2:** Pupillary unrest, average pupil diameter and comparison of self-rated mental fatigue, depressive symptoms and sleepiness between healthy controls and post-COVID patients

	Healthy controls (*n* = 40)		Post-COVID patients (*n* = 40)		Wilcoxon rank sum test			
	*M *(SD)	Mdn (IQR)	*M* (SD)	Mdn (IQR)	*W*	*Z*	*p*	*r*
PUI	–	–	4.39 (1.76)	4.15 (2.22)	–	–	–	–
PD	–	–	6.88 (0.97)	7.09 (1.22)	–	–	–	–
Mental fatigue	8.97 (2.90)	8.00 (2.00)	18.65 (4.17)	17.00 (8.25)	91	− 6.90	< 0.001	0.77
Depression	2.72 (4.55)	1.50 (4.00)	6.85 (3.98)	5.50 (6.00)	249	− 4.72	0.006	0.53
Sleepiness	7.30 (4.20)	7.00 (5.00)	11.24 (4.31)	11.00 (5.50)	329.5	− 4.72	< 0.001	0.53

PUI = pupillary unrest index (in mm/s); PD = average pupil diameter (in mm); Mental fatigue = Fatigue Assessment Scale (FAS) mental fatigue subscore; Depression = Hospital Anxiety and Depression Scale (HADS-D) depression subscore; Sleepiness = Epworth Sleepiness Scale (ESS) score. FDR-corrected *p*-values

### Comparison of VPS and other TVA parameters between healthy controls and post-COVID patients

**Fig. 3:**
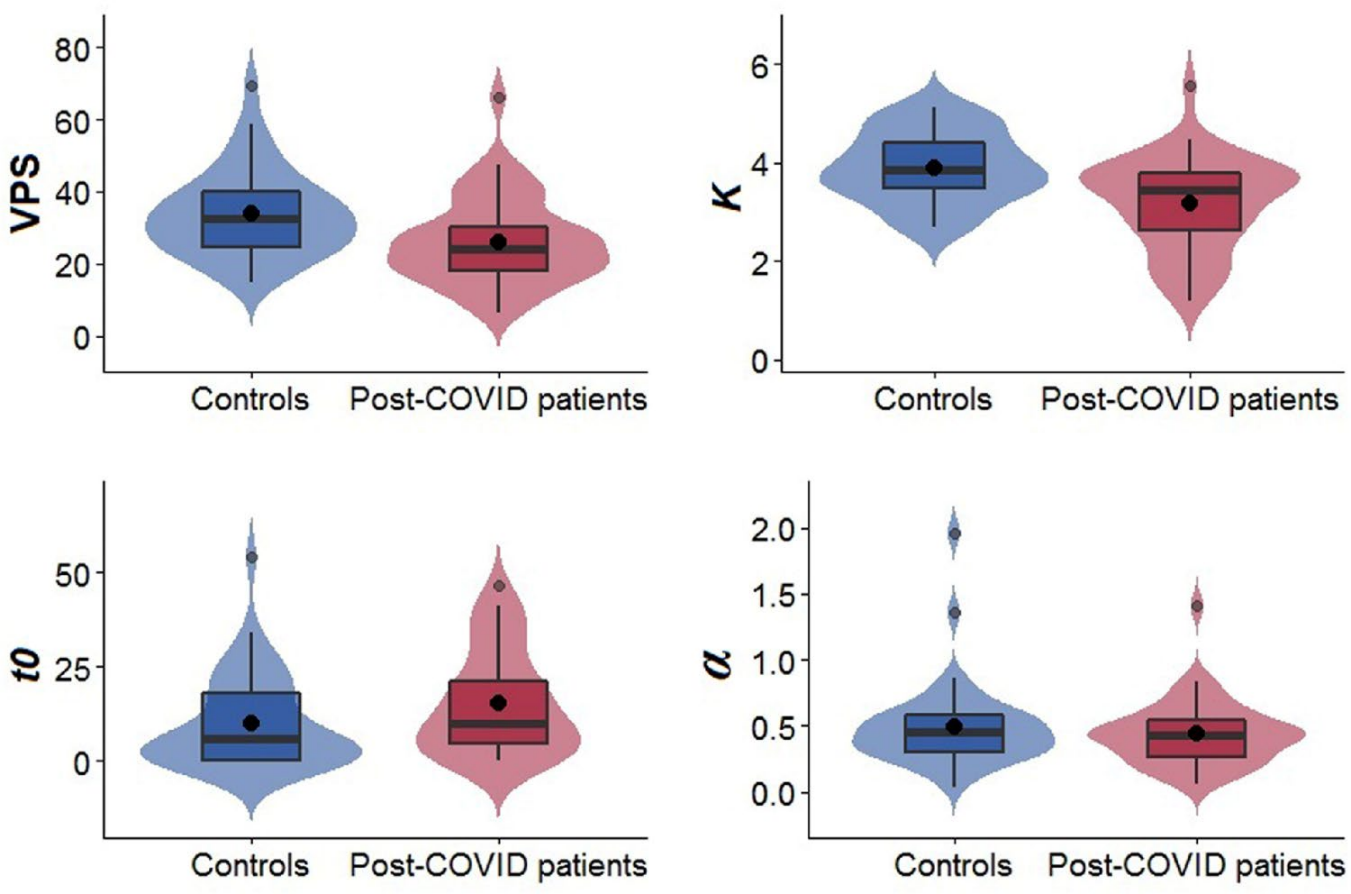
Distributions, medians (line) and means (point) in *n* = 40 post-COVID patients and *n* = 40 sociodemographically matched healthy controls for VPS = visual processing speed *C* (in letters per second); *K* = visual short-term memory capacity (maximum number of letters); *t0* = visual perceptual threshold (in ms); and *α* = top-down control (distractor/targets)

Table 3 depicts means, standard deviations and results of group comparison for VPS and the other TVA parameters in healthy control participants and post-COVID patients. VPS was lower in patients than in control participants with a medium effect size (*r* = 0.34, *p* = 0.005) and an estimated 25% non-overlap of the two distributions of VPS scores. Among the other TVA parameters, vSTM capacity *K* was lower compared to healthy controls with a medium effect (*r* = 0.39, *p* = 0.001) and an estimated 26% non-overlap of the two distributions of *K* scores. Visual perceptual threshold *t0* was higher in patients compared to controls with a small effect size (*r* = 0.25, *p* = 0.037) and an estimated 16% non-overlap of the two distributions of *t0* scores. Top-down control *α* did not differ between groups. In Fig. 3, distribution means and medians for VPS and the other TVA parameters are depicted per group. In the supplement’s Sect. 2.2, estimated overlap of distribution curves is depicted (Fig. 6).

**Table 3:** Group differences between post-COVID patients and healthy controls for VPS (processing speed *C*) and other visual attention parameters

	Healthy controls (*n* = 40)		Post-COVID patients (*n* = 40)		Wilcoxon rank sum test			
	*M *(SD)	Mdn (IQR)	*M* (SD)	Mdn (IQR)	*W*	*z*	*p*	*r*
VPS	33.72 (11.92)	33.77 (15.35)	26.23 (12.26)	24.21 (14.92)	1092.00	− 3.03	0.005	0.34
*K*	3.89 (0.71)	3.85 (0.96)	3.16 (0.95)	3.43 (1.19)	1146.00	− 3.52	0.001	0.39
*t0*	9.92 (12.19)	5.96 (18.73)	15.16 (13.65)	10.00 (19.33)	594.50	− 2.27	0.037	0.25
*α*	0.50 (0.33)	0.45 (0.27)	0.44 (0.25)	0.43 (0.28)	875.00	− 0.30	0.765	0.03

VPS = visual processing speed *C* (in letters per second); *K* = visual short-term memory capacity (in a maximum number of letters); *t0* = visual perceptual threshold (in ms);* α* = top-down control (distractor/targets). FDR-corrected *p-*values

#### Whole report performance of a representative healthy control participant and post-COVID patient

**Fig. 4:**
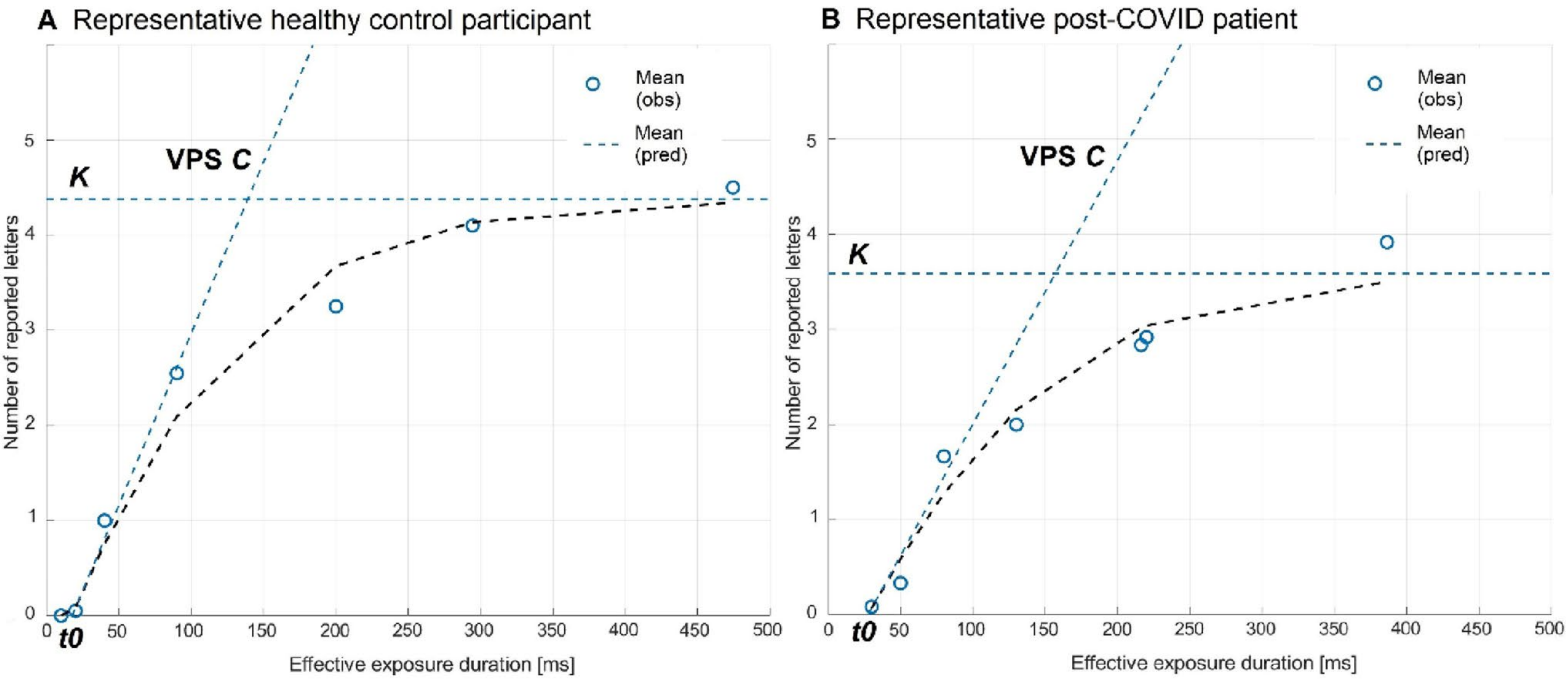
Representative whole report performance of a healthy control participant (**A**) and a post-COVID patient (**B**) as a function of exposure duration. Circles indicate observed values (= obs), dashed lines indicate predicted values based on the best model fit (= pred). The slope represents VPS (visual processing speed *C* in letters per second), the dashed horizontal line represents *K* (maximum visual short-term memory capacity in a maximum number of letters) and the intercept of the curve with the x-axis represents *t0* (visual perceptual threshold in ms)

In Fig. 4, the whole report performance of a representative healthy control participant and a post-COVID patient is depicted. The mean number of correctly reported letters as a function of effective exposure duration are presented. Circles indicate observed values and dashed curves represent the maximum likelihood fits to the observed data, which are closely corresponding.

Both curves start at the visual perceptual threshold *t0*, which represents the minimum necessary exposure duration for conscious perception. Following this, both curves initially increase systematically indicating an increasing number of correctly reported letters with increasing exposure duration. However, the slope of the function in *t0*, which represents VPS, is considerably steeper in the representative healthy control participant’s curve (Fig. 4A) than in the post-COVID patient’s curve (Fig. 4B). Visual perceptual threshold *t0*, represented by the origin of the function, is higher in the representative post-COVID patient compared to the representative healthy control. As effective exposure duration increases, report performance in both individuals approaches an asymptote, which represents vSTM capacity *K*: Here, the representative post-COVID patient’s asymptote is lower than the one of the healthy control, illustrating that the number of letters that can be represented in a given instance is reduced in the post-COVID patient. See supplement’s Sect. 2.4 for all individual whole report performance curves.

### Correlative analyses

**Fig. 5:**
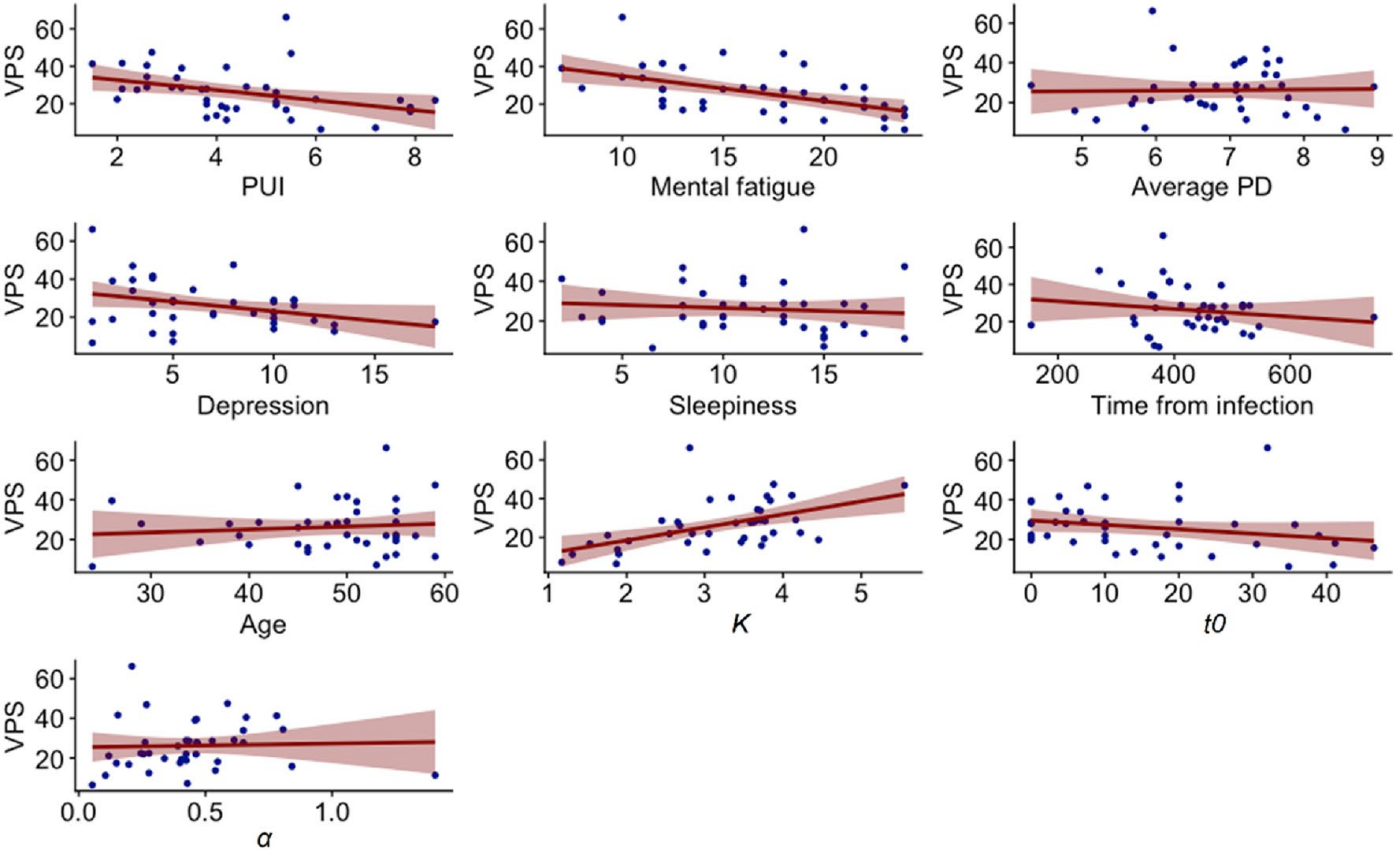
VPS = visual processing speed *C* (letters/second) and PUI = pupillary unrest index (mm/min): Mental fatigue = subscore of the Fatigue Assessment Scale (German version); Average PD = average pupil diameter (mm); Depression = depression subscore of the Hospital Anxiety and Depression Scale (German version); Sleepiness = Epworth Sleepiness Scale (German version) score; Time from (SARS-CoV2) infection (days); Age in years; *K* = visual short term memory capacity (maximum number of letters); *t0* = visual perceptual threshold (ms);* α* = top-down control (distractor/targets) within the post-COVID patient group (*n* = 40)

We first inspected graphically the relationships between VPS and PUI, mental fatigue, the relevant clinical measures assessed and the other TVA-based attention parameters in the post-COVID patient group (Fig. 5). Then, we ran statistical correlation analyses between all measures (Table 4).

**Table 4:** Spearman’s rank correlations between VPS, PUI, mental fatigue, average PD, depressive symptoms, sleepiness, time from infection, age, vSTM capacity *K*, visual perceptual threshold *t0* and top-down control* α* within the post-COVID patient group

	VPS	PUI	Mental fatigue	PD	Depression	Sleepiness	Time	Age	*K*	*t0*
PUI	− 0.508^*^									
Mental fatigue	− 0.535^**^	0.234								
PD	0.094	− 0.447^*^	0.050							
Depression	**− **0.266	0.097	0.498^*^	**− **0.131						
Sleepiness	**− **0.194	0.116	0.318	**− **0.163	0.345					
Time	**− **0.115	0.005	0.133	0.063	0.378	**− **0.016				
Age	0.042	**− **0.053	0.014	**− **0.364	0.095	0.169	**− **0.315			
*K*	0.574^**^	− 0.433^*^	**− **0.206	0.124	**− **0.037	**− **0.186	**− **0.014	0.001		
*t0*	**− **0.369	0.379	0.258	**− **0.112	0.110	0.309	**− **0.419	0.093	**− **0.258	
*α*	0.250	**− **0.247	**− **0.249	**− **0.024	**− **0.022	0.124	**− **0.160	0.145	0.175	0.084

FDR-corrected *p*-values. Statistically significant correlations are in bold. *Indicates *p* < 0.05. **Indicates *p* < 0.01. VPS = visual processing speed *C* (letters/second); PUI = pupillary unrest index (mm/min): Mental fatigue = Fatigue Assessment Scale (FAS, German version) mental fatigue subscore; PD = average pupil diameter (in mm); Depression = Hospital Anxiety and Depression Scale (HADS-D, German version) depression subscore; Sleepiness = Epworth Sleepiness Scale (ESS, German version) score; Time = time from (SARS-Cov2-) infection (days); Age (years); *K* = visual short-term memory capacity (maximum number of letters); *t0* = visual perceptual threshold (ms);* α* = top-down control (distractor/targets). Spearman’s rank correlations within the post-COVID patient group (*n* = 40)

#### Correlations between VPS, PUI, mental fatigue, relevant clinical and sociodemographic measures

As can be seen in Fig. 5, VPS was correlated with higher PUI (*p* = 0.01) and higher mental fatigue (*p* = 0.004). No correlation was found between VPS and depressive symptoms, sleepiness, age, time from infection, or average PD. See supplement’s Sect. 2.1 for intercorrelations in the healthy control group.

#### Further correlations and mediation analysis

Lower VPS was related to lower vSTM capacity *K* (*r* = 0.574, *p* = 0.005). Similarly to VPS, also vSTM capacity *K* was related to higher PUI (*p* = 0.005). Given the close association between VPS and vSTM capacity *K*, we first assessed whether this was also true for the control group. The correlation coefficient for VPS and *K* in the control group was only moderate (*r* = 0.336,* p* < 0.001) which is representative for normal participants (e.g. Finke et al. [28]). Given this difference between groups, we then tested in a mediation analysis whether the association between vSTM capacity *K* and PUI is explained by VPS. Indeed, VPS fully mediated the association between PUI and *K* (see supplement’s Sect. 2.3 for the full model). That is, we found a non-significant bootstrapped (1000 samples) direct effect of PUI on *K, b* = − 0.130, *SE* = 0.094, 95% CI[LL, UL] = [− 0.303, 0.073], and a significant bootstrapped (1000 samples) indirect effect, *b* = − 0.089, *SE* = 0.066, 95% CI[LL, UL] = [− 0.250, − 0.011]. Incidentally, higher mental fatigue was found to be correlated with higher depression scores (*p* = 0.014).

### Regression analysis for predictors of VPS in post-COVID patients

For VPS, the first regression model was significant (*p* < 0.001). PUI (*p* = 0.029) as well as mental fatigue (*p* = 0.001) predicted VPS (Table 5). Effect size was large [19] with *f*^2^ = 0.51. The second model which additionally included the covariates depression, sleepiness, time from infection and age also reached significance (*p* = 0.006). Here, only PUI (*p* = 0.029) and mental fatigue (*p* = 0.008) predicted VPS, while none of the covariates did. An ANOVA comparing the first with the second model revealed that additionally including the covariates in the model did not lead to increased VPS variance explanation, *F*(4, 3675.9) = 0.42, *p* = 0.801. Bootstrap (1000 samples) confidence intervals indicate the generalizability of both models.

**Table 5:** Multiple regression results with VPS as the criterion and PUI and mental fatigue as predictors as well as depressive symptoms, sleepiness, time from infection and age as covariates

Multiple regression results: VPS as the criterion						
Predictor	*b*	*b* 95% CI [LL, UL]	*beta*	*beta* 95% CI [LL, UL]	*p*	Fit
Model 1						
(Intercept)	**55.31**	[44.45, 68.17]			< 0.001	
PUI	**− 2.10**	[**− **3.42, **− **0.51]	**− **0.30	[**− **0.57, **− **0.06]	0.029	
Mental fatigue	**− 1.20**	[**− **1.97, **− **0.58]	**− **0.48	[**− **0.67, **− **0.27]	0.001	
						*R*^*2*^ = 0.373 *R*^*2*^adj. = 0.338 95% CI [0.22,0.64] FDR-adj. *p* < 0.001
Model 2						
(Intercept)	**57.51**	[26.66, 86.19]			0.001	
PUI	**− 2.24**	[**− **3.71, **− **0.07]	**− **0.32	[**− **0.63, **− **0.01]	0.029	
Mental fatigue	**− 1.16**	[**− **1.99, **− **0.24]	**− **0.46	[**− **0.75, **− **0.10]	0.008	
Depression	**− **0.08	[**− **1.40, 0.99]	**− **0.03	[**− **0.40, 0.36]	0.873	
Sleepiness	0.14	[**− **0.69, 1.04]	0.05	[**− **0.29, 0.33]	0.730	
Time from infection	**− **0.02	[**− **0.05, 0.01]	**− **0.13	[**− **0.37, 0.08]	0.382	
Age	0.08	[**− **0.36, 0.51]	0.06	[**− **0.25, 0.30]	0.690	
						*R*^*2*^ = 0.402 *R*^*2*^adj. = 0.294 95% CI [0.28,0.73] FDR-adj. *p* = 0.006

*b* represents unstandardized regression weights. *beta* indicates the standardized regression weights. A significant (in bold) *b*-weight indicates the beta-weight is also significant. Square brackets are used to enclose the lower and upper limits of bootstrapped (1,000 samples) confidence intervals. *LL* and *UL* indicate the lower and upper limits of a confidence interval, respectively. FDR-adjusted model fit *p*-values. VPS = visual processing speed* C* (letters/second); PUI = pupillary unrest index (mm/min): Mental fatigue = Fatigue Assessment Scale (FAS, German version) mental fatigue subscore; Depression = Hospital Anxiety and Depression Scale (HADS-D, German version) depression subscore; Sleepiness = Epworth Sleepiness Scale score (ESS, German version); Time from (SARS-Cov2) infection (days); Age (years). Multiple regression analysis was conducted within the post-COVID patient group (*n* = 40)

## Discussion

The present study’s results support the hypothesis that arousal is reduced in post-COVID patients with subjective cognitive dysfunction. It was found that VPS, as a proxy for tonic alertness, is reduced in post-COVID patients compared to healthy controls. Second, such reduced tonic alertness was associated with indices of arousal on the neurophysiological and subjective experience level. That is, VPS was predicted by the level of central nervous activation measured through PUI and by the level of mental fatigue measured through self-rating.

VPS as a latent parameter reflecting tonic alertness according to the underlying theoretical TVA framework [13] was reduced in post-COVID patients compared to healthy controls. To the best of our knowledge, this is the first evidence of tonic alertness dysfunction in post-COVID patients measured in a process-pure manner. Based on the non-speeded performance measure and the independent mathematical modelling, we can interpret the effect of reduced VPS as perceptual (attentional) slowing which is independent of potential changes in motor capabilities and controlled for the potential influence of other attentional parameters that might affect report performance. According to the neural interpretation of TVA [12, 14], VPS reduction reflects a downscaled activation level in neurons involved in object coding, therefore, expressing hypoarousal of the brain.

As VPS estimation is based on performance changes across exposure durations that are extremely brief and vary from each other to a degree that is not detectable for the participant, the measure of VPS is less prone to possible aggravation tendencies than “classic”, simple-response-time-based measures of tonic alertness. Notably, a systematical increase of whole report performance highly predictable for the estimated TVA parameters was found in both groups. The resulting high goodness of fit between observed and estimated values that did not differ between groups indicate that patients delivered valid performance levels. Any attempts to simulate or aggravate a deficit would have resulted in less systematic performance curves, and therefore less optimal goodness of fit.

VPS is a basic cognitive function determining performance in diverse cognitive tasks, influencing global cognition [23, 52] and functional independence in aging individuals [51]. Therefore, based on our finding of perceptual (attentional) slowing indicative of hypoarousal, it is not astonishing that patients with neurological post-COVID syndrome show considerable long-term problems with respect to reintegration into work and societal life [24, 55]

Aside from reduced VPS, post-COVID patients also showed lower vSTM capacity* K* and higher visual perceptual threshold *t0* compared to healthy controls. These incidental findings might, however, represent secondary deficits also resulting from hypoarousal. Given that some of the patients suffered from severe (< 10 letters/s), rather than just moderate, slowing of VPS during the brief exposure durations used in the whole report, they might have simply not been able to fill up their vSTM store. Thus, in TVA terms, available slots in vSTM might have remained empty resulting in reduced vSTM capacity *K* values. This assumption is supported by the fact that vSTM capacity *K* was strongly [19] related to VPS in patients, while in healthy controls their relationship was merely moderate [19] (as is typically found in healthy participants, e.g. [28]. In line with this, prior patient studies have shown that severe slowing of VPS is related to deficits in the simultaneous perception of multiple visual objects [47, 62]. Such deficits might also prevail in the more severely slowed post-COVID patients. This should be tested in future studies. Note that we also found that the relationship between vSTM capacity *K* and PUI was fully explained by VPS. Furthermore, delayed processing of visual information, indicated by higher *t0* values in post-COVID patients, might also represent a secondary deficit reflecting hypoarousal. For example, it was documented that, apart from VPS, also visual threshold is affected by arousal variations [56]. We thus assume that slowing in VPS represents a core attentional deficit reflecting hypoarousal in post-COVD patients. If our conclusions are correct, enhancement of arousal could not only improve VPS but also lead to enhanced vSTM capacity and lowered visual perceptual threshold. This should be tested in future studies.

In a regression analysis, we found that VPS in post-COVID patients was predicted by PUI, as a proxy for central nervous activation, and by self-rated mental fatigue, as a proxy for the subjective experience of post-COVID patients. Both predictors explained 34% of variance of tonic alertness reduction, yielding a large effect with *f*^2^ = 0.51 [19]. This relationship was exclusive, as none of the potentially relevant covariates, i.e. depressive symptoms, sleepiness, time from infection nor age were valid predictors for VPS. Post-COVID patients with lower VPS are characterized by higher PUI, corroborating the relation between tonic alertness and central nervous activation. Notably, higher PUI levels are associated with smaller average PD, confirming that higher PUI actually reflects low brain arousal [35, 57, 59]. Furthermore, post-COVID patients with lower VPS have a more pronounced feeling of mental fatigue, implying the clinical relevance of the tonic alertness reduction with respect to the patient-related impairment level. It is noteworthy that VPS shows substantial variance in healthy participants (e.g. [28]), as can also be seen in our control group. Thus, the amount of tonic alertness *reduction* that is explained by pupillary unrest and self-rated mental fatigue in post-COVID patients might even be higher, although this is not directly testable without the availability of pre-infection VPS data. According to our data, VPS, therefore, represents a valid proxy of arousal that relates to both the level of central nervous activation as well as arousal on the subjective experience level in post-COVID patients.

Once identified, VPS could serve as a target as well as an efficacy marker in future systematic treatment studies targeting hypoarousal. Given the substantial amount of patients affected worldwide [18], scalable, digital treatments, such as computerized cognitive training, might be useful as digital alertness training was shown to improve VPS in older individuals [54].

We cannot directly infer the underlying pathomechanisms of arousal dysfunction in post-COVID patients in our study. However, our results point towards possible neurostructures and -modulators. As tonic alertness regulation depends on input from wakefulness-promoting monoaminergic systems including the noradrenergic locus coeruleus system [2, 45, 58, 64], post-infectious brainstem alterations might lead to decreased cortical activation resulting in slowed information processing speed and feeling of fatigue [10]. Notably, in multiple sclerosis patients, disrupted brainstem monoaminergic pathways have been associated with cognitive fatigue [16] and reduced intra-brainstem connectivity correlated with symptom severity in patients suffering from chronic fatigue syndrome [5]. Brainstem hypometabolism in a case series of three post-COVID patients suffering from brain fog [37] and changes in monoamine levels in SARS-Cov2-recovered hACE2 mice [67] have been reported. The brainstem expresses angiotensin-converting enzyme 2, which could therein explain SARS-Cov2 tropism [81]. Departing from our results demonstrating hypoarousal, future studies implementing appropriate neuroimaging procedures in post-COVID patients can more directly assess the underlying specific neurostructural and –modulatory mechanisms.

Incidentally, we found that self-rated mental fatigue and depressive symptoms were intercorrelated in our post-COVID patient group, indicating that mental fatigue imposes a significant stressor leading to higher psychological strain, or vice versa.

The present study has critical limitations. The sample size is relatively small. The cross-sectional design cannot inform about the long-term trajectories of VPS, mental fatigue and PUI and a longitudinal analysis should complement our study. Furthermore, due to hospital restriction policies during the pandemic with limited access to the JUH Interdisciplinary Centre for Sleep and Ventilatory Medicine, we do not have healthy control data of pupillary unrest. Moreover, we do not report (cognitive or pupillometric) measures of phasic alertness indicating the ability to react to external stimulation, such as warning stimuli or light, with a fast increase of the arousal/readiness level [58, 72], due to our interest in intrinsic, tonic alertness reflecting the overall arousal state of the brain. Future studies focusing on the phasic alertness system could combine on-task pupillometry (e.g. event-evoked pupil dilation) with respective effects on visual processing speed, as has been done in healthy individuals [56].

In sum, this is the first study to systematically investigate arousal in post-COVID patients. Our results indicate that processing speed is hampered in post-COVID patients by hypoarousal of the brain. Furthermore, the reduction in processing speed relates to and therefore objectifies mental fatigue as a core subjective clinical complaint in post-COVID syndrome. Future studies should address underlying pathomechanisms and the potential of interventions targeting hypoarousal.

### Supplementary Information

Below is the link to the electronic supplementary material.

Supplementary file1 (DOCX 4471 KB)

## Data Availability

Upon a reasonable request, the corresponding author can provide the data supporting the findings of this study.

## References

[1] Arditi A (2005). Improving the design of the letter contrast sensitivity test. Invest Ophthalmol Vis Sci.

[2] Aston-Jones G, Cohen JD (2005). Adaptive gain and the role of the locus coeruleus-norepinephrine system in optimal performance. J Comp Neurol.

[3] Augustin M, Schommers P, Stecher M, Dewald F, Gieselmann L, Gruell H, Horn C, Vanshylla K, Cristanziano VD, Osebold L, Roventa M, Riaz T, Tschernoster N, Altmueller J, Rose L, Salomon S, Priesner V, Luers JC, Albus C, Rosenkranz S, Gathof B, Fatkenheuer G, Hallek M, Klein F, Suarez I, Lehmann C (2021). Post-COVID syndrome in non-hospitalised patients with COVID-19: a longitudinal prospective cohort study. Lancet Reg Health Eur.

[4] Badenoch JB, Rengasamy ER, Watson C, Jansen K, Chakraborty S, Sundaram RD, Hafeez D, Burchill E, Saini A, Thomas L, Cross B, Hunt CK, Conti I, Ralovska S, Hussain Z, Butler M, Pollak TA, Koychev I, Michael BD, Holling H, Nicholson TR, Rogers JP, Rooney AG (2022). Persistent neuropsychiatric symptoms after COVID-19: a systematic review and meta-analysis. Brain Commun.

[5] Barnden LR, Shan ZY, Staines DR, Marshall-Gradisnik S, Finegan K, Ireland T, Bhuta S (2019). Intra brainstem connectivity is impaired in chronic fatigue syndrome. Neuroimage Clin.

[6] Bloch KE, Schoch OD, Zhang JN, Russi EW (1999). German version of the Epworth Sleepiness Scale. Respiration.

[7] Bloxham C, Dick D, Moore M (1987). Reaction times and attention in Parkinson's disease. J Neurol Neurosurg Psychiatry.

[8] Brown LA, Ballentine E, Zhu Y, McGinley EL, Pezzin L, Abramoff B (2022). The unique contribution of depression to cognitive impairment in Post-Acute Sequelae of SARS-CoV-2 infection. Brain Behav Immun Health.

[9] Bruno RL, Creange S, Zimmerman JR, Frick NM (1998). Elevated plasma prolactin and EEG slow wave power in post-polio fatigue: Implications for a dopamine deficiency underlying post-viral fatigue syndromes. J Chronic Fatigue Syndrome.

[10] Bruno RL, Creange SJ, Frick NM (1998). Parallels between post-polio fatigue and chronic fatigue syndrome: a common pathophysiology?. Am J Med.

[11] Bundesen C (1990). A theory of visual attention. Psychol Rev.

[12] Bundesen C, Habekost T, Kyllingsbaek S (2005). A neural theory of visual attention: bridging cognition and neurophysiology. Psychol Rev.

[13] Bundesen C, Vangkilde S, Habekost T (2015). Components of visual bias: a multiplicative hypothesis. Ann N Y Acad Sci.

[14] Bundesen C, Vangkilde S, Petersen A (2015). Recent developments in a computational theory of visual attention (TVA). Vision Res.

[15] Bungenberg J, Humkamp K, Hohenfeld C, Rust MI, Ermis U, Dreher M, Hartmann NK, Marx G, Binkofski F, Finke C, Schulz JB, Costa AS, Reetz K (2022). Long COVID-19: objectifying most self-reported neurological symptoms. Ann Clin Transl Neurol.

[16] Carandini T, Mancini M, Bogdan I, Rae CL, Barritt AW, Sethi A, Harrison N, Rashid W, Scarpini E, Galimberti D, Bozzali M, Cercignani M (2021). Disruption of brainstem monoaminergic fibre tracts in multiple sclerosis as a putative mechanism for cognitive fatigue: a fixel-based analysis. Neuroimage Clin.

[17] Ceban F, Ling S, Lui LMW, Lee Y, Gill H, Teopiz KM, Rodrigues NB, Subramaniapillai M, Di Vincenzo JD, Cao B, Lin K, Mansur RB, Ho RC, Rosenblat JD, Miskowiak KW, Vinberg M, Maletic V, McIntyre RS (2022). Fatigue and cognitive impairment in Post-COVID-19 syndrome: a systematic review and meta-analysis. Brain Behav Immun.

[18] Chen C, Haupert SR, Zimmermann L, Shi X, Fritsche LG, Mukherjee B (2022). Global Prevalence of Post-Coronavirus Disease 2019 (COVID-19) Condition or Long COVID: A Meta-Analysis and Systematic Review. J Infect Dis.

[19] Cohen J (1988) Statistical power analysis for the behavioral sciences. Lawrence Erlbaum Associates, New York

[20] R Core Team (2021) R: A language and environment for statistical computing. R Foundation for Statistical Computing, Vienna [Computer software]. https://www.R-project.org/

[21] Crivelli L, Palmer K, Calandri I, Guekht A, Beghi E, Carroll W, Frontera J, Garcia-Azorin D, Westenberg E, Winkler AS, Mangialasche F, Allegri RF, Kivipelto M (2022). Changes in cognitive functioning after COVID-19: a systematic review and meta-analysis. Alzheimers Dement.

[22] Danker-Hopfe H, Kraemer S, Dorn H, Schmidt A, Ehlert I, Herrmann WM (2001). Time-of-day variations in different measures of sleepiness (MSLT, pupillography, and SSS) and their interrelations. Psychophysiology.

[23] Deary IJ, Johnson W, Starr JM (2010). Are processing speed tasks biomarkers of cognitive aging?. Psychol Aging.

[24] Delgado-Alonso C, Cuevas C, Oliver-Mas S, Diez-Cirarda M, Delgado-Alvarez A, Gil-Moreno MJ, Matias-Guiu J, Matias-Guiu JA (2022). Fatigue and cognitive dysfunction are associated with occupational status in post-COVID syndrome. Int J Environ Res Public Health.

[25] Desimone R, Duncan J (1995). Neural mechanisms of selective visual attention. Annu Rev Neurosci.

[26] Espeseth T, Vangkilde SA, Petersen A, Dyrholm M, Westlye LT (2014). TVA-based assessment of attentional capacities-associations with age and indices of brain white matter microstructure. Front Psychol.

[27] Faul F, Erdfelder E, Lang AG, Buchner A (2007). G*Power 3: a flexible statistical power analysis program for the social, behavioral, and biomedical sciences. Behav Res Methods.

[28] Finke K, Bublak P, Krummenacher J, Kyllingsbaek S, Muller HJ, Schneider WX (2005). Usability of a theory of visual attention (TVA) for parameter-based measurement of attention I: evidence from normal subjects. J Int Neuropsychol Soc.

[29] Giszas B, Trommer S, Schussler N, Rodewald A, Besteher B, Bleidorn J, Dickmann P, Finke K, Katzer K, Lehmann-Pohl K, Lemhofer C, Pletz MW, Puta C, Quickert S, Walter M, Stallmach A, Reuken PA (2022) Post-COVID-19 condition is not only a question of persistent symptoms: structured screening including health-related quality of life reveals two separate clusters of post-COVID. Infection 51:365–377. 10.1007/s15010-022-01886-9 10.1007/s15010-022-01886-9 PMC9307219 35869353

[30] Gobbelé R, Waberski TD, Thyerlei D, Thissen M, Fimm B, Klostermann F, Curio G, Buchner H (2007). Human high frequency somatosensory evoked potential components are refractory to circadian modulations of tonic alertness. J Clin Neurophysiol.

[31] Graham EL, Clark JR, Orban ZS, Lim PH, Szymanski AL, Taylor C, DiBiase RM, Jia DT, Balabanov R, Ho SU, Batra A, Liotta EM, Koralnik IJ (2021). Persistent neurologic symptoms and cognitive dysfunction in non-hospitalized Covid-19 "long haulers". Ann Clin Transl Neurol.

[32] Hancock PA (1989). A dynamic model of stress and sustained attention. Hum Factors.

[33] Hayes AF (2022). Introduction to mediation, moderation, and conditional process analysis: a regression-based approach.

[34] Hayes AF, Rockwood NJ (2017). Regression-based statistical mediation and moderation analysis in clinical research: observations, recommendations, and implementation. Behav Res Ther.

[35] Henson DB, Emuh T (2010). Monitoring vigilance during perimetry by using pupillography. Invest Ophthalmol Vis Sci.

[36] Herrmann-Lingen C, Buss U, Snaith RP (2011). Hospital anxiety and depression scale, deutsche version (HADS-D).

[37] Hugon J, Queneau M, Sanchez Ortiz M, Msika EF, Farid K, Paquet C (2022). Cognitive decline and brainstem hypometabolism in long COVID: a case series. Brain Behav.

[38] Jain S, Siegle GJ, Gu C, Moore CG, Ivanco LS, Studenski S, Greenamyre JT, Steinhauer SR (2011). Pupillary unrest correlates with arousal symptoms and motor signs in Parkinson disease. Mov Disord.

[39] Jennings G, Monaghan A, Xue F, Duggan E, Romero-Ortuño R (2022). Comprehensive clinical characterisation of brain fog in adults reporting long COVID symptoms. J Clin Med.

[40] Kyllingsbaek S (2006). Modeling visual attention. Behav Res Methods.

[41] Lemhofer C, Sturm C, Loudovici-Krug D, Best N, Gutenbrunner C (2021). The impact of Post-COVID-Syndrome on functioning—results from a community survey in patients after mild and moderate SARS-CoV-2-infections in Germany. J Occup Med Toxicol.

[42] Lowenstein O, Feinberg R, Loewenfeld IIE (1963). Pupillary movements during acute and chronic fatigue: a new test for the objective evaluation of tiredness. Investig Ophthalmol.

[43] Lüdtke H, Wilhelm B, Adler M, Schaeffel F, Wilhelm H (1998). Mathematical procedures in data recording and processing of pupillary fatigue waves. Vision Res.

[44] Maccora J, Manousakis JE, Anderson C (2019). Pupillary instability as an accurate, objective marker of alertness failure and performance impairment. J Sleep Res.

[45] Maness EB, Burk JA, McKenna JT, Schiffino FL, Strecker RE, McCoy JG (2022). Role of the locus coeruleus and basal forebrain in arousal and attention. Brain Res Bull.

[46] Michielsen HJ, De Vries J, Van Heck GL (2003). Psychometric qualities of a brief self-rated fatigue measure: the Fatigue Assessment Scale. J Psychosom Res.

[47] Neitzel J, Ortner M, Haupt M, Redel P, Grimmer T, Yakushev I, Drzezga A, Bublak P, Preul C, Sorg C, Finke K (2016). Neuro-cognitive mechanisms of simultanagnosia in patients with posterior cortical atrophy. Brain.

[48] Neumann M, Sterr A, Claros-Salinas D, Gütler R, Ulrich R, Dettmers C (2014). Modulation of alertness by sustained cognitive demand in MS as surrogate measure of fatigue and fatigability. J Neurol Sci.

[49] Niepel G, Bibani RH, Vilisaar J, Langley RW, Bradshaw CM, Szabadi E, Constantinescu CS (2013). Association of a deficit of arousal with fatigue in multiple sclerosis: effect of modafinil. Neuropharmacology.

[50] Olbrich S, Sander C, Jahn I, Eplinius F, Claus S, Mergl R, Schönknecht P, Hegerl U (2012). Unstable EEG-vigilance in patients with cancer-related fatigue (CRF) in comparison to healthy controls. World J Biol Psychiatry.

[51] Owsley C (2013). Visual processing speed. Vision Res.

[52] Park DC, Reuter-Lorenz P (2009). The adaptive brain: aging and neurocognitive scaffolding. Annu Rev Psychol.

[53] Paus T, Zatorre RJ, Hofle N, Caramanos Z, Gotman J, Petrides M, Evans AC (1997). Time-related changes in neural systems underlying attention and arousal during the performance of an auditory vigilance task. J Cogn Neurosci.

[54] Penning MD, Ruiz-Rizzo AL, Redel P, Muller HJ, Salminen T, Strobach T, Behrens S, Schubert T, Sorg C, Finke K (2021). Alertness training increases visual processing speed in healthy older adults. Psychol Sci.

[55] Perlis RH, Lunz Trujillo K, Safarpour A, Santillana M, Ognyanova K, Druckman J, Lazer D (2023). Association of post-COVID-19 condition symptoms and employment status. JAMA Netw Open.

[56] Petersen A, Petersen AH, Bundesen C, Vangkilde S, Habekost T (2017). The effect of phasic auditory alerting on visual perception. Cognition.

[57] Phillips M, Szabadi E, Bradshaw C (2000). Comparison of the effects of clonidine and yohimbine on spontaneous pupillary fluctuations in healthy human volunteers. Psychopharmacology.

[58] Posner MI (2008). Measuring alertness. Ann N Y Acad Sci.

[59] Ranzijn R, Lack L (1997). The pupillary light reflex cannot be used to measure sleepiness. Psychophysiology.

[60] Regen F, Dorn H, Danker-Hopfe H (2013). Association between pupillary unrest index and waking electroencephalogram activity in sleep-deprived healthy adults. Sleep Med.

[61] Rudroff T, Fietsam AC, Deters JR, Bryant AD, Kamholz J (2020). Post-COVID-19 fatigue: potential contributing factors. Brain Sci.

[62] Ruiz-Rizzo AL, Sorg C, Napiorkowski N, Neitzel J, Menegaux A, Muller HJ, Vangkilde S, Finke K (2019). Decreased cingulo-opercular network functional connectivity mediates the impact of aging on visual processing speed. Neurobiol Aging.

[63] Saif DS, Ibrahem RA, Eltabl MA (2022). Prevalence of peripheral neuropathy and myopathy in patients post-COVID-19 infection. Int J Rheum Dis.

[64] Samuels ER, Szabadi E (2008). Functional neuroanatomy of the noradrenergic locus coeruleus: its roles in the regulation of arousal and autonomic function part I: principles of functional organisation. Curr Neuropharmacol.

[65] Sander C, Hensch T, Wittekind DA, Bottger D, Hegerl U (2015). Assessment of wakefulness and brain arousal regulation in psychiatric research. Neuropsychobiology.

[66] Santoyo-Mora M, Villaseñor-Mora C, Cardona-Torres LM, Martínez-Nolasco JJ, Barranco-Gutiérrez AI, Padilla-Medina JA, Bravo-Sánchez MG (2022). COVID-19 long-term effects: Is there an impact on the simple reaction time and alternative-forced choice on recovered patients?. Brain Sci.

[67] Schou T, Wegener G, Joca S, Bay-Richter C (2022). Long-COVID’-a neuroinflammatory disease. Brain Behave Immunity.

[68] Sivan M, Taylor S (2020). NICE guideline on long covid. BMJ.

[69] Snaith RP, Zigmond AS (1986). The hospital anxiety and depression scale. Br Med J (Clin Res Ed).

[70] Sperling G (1960). The information available in brief visual presentations. Psychol Monogr Gen Appl.

[71] Stallmach A, Kesselmeier M, Bauer M, Gramlich J, Finke K, Fischer A, Fleischmann-Struzek C, Heutelbeck A, Katzer K, Mutschke S, Pletz MW, Quickert S, Reinhart K, Stallmach Z, Walter M, Scherag A, Reuken PA (2022). Comparison of fatigue, cognitive dysfunction and psychological disorders in post-COVID patients and patients after sepsis: is there a specific constellation?. Infection.

[72] Sturm W, Willmes K (2001). On the functional neuroanatomy of intrinsic and phasic alertness. Neuroimage.

[73] Tanriverdi A, Savci S, Kahraman BO, Ozpelit E (2022). Extrapulmonary features of post-COVID-19 patients: muscle function, physical activity, mood, and sleep quality. Ir J Med Sci.

[74] Taquet M, Geddes JR, Husain M, Luciano S, Harrison PJ (2021). 6-month neurological and psychiatric outcomes in 236 379 survivors of COVID-19: a retrospective cohort study using electronic health records. Lancet Psychiatry.

[75] Ulke C, Surova G, Sander C, Engel C, Wirkner K, Jawinski P, Hensch T, Hegerl U (2020). Fatigue in cancer and neuroinflammatory and autoimmune disease: CNS arousal matters. Brain Sci.

[76] Weinges-Evers N, Brandt AU, Bock M, Pfueller CF, Dorr J, Bellmann-Strobl J, Scherer P, Urbanek C, Boers C, Ohlraun S, Zipp F, Paul F (2010). Correlation of self-assessed fatigue and alertness in multiple sclerosis. Mult Scler.

[77] Wilhelm B, Giedke H, Lüdtke H, Bittner E, Hofmann A, Wilhelm H (2001). Daytime variations in central nervous system activation measured by a pupillographic sleepiness test. J Sleep Res.

[78] Wilhelm BJ (2008). Pupillography for the assessment of driver sleepiness. Klin Monbl Augenheilkd.

[79] Williams VSJ, Jones LV, Tukey JW (1999). Controlling error in multiple comparisons, with examples from state-to-state differences in educational achievement. J Educ Behav Stat.

[80] Yerkes RM, Dodson JD (1908). The relation of strength of stimulus to rapidity of habit-formation. J Comp Neurol Psychol.

[81] Yong SJ (2021). Persistent brainstem dysfunction in long-COVID: a hypothesis. ACS Chem Neurosci.

