# Supplementary material for: A hypoarousal model of neurological post-COVID syndrome: the relation between mental fatigue, the level of central nervous activation and cognitive processing speed
Source: J Neurol. 2023 Jun 25;270(10):4647–60. doi: 10.1007/s00415-023-11819-7 (PMC10511382; doi:10.1007/s00415-023-11819-7)

# **A hypoarousal model of neurological post-COVID syndrome: the relation between mental fatigue, the level of central nervous activation and cognitive processing speed**

Eva Maria Martin^1^, Sven Rupprecht^1,2^, Simon Schrenk^1^, Fabian Kattlun^1^, Isabelle Utech^1^, Monique Radscheidt^1,2^, Stefan Brodoehl^1^, Matthias Schwab^1^, Philipp A. Reuken^3,^ Andreas Stallmach^3^, Thomas Habekost^4^ & Kathrin Finke^1,5,6^

^1^Department of Neurology, Jena University Hospital, Jena,Germany, ^2^Interdisciplinary Centre for Sleep and Ventilatory Medicine, Jena University Hospital, Jena, Germany, ^3^Department of Internal Medicine IV (Gastroenterology, Hepatology and Infectious Diseases), Jena University Hospital, Jena, Germany, ^4^Center of Visual Cognition, University of Copenhagen, Copenhagen, Denmark ^5^Center for Sepsis Control and Care, Jena University Hospital, Jena, Germany ^6^Department of Psychology, Ludwig-Maximilians-University Munich, Munich, Germany

**Supplementary Information**

1. **Methods – Technical details of TVA-based parameter assessment**

### *Assessment of visual processing speed C (VPS) and other visual attention parameters*

Both paradigms were run using Matlab (Version R2020a 9.8.0.1323502), PsychToolbox and presented on a 24inch/61cm-wide monitor screen (1080p resolution, 100 Hz refresh rate) on a black background. Distance to the computer monitor was kept at 60 cm by use of a chin rest. Each trial of letter presentation in the whole and partial report paradigms started with a 1000 ms presentation of a central fixation point (diameter: 1 cm) at a screen with a standardized luminance set at 0.49cd/m2 in a dimly lit room. Masks consisted of squares with a checkerboard pattern in red and blue fully covering the letters in per participant.

After each trial block, the experimenter was automatically informed about the percentage of accurately reported letters. The aim was to achieve an accuracy level lying between 70% to 90%. If the accuracy level of reported letters was too high (>90%), participants were asked to report letters that they believed to have seen without being 100% certain about it. If the accuracy level was too low (<70%), participants were asked to only report letters they were absolutely certain about. The completion of the whole report and partial report tasks took between 45 to 60 m.

#### Whole report paradigm

To find the five adequate exposure durations, for a given participant a pretest phase consisting of 4 practice blocks with 12 trials were carried out per block. In order to prevent discouragement, one masked calibration trial was accompanied by 2 “easier” trials with (i.e., one longer and one unmasked trial), with the three trials forming a triplet. Trials within these triplets were randomized. Only the calibration trial was critical for exposure duration adjustment. Calibration trials started at 80ms and, if the subject was able to identify at least one target correctly, the exposure duration would be reduced by 10ms until reaching the lowest individual threshold, where no letter could be reported anymore. This threshold was used to identify four additional adjusted longer exposure durations according to a Bayesian adaptive staircase paradigm, resulting in 5 masked adjusted exposure durations, e.g., 10, 20, 40, 90, and 200 ms. The exposure durations were ranging from 10ms to 250ms in the healthy control group and from 10 ms to 260 ms in the post-COVID patient group.

Additionally, in two unmasked conditions, letters were presented for the second shortest and the longest exposure duration. In unmasked trials, a retinal afterimage of the presented letters emerged which extended the effective exposure durations by a constant duration represented by parameter μ (in ms) [58]. The effective exposure duration in unmasked displays *μ,* was also estimated. This parameter serves the valid estimation of the parameters of interest but apart from this, is of no further relevance in the present study.

#### Partial report paradigm

To find the individually adjusted exposure duration, calibration trials were carried out. Calibration started with an exposure duration of 80ms. Exposure duration was decreased by 10ms if participants were able to report two letters in a T-T-condition or was increased by 10ms if none of the two targets in T-T-condition could be named. This was done so until, on average, one letter per trial could be named correctly. The aim was to adjust individual exposure duration to a speed at which accuracy was above 50% for T-T-condition and between 70% and 90% for T-condition. If this could not be achieved during the first 24 trials, exposure duration would be readjusted manually by the experimenter and another 24 calibration trials were run until the he exposure duration for identifying letters in a dual-target condition was adjusted based on the participant's ability to identify the letters. In the pretest, a total of 24 calibration trials were used to determine one individually adjusted exposure duration, ranging from 40ms to 150ms in the healthy control group and from 60 ms to 230 ms in the post-COVID patient group.

#### Estimation of visual attention parameters

From the partial report accuracy across different conditions, attentional weights are derived for targets (wT) and distractors (wD). Parameter α is defined as the ratio of distractor to target weights (wD/wT) and reflects top-down efficacy, i.e., the ability to prioritize task-relevant over task-irrelevant information. Values of α close to 0 indicate a high selectivity, i.e., targets receive more weight than distractors while values of α close to 1 signify no selection. The partial report model predicted 58% of the variability of the observed mean scores for healthy control participants and 61% for post-COVID patients, indicating a satisfactory goodness of fit of the TVA model.

1. **Results**

### *Spearman’s rank correlations within the healthy control group (n = 40)*

|  | **VPS** | **Fatigue** | **Depression** | **Sleepiness** | **Age** | ***K*** | ***t0*** |
| --- | --- | --- | --- | --- | --- | --- | --- |
| **Fatigue** | -.209 |  |  |  |  |  |  |
| **Depression** | -.320 | .487^**^ |  |  |  |  |  |
| **Sleepiness** | -.174 | .339 | .523^**^ |  |  |  |  |
| **Age** | -.161 | .062 | .008 | .282 |  |  |  |
| ***K*** | .336^*^ | -.141 | -.369^*^ | -.113 | -.333^*^ |  |  |
| ***t0*** | -.385^*^ | .340^*^ | .095 | .057 | .278 | -.210 |  |
| ***α*** | .376^*^ | -.001 | -.080 | .080 | .052 | .005 | .027 |

*Note.* *indicates *p* < 0.05. ** indicates *p* <0 .01. VPS = visual processing speed *C* (letters/second); PUI = pupillary unrest index (mm/min): Mental fatigue = Fatigue Assessment Scale (FAS, German version); Depression = Hospital Anxiety and Depression Scale (HADS-D, German version) depression subscore; Sleepiness = Epworth Sleepiness Scale (ESS, German version) score; Time from (SARS-Cov2-) infection in days; Age in years; *K* = visual short term memory capacity (maximum number of letters); *t0* = visual perceptual threshold (in ms); *α* = top-down control (distractor/targets).

### *Graphic depiction of overlap and non-overlap of non-paramtric distributions for VPS and the other TVA parameters per group*

**Fig.6** Non-parametric distribution curves per group for VPS and the other TVA parameters

*
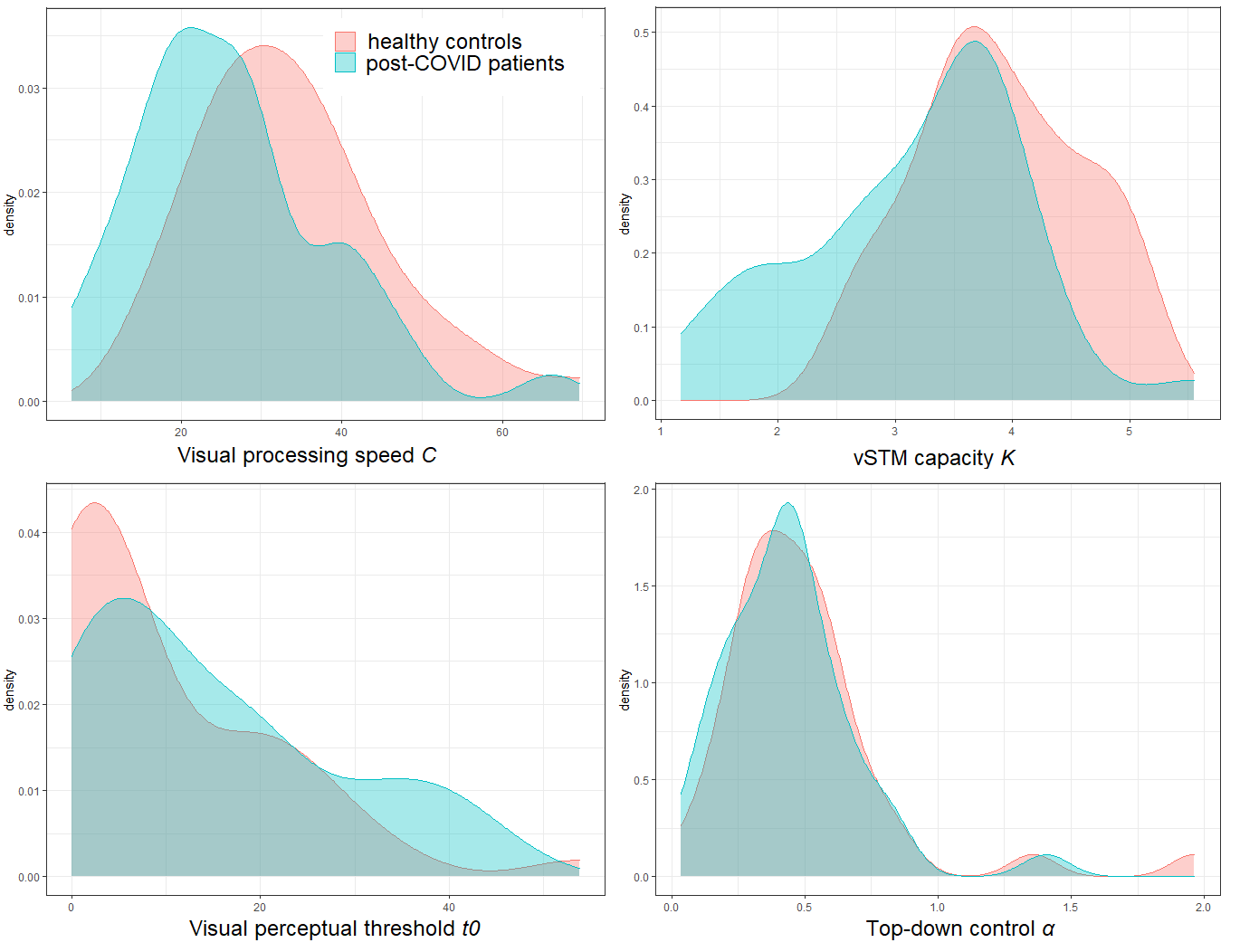
*

### *Mediation analysis*

Visual processing speed *C* (VPS) fully mediated the association between pupillary unrest index (PUI) and visual short term memory (vSTM) capacity *K*. Bootstrapping (1.000 samples) was applied. We found a non-significant direct effect of PUI on *K* (b1 = -0.130. *SE*1 = 0.094. 95% CI1 [LL.UL] = [-0.303. 0.073]) and a significant indirect effect (b2 = -0.089. *SE*2 = 0.066. 95% CI2[LL. UL] = [-0.250. -0.011]). Fig. 6 depicts the mediation path model. Every increase in PUI (mm/min) results in a decrease of vSTM capacity *K* (maximum number of letters). Such decrease is given through the positive effect of PUI on VPS (blue path) and negative effect of VPS on vSTM capacity *K* (red path).

**Fig.7:** Mediation model of pupillary unrest (PUI) on vSTM capacity *K* through visual processing speed *C* (VPS).


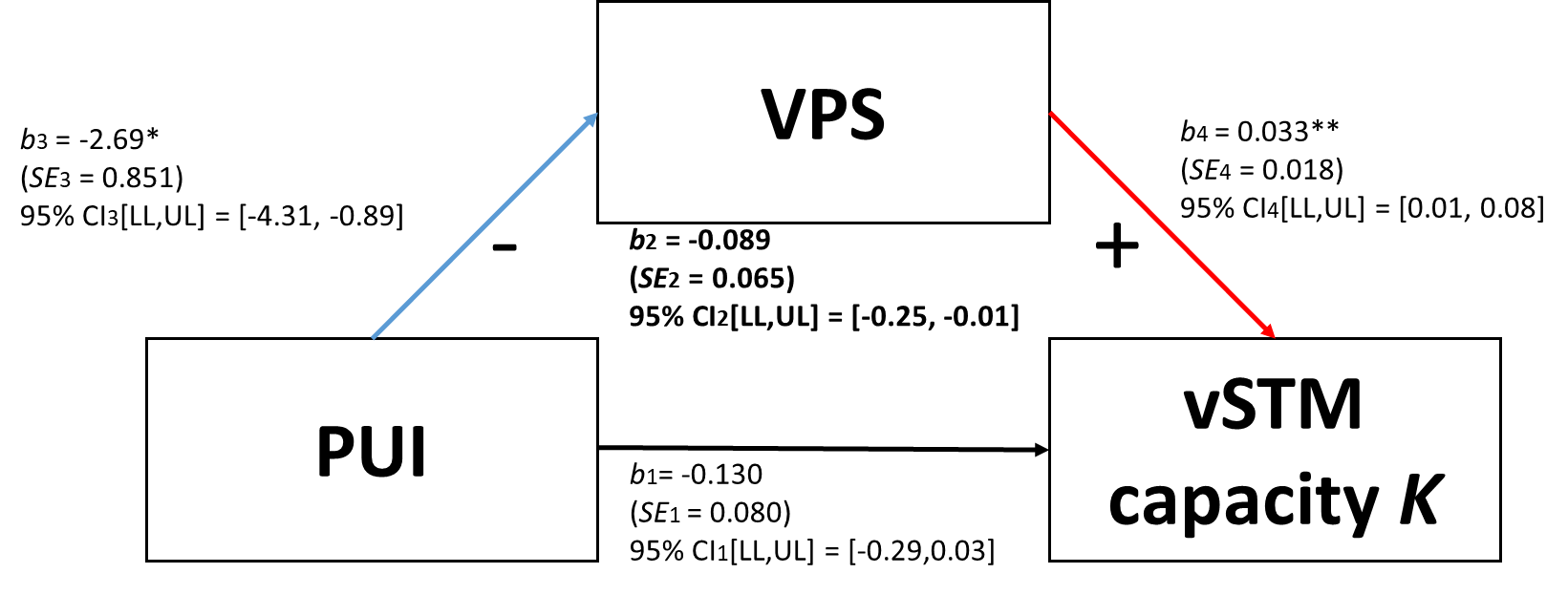


*Note*. Mediation path model of pupillary unrest (PUI) on vSTM capacity *K* through visual processing speed *C* (VPS). Bootstrapped results (1.000 samples). Unstandardized coefficients (b). standard errors (*SE*) and 95% confidence intervals (CI) of the particular effects (arrows) are depicted. blue indicating a significant negative and red indicating a significant positive effect. black indicating a non-significant effect. Bold *b2*. *SE2* and CI2 represent the significant indirect effect of interest. i.e. the effect of PUI on *K* through VPS.

### *Individual whole report performance (fitted curves) of all post-COVID patients and healthy control participants*

#### Fitted whole report performance Post-COVID patient curves


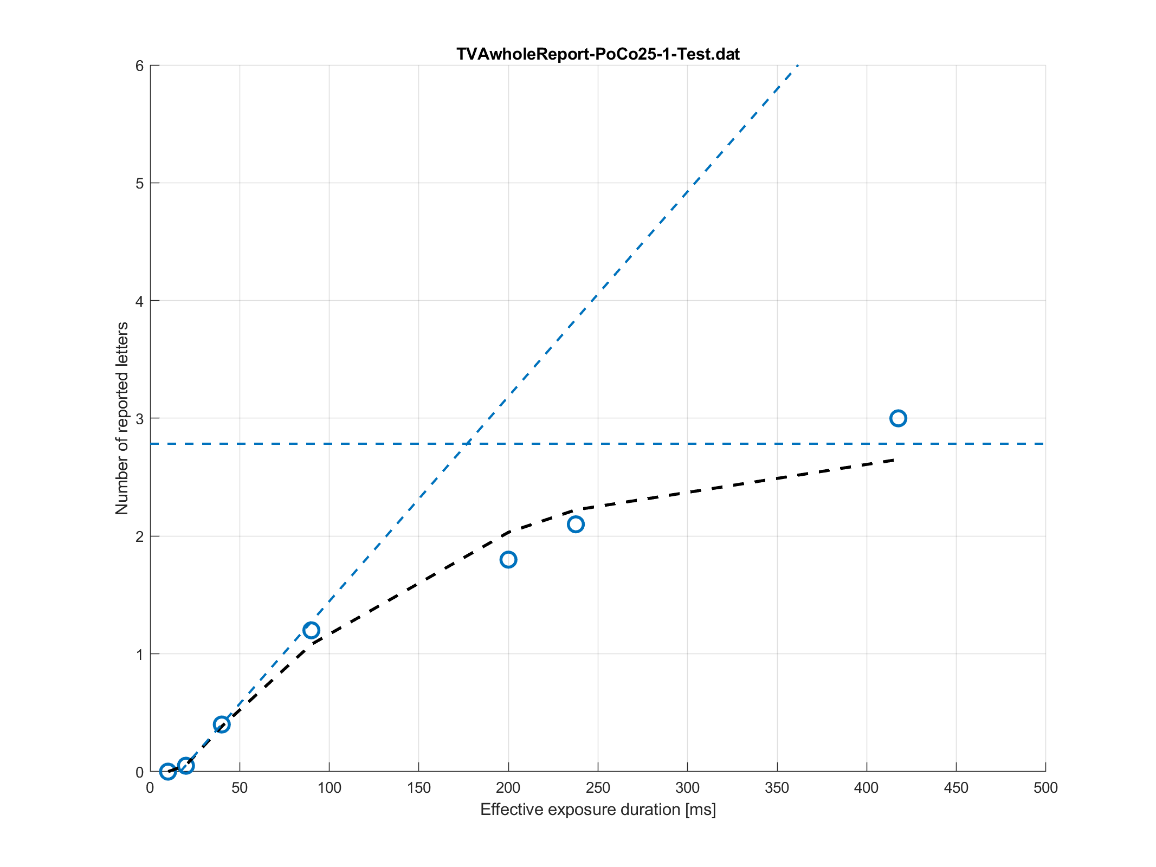

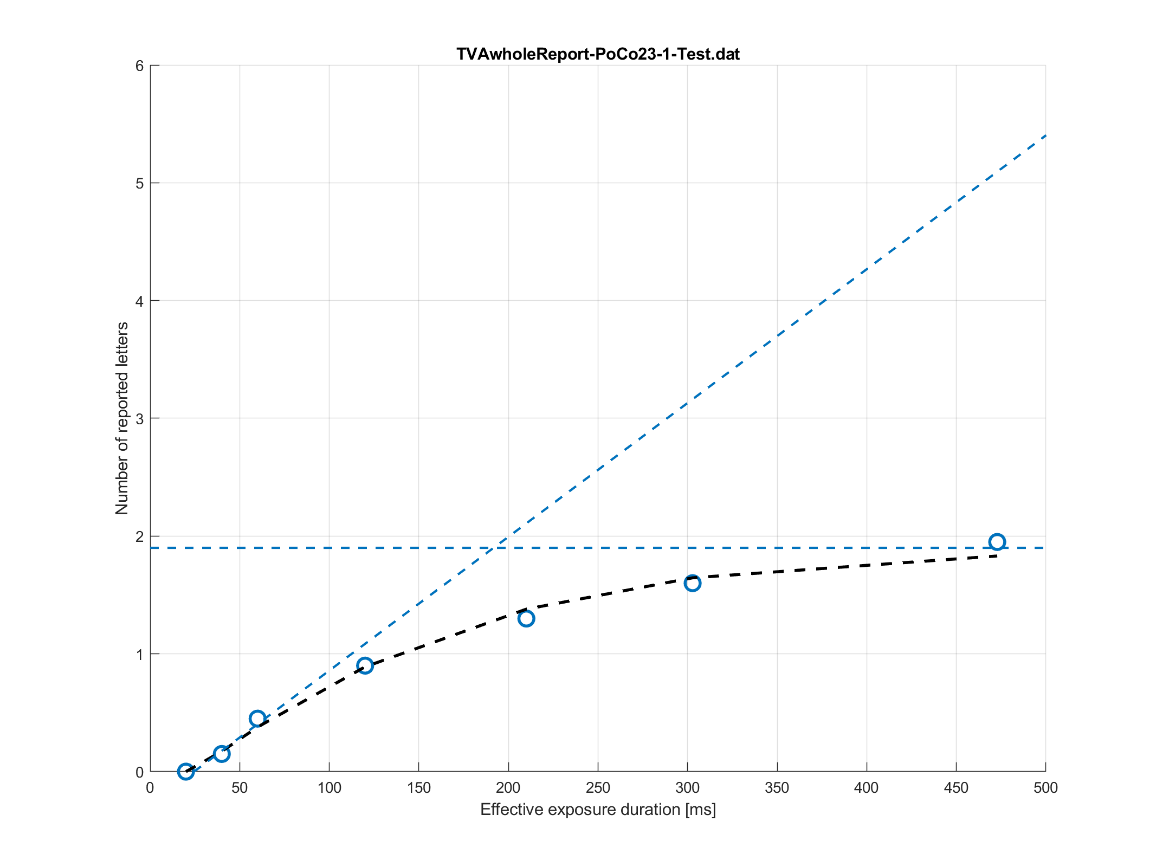

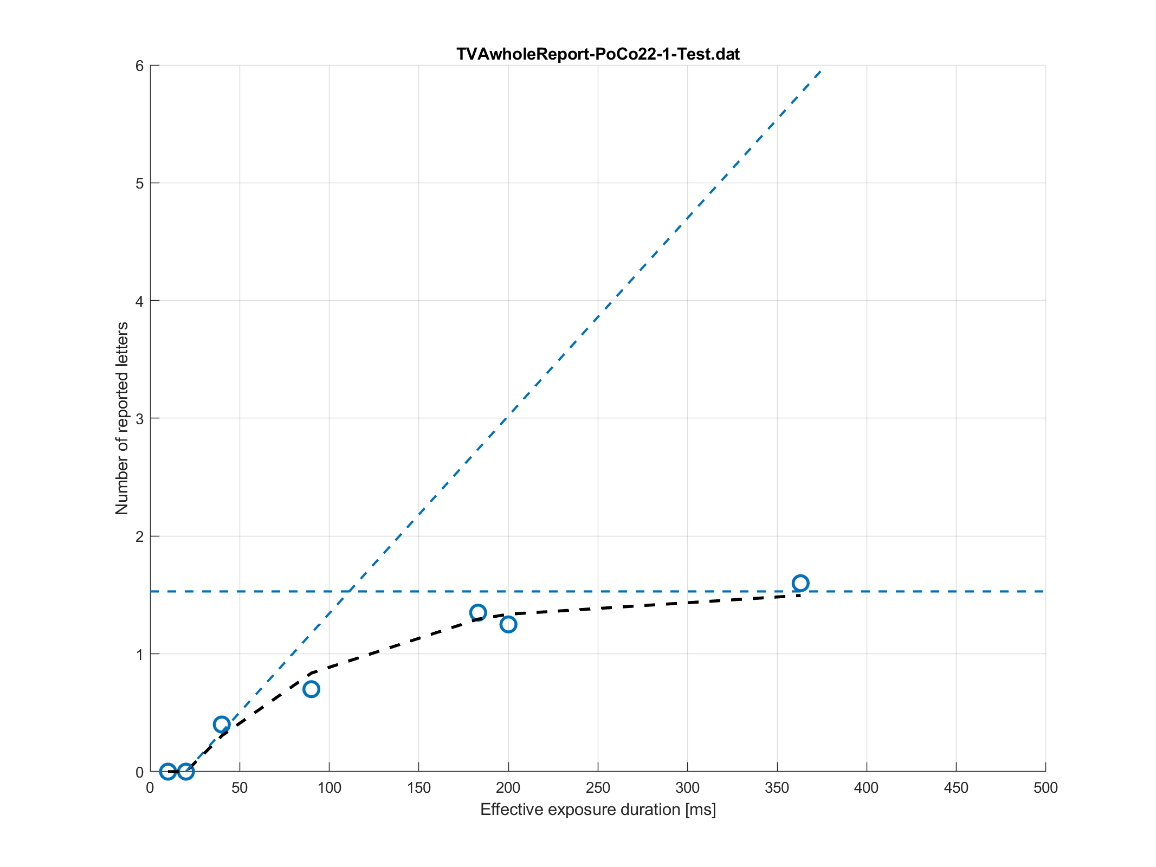

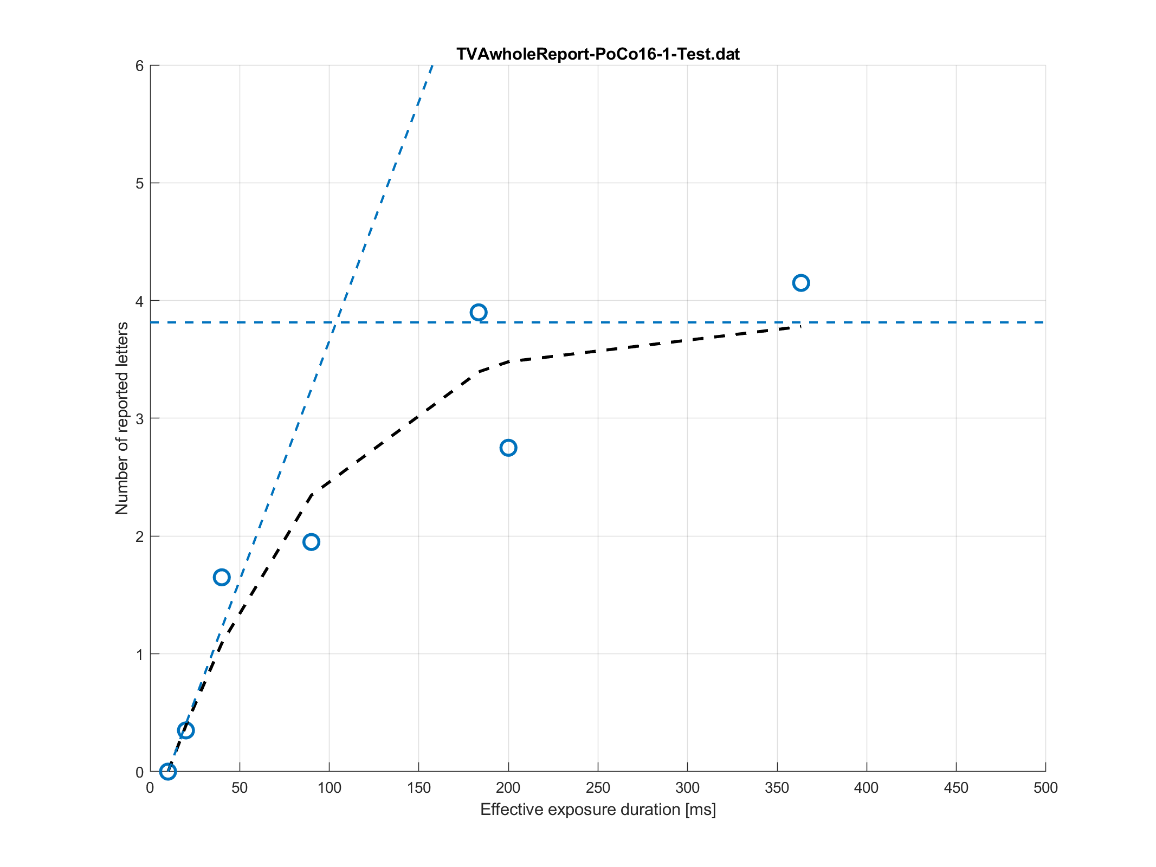


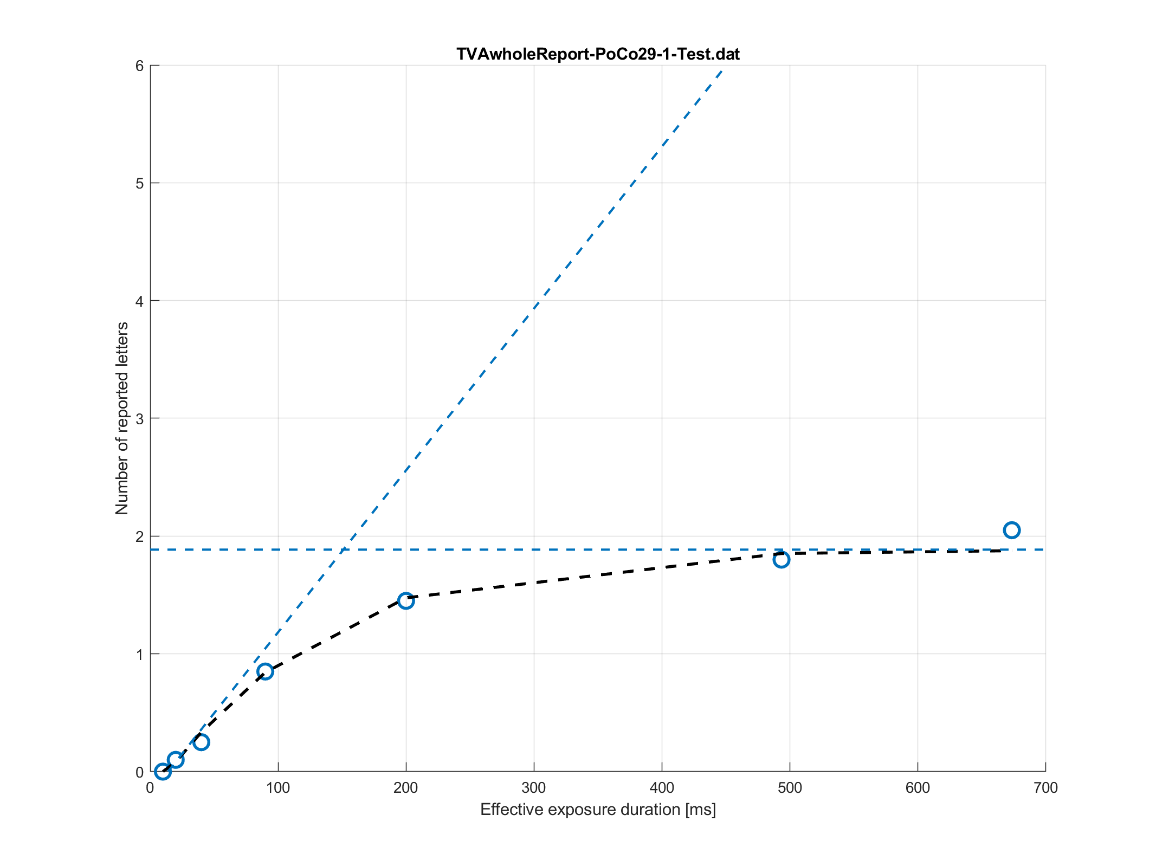

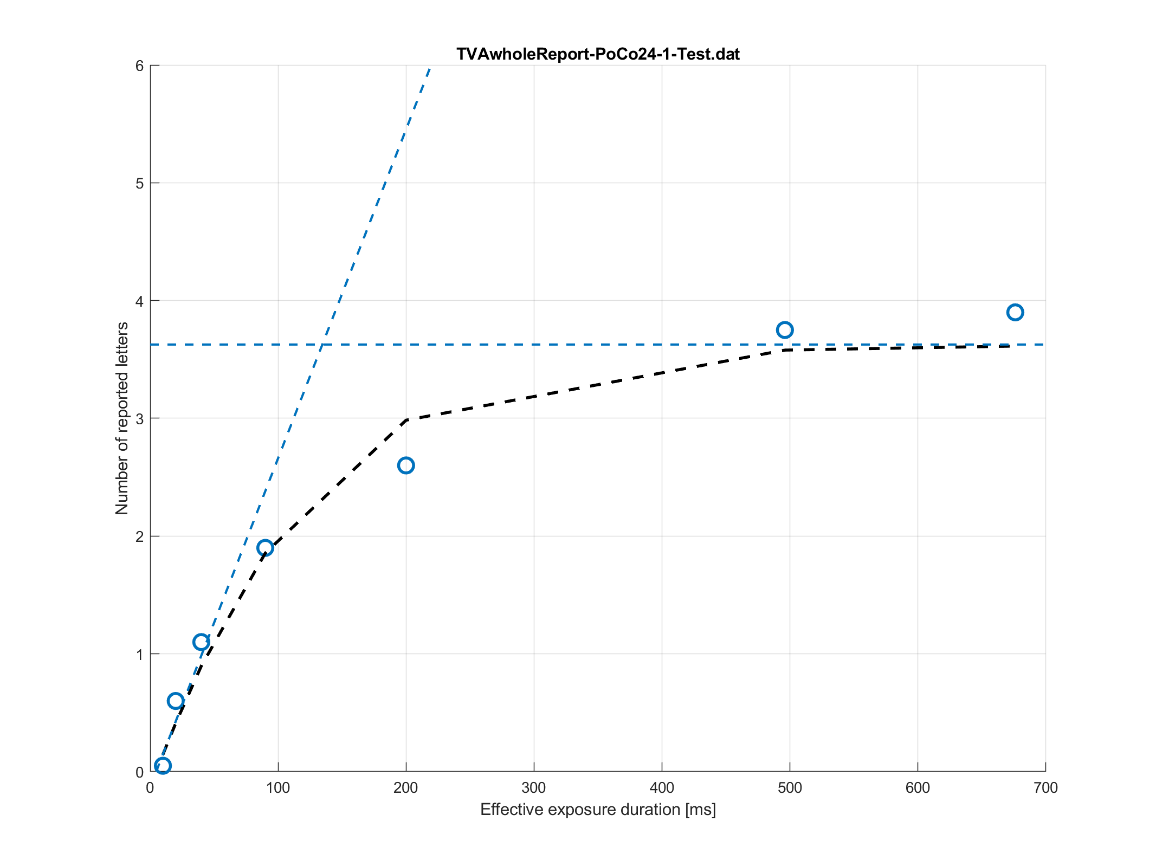

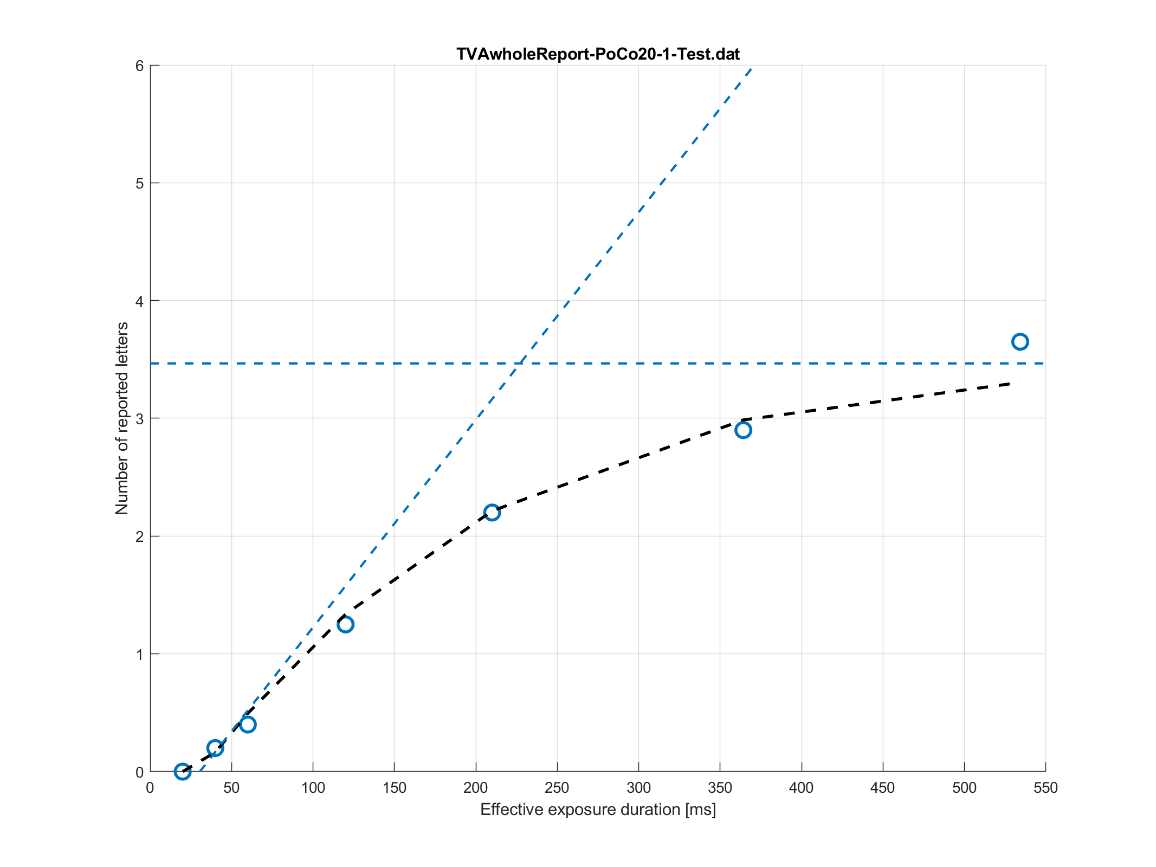

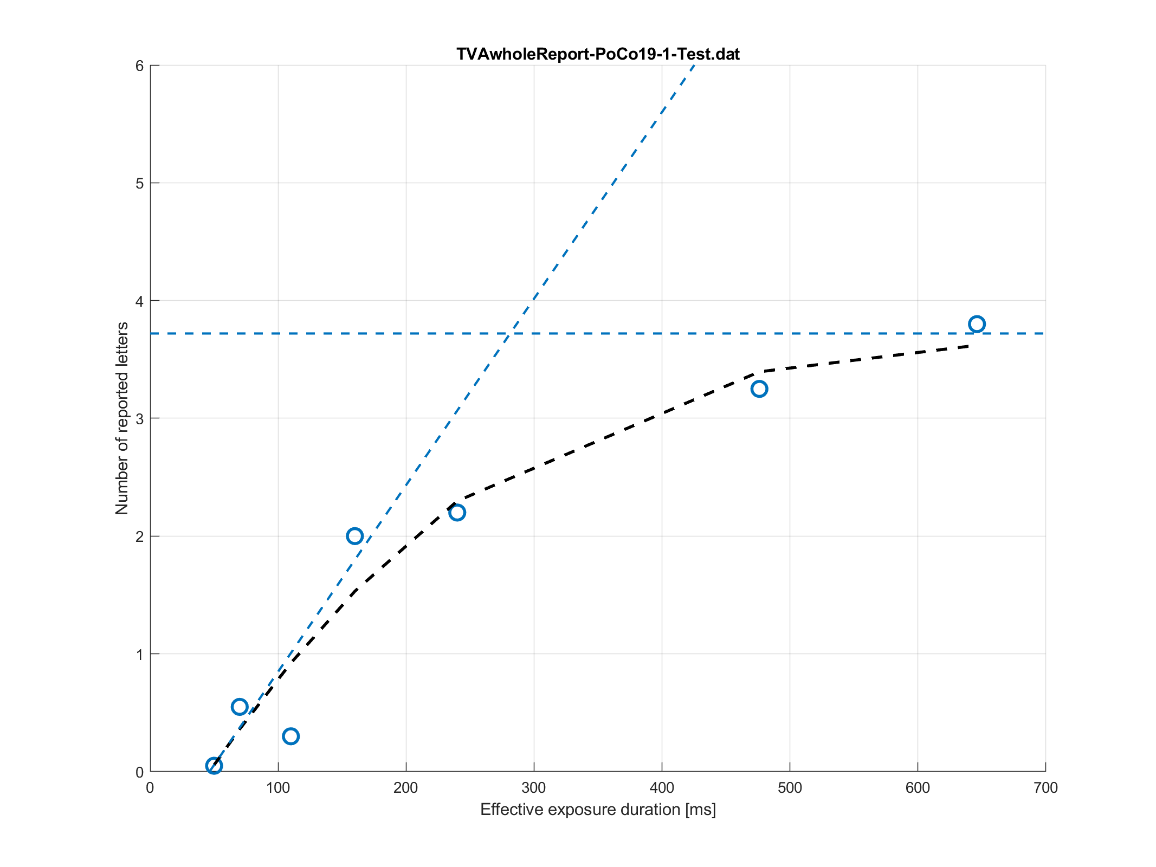

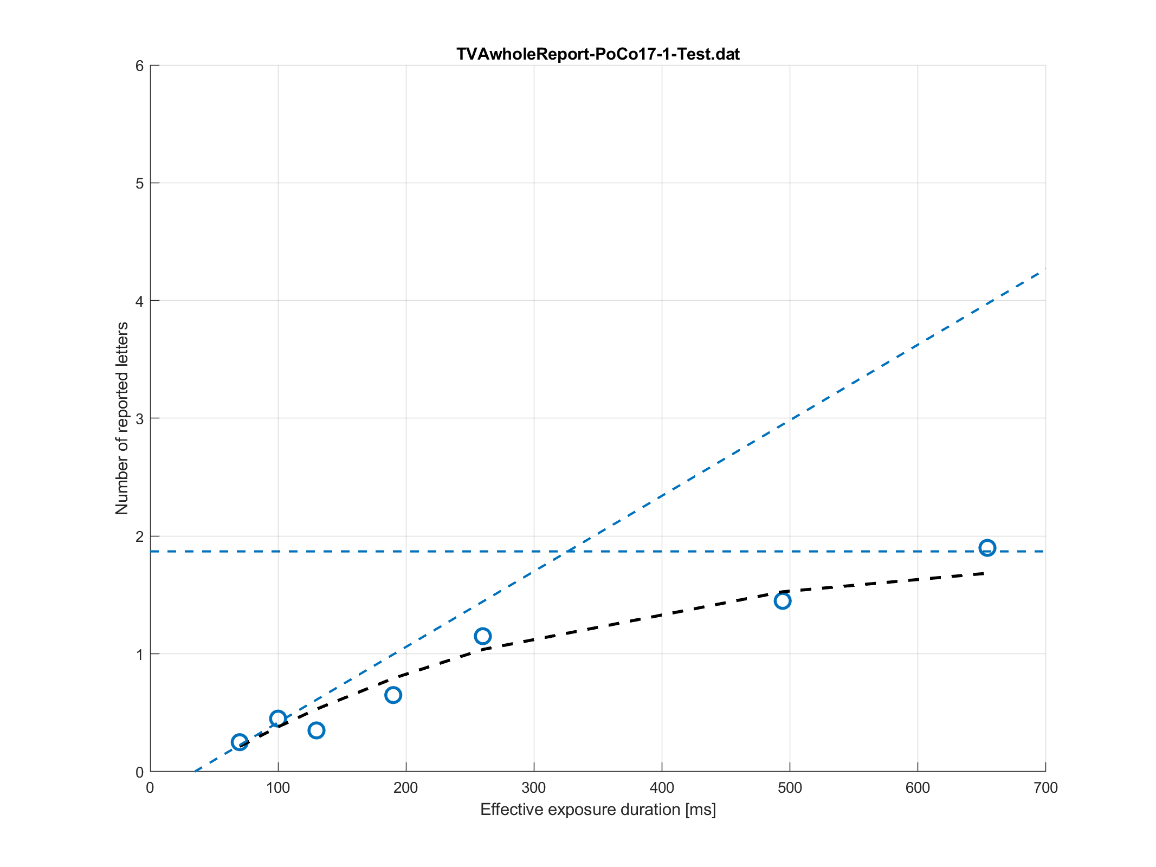

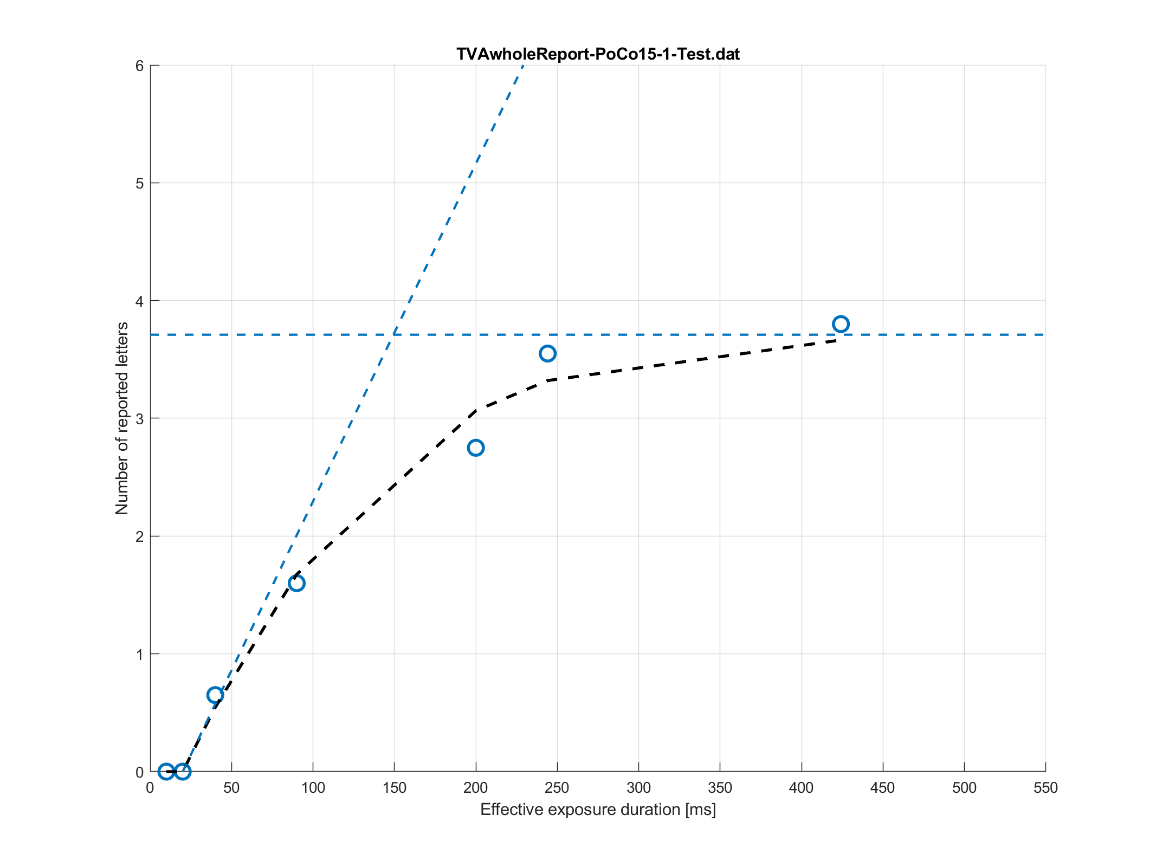

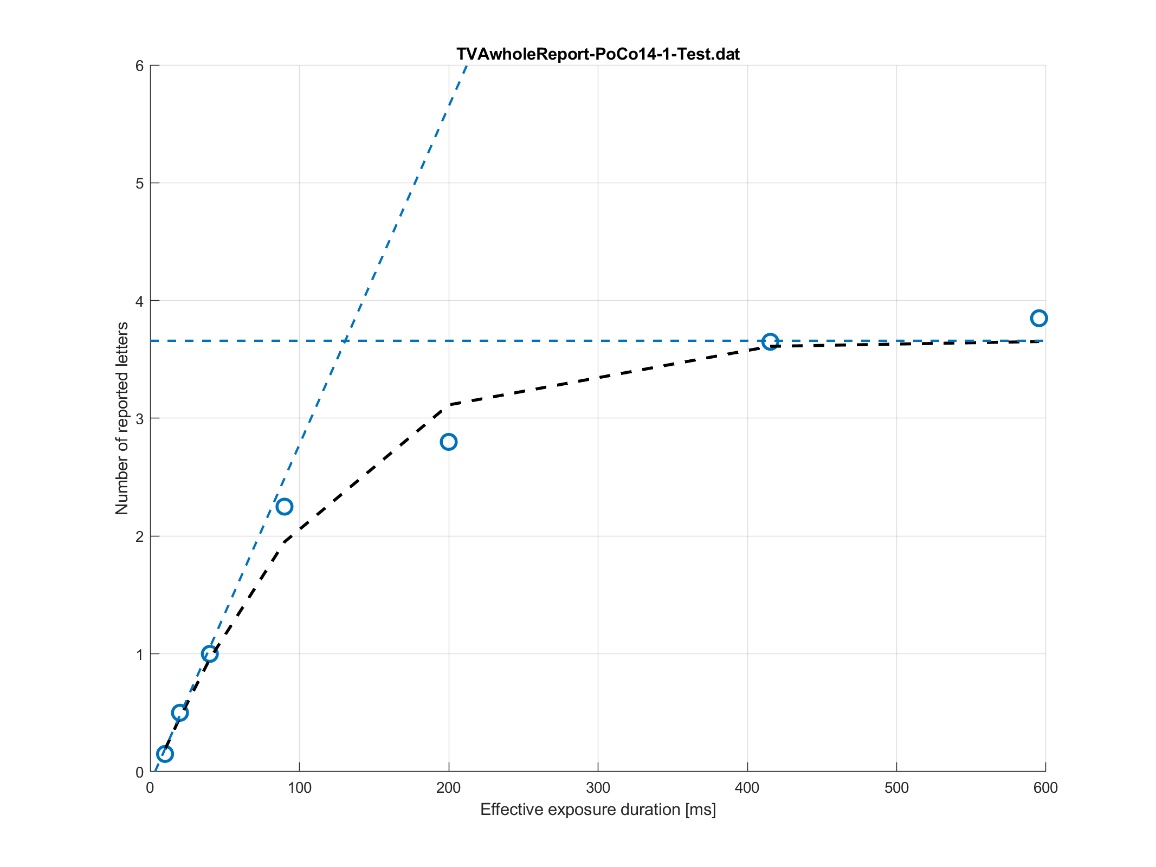

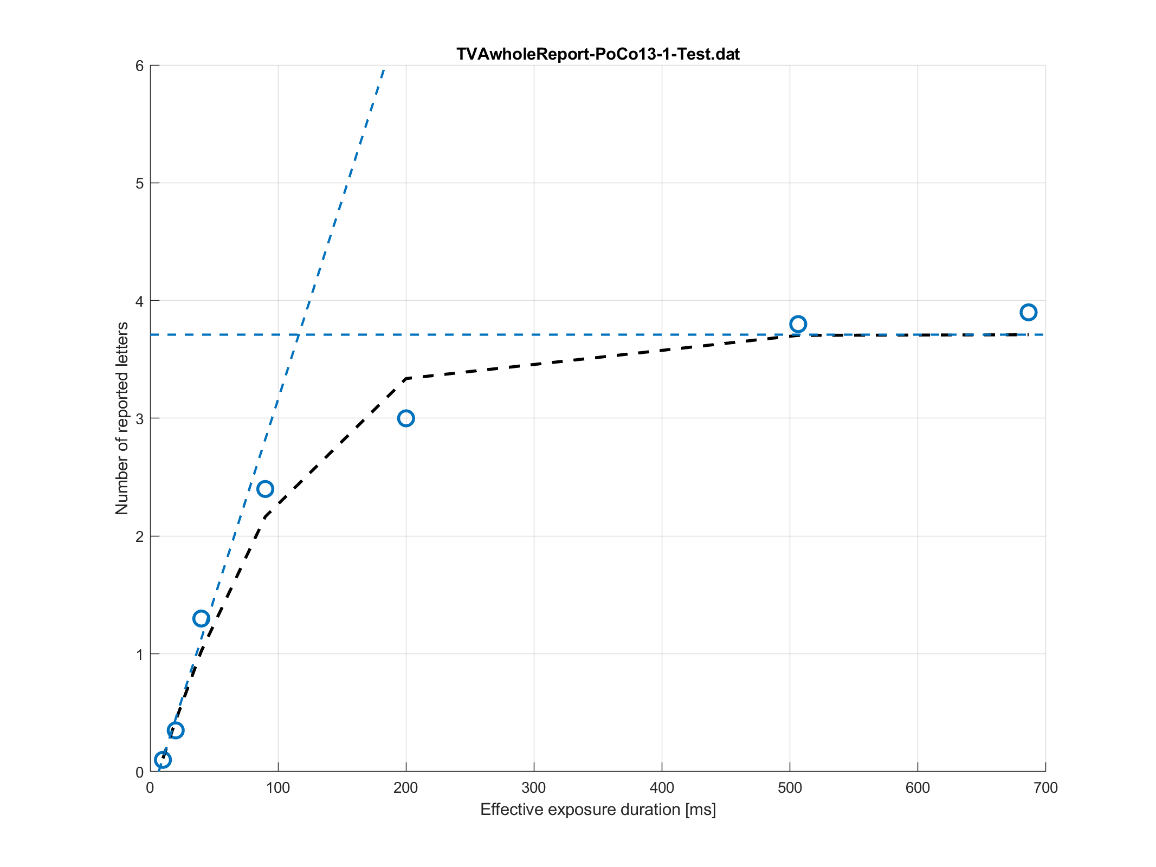

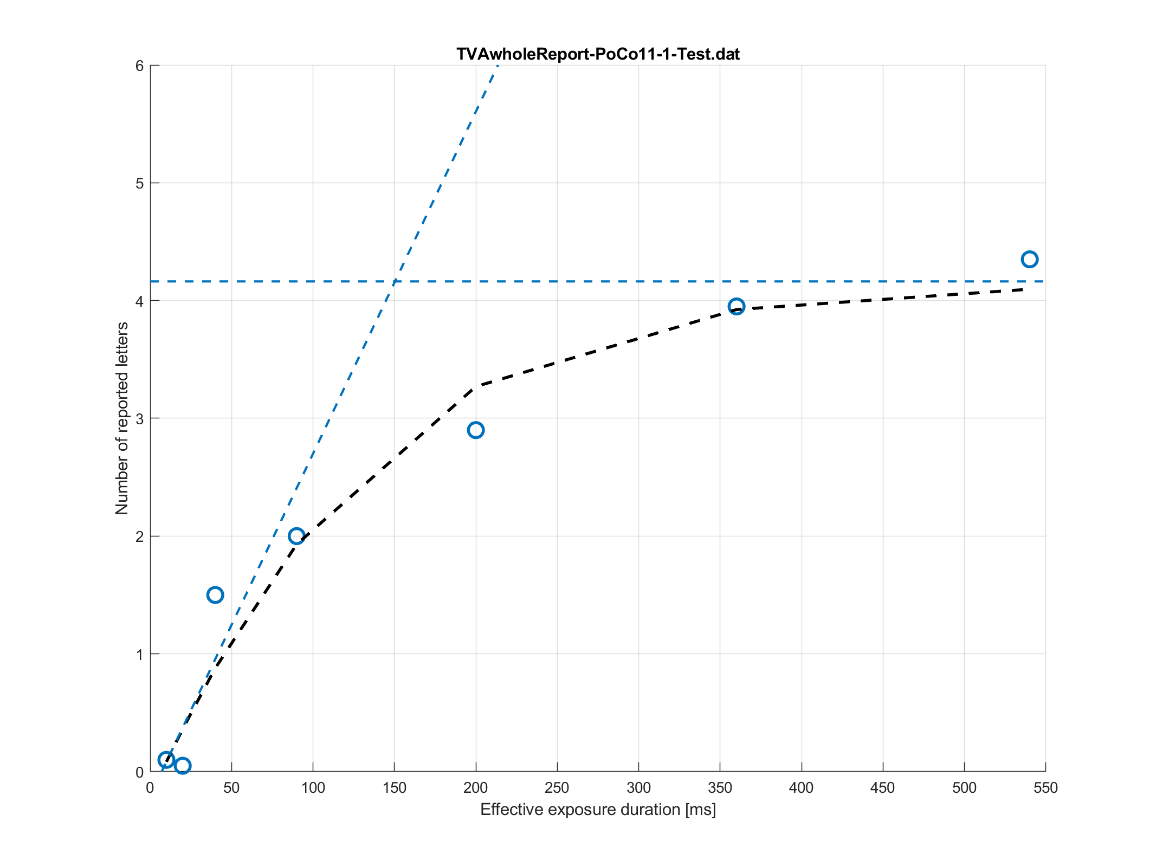

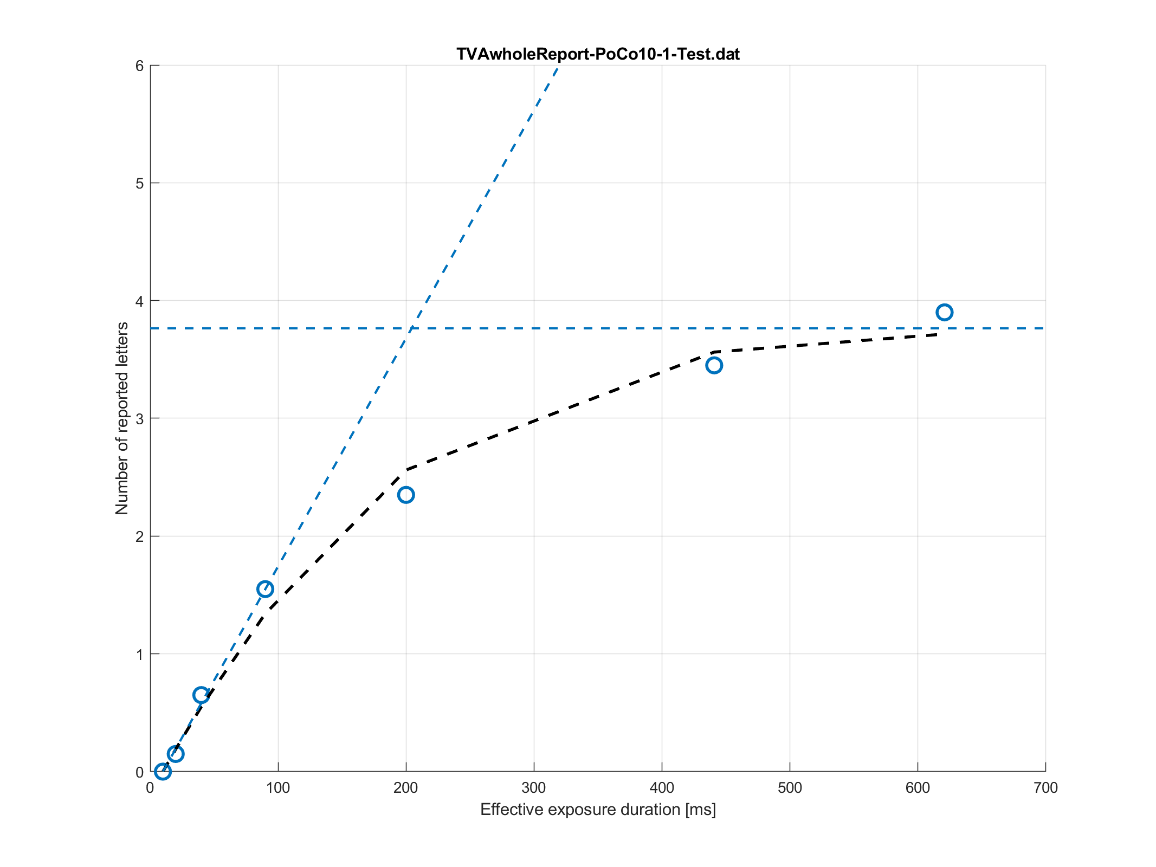

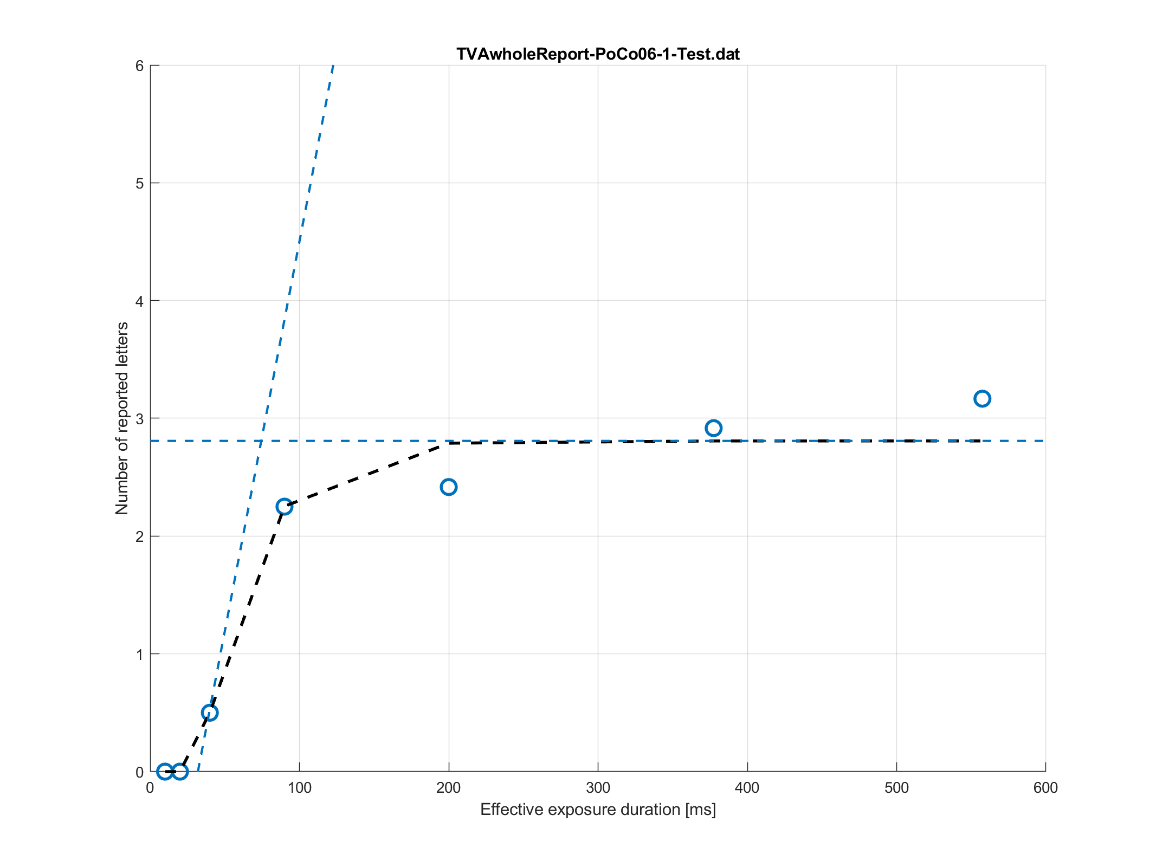

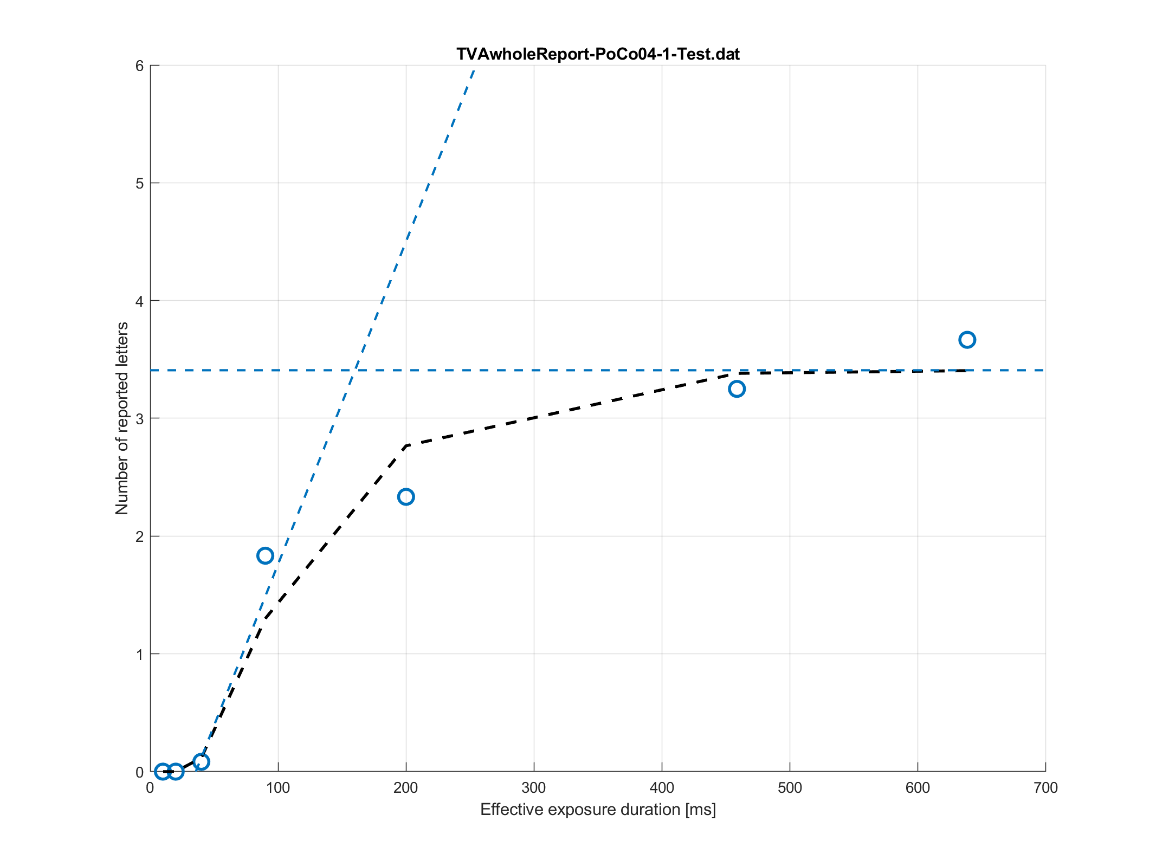

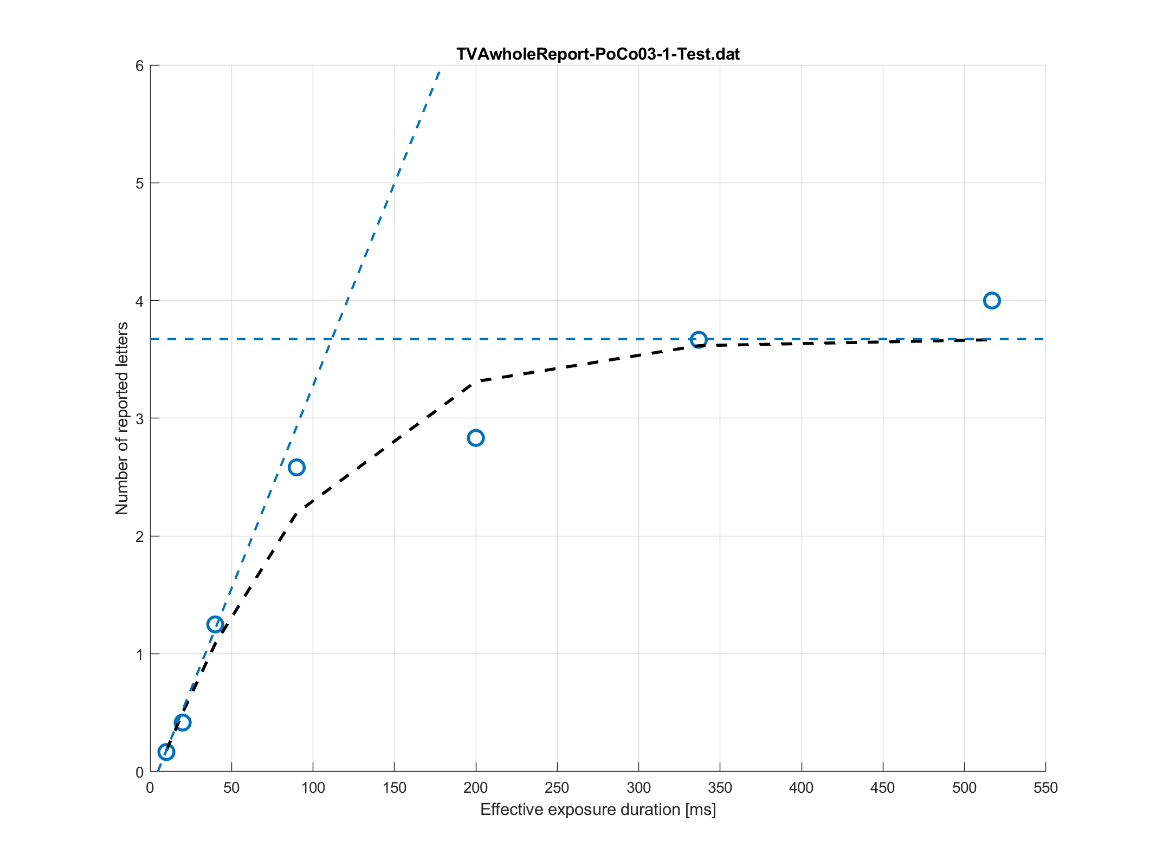

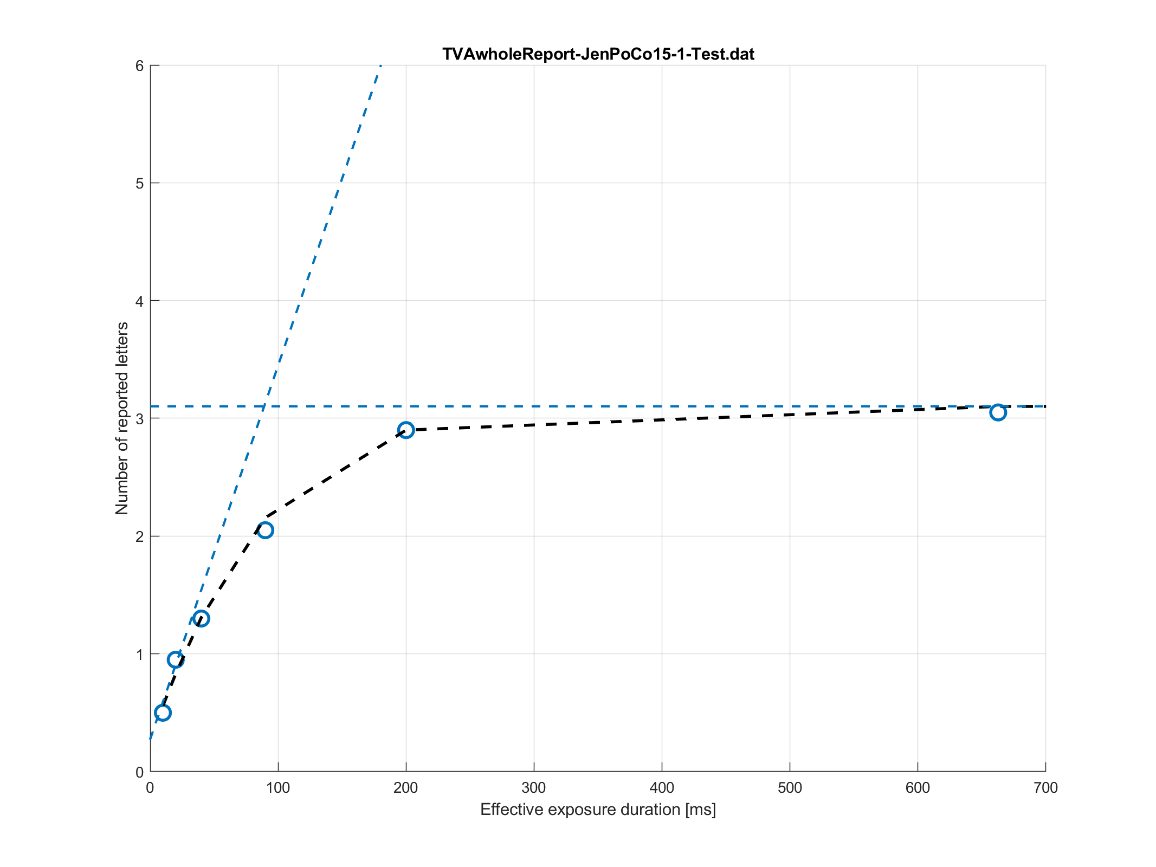

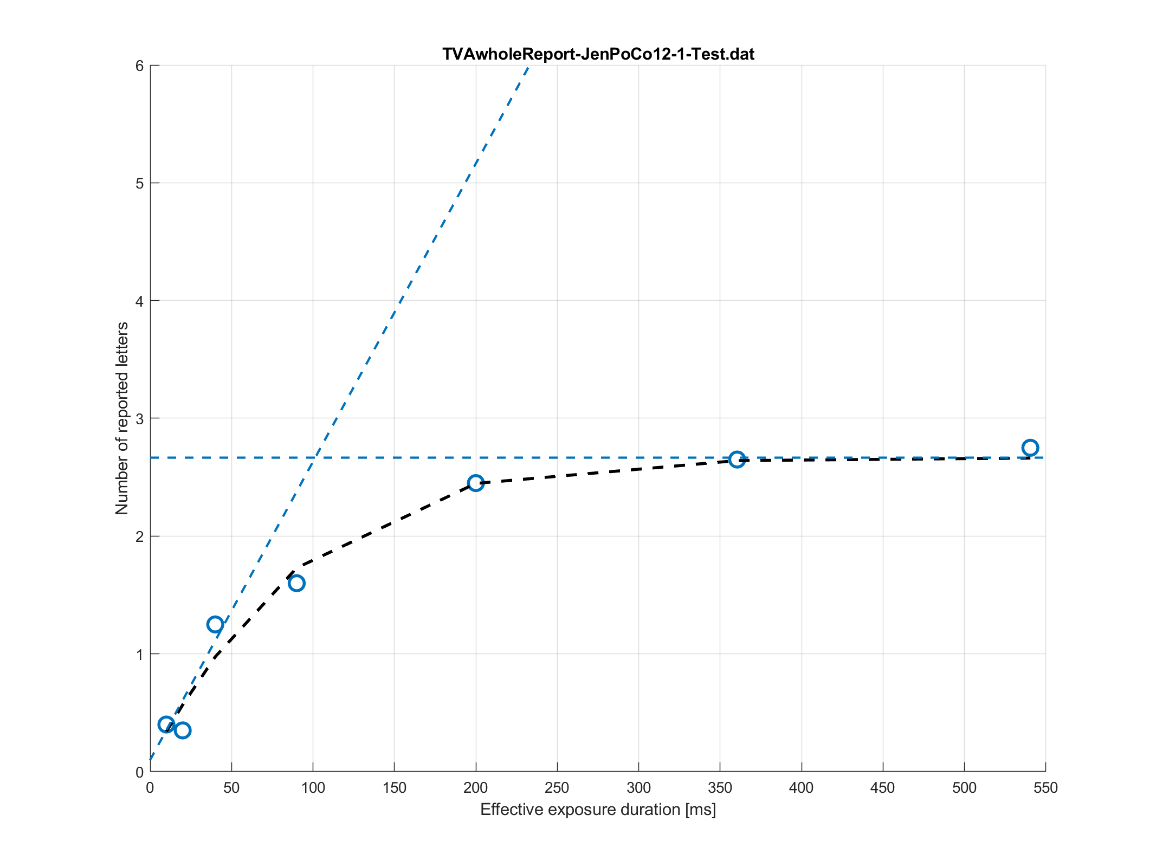

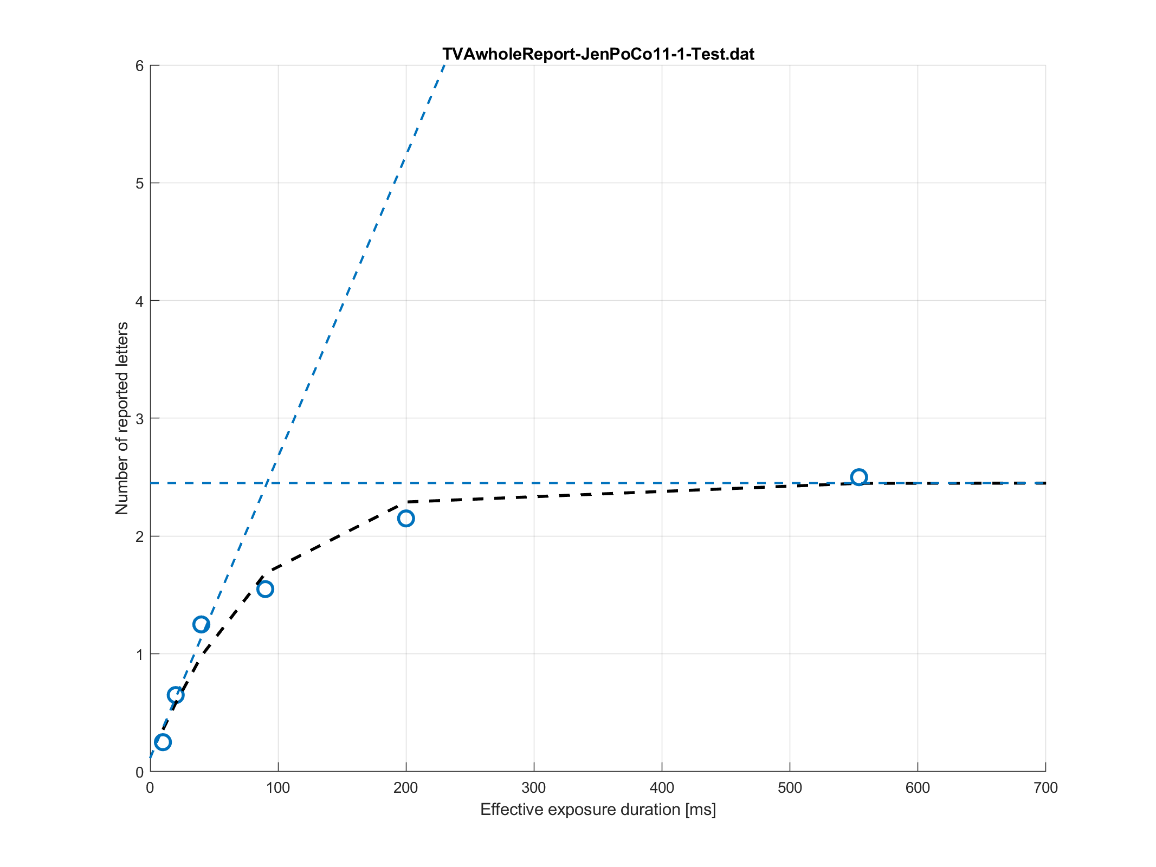

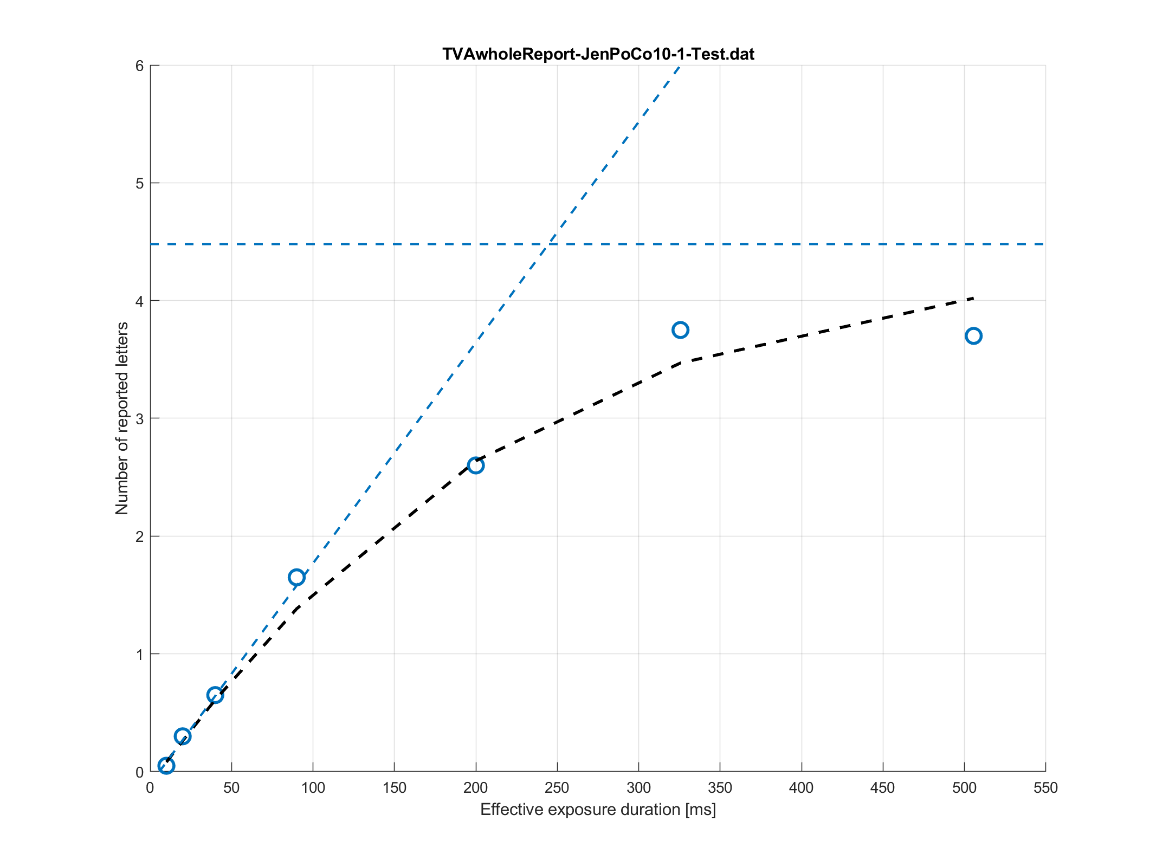

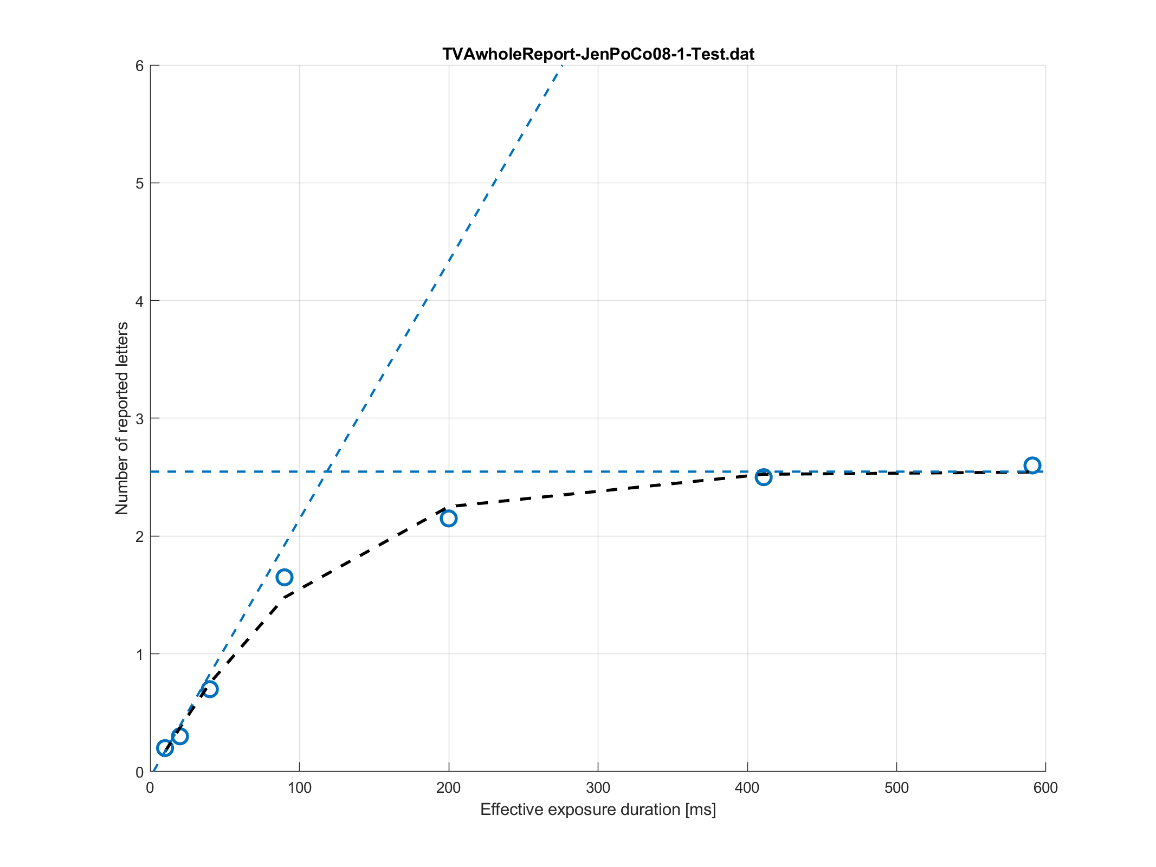

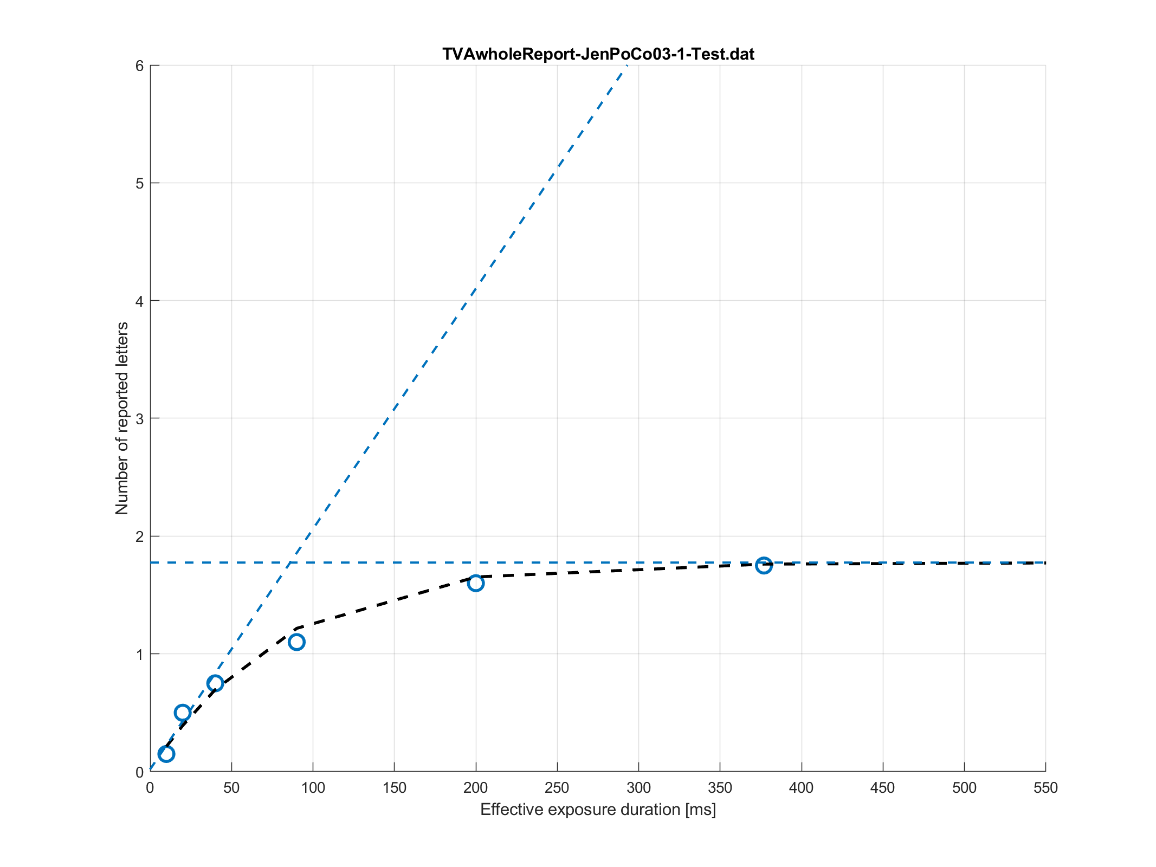

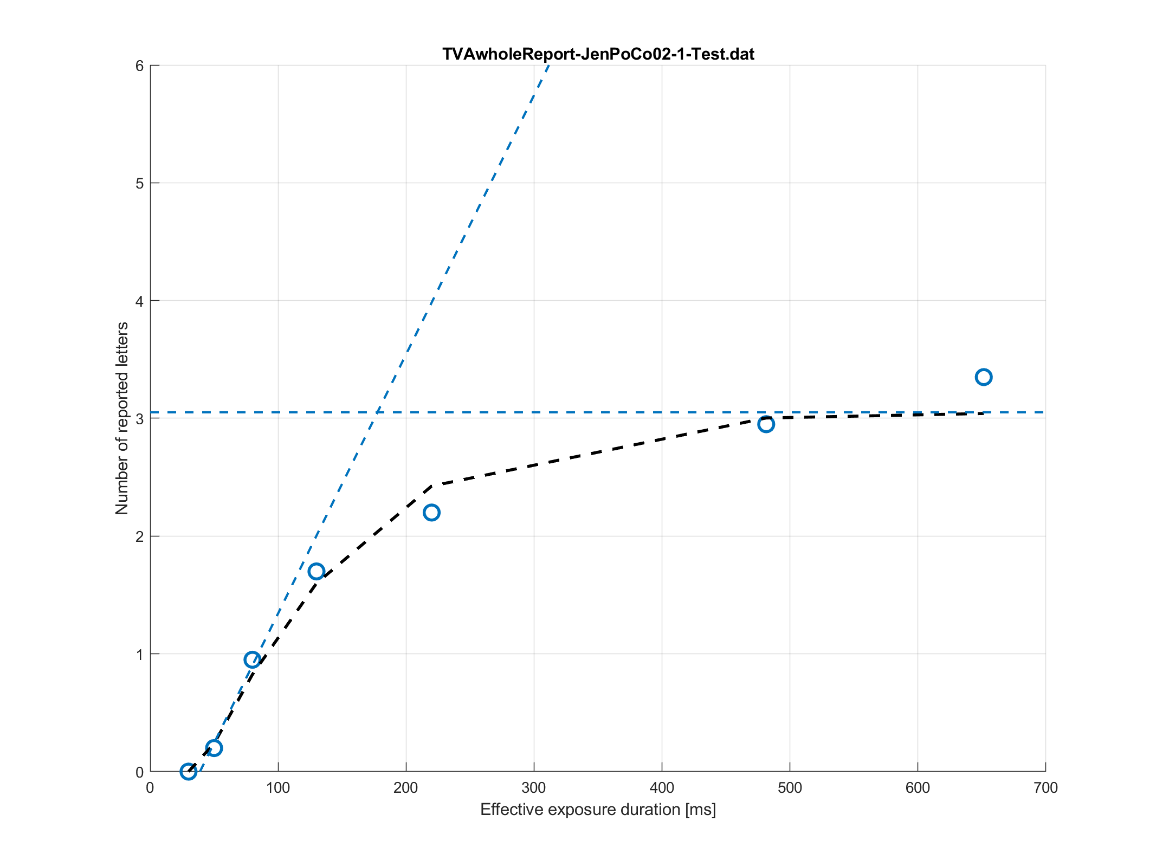

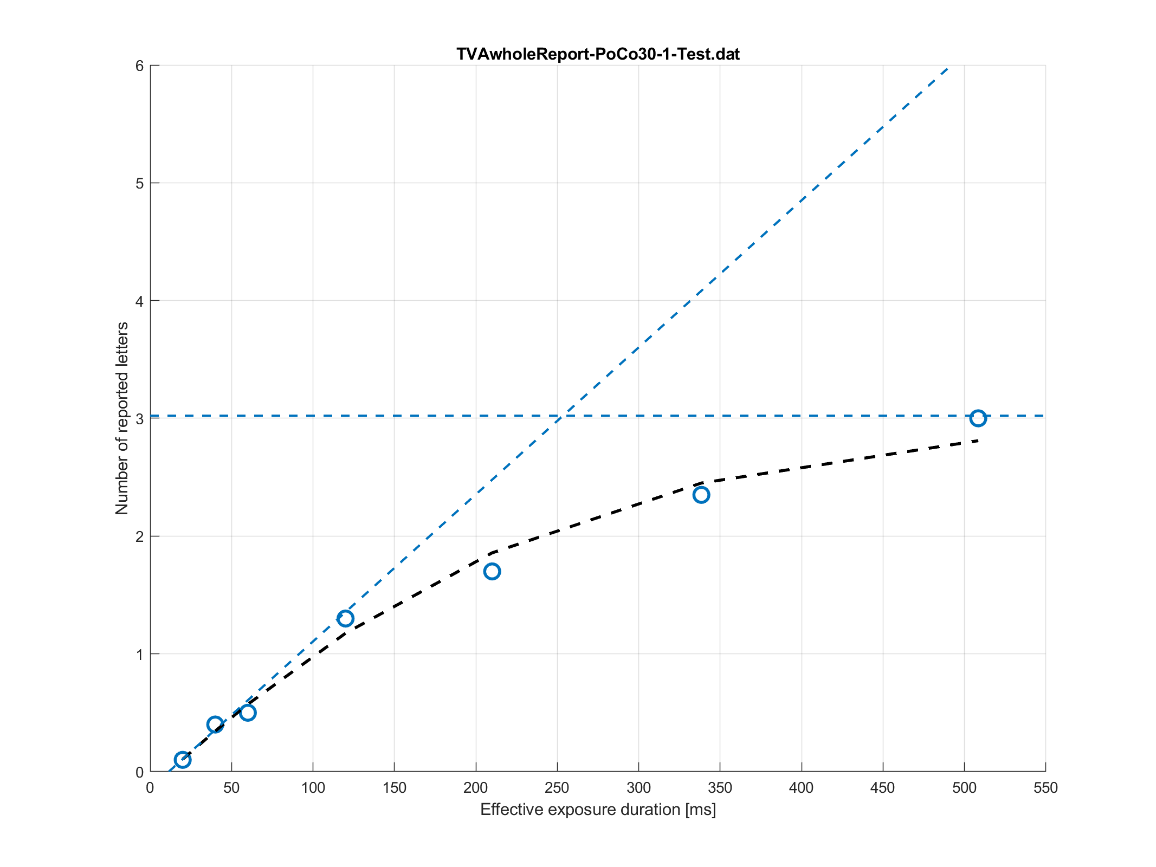

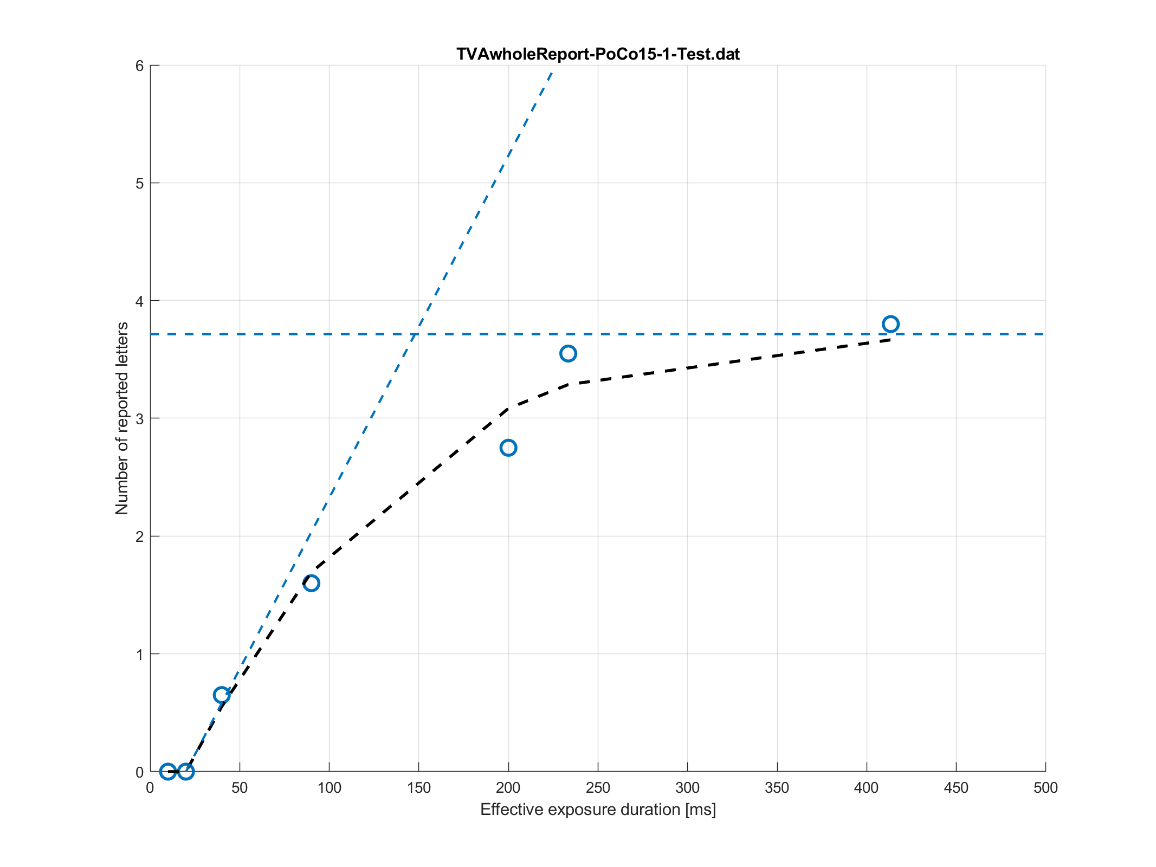

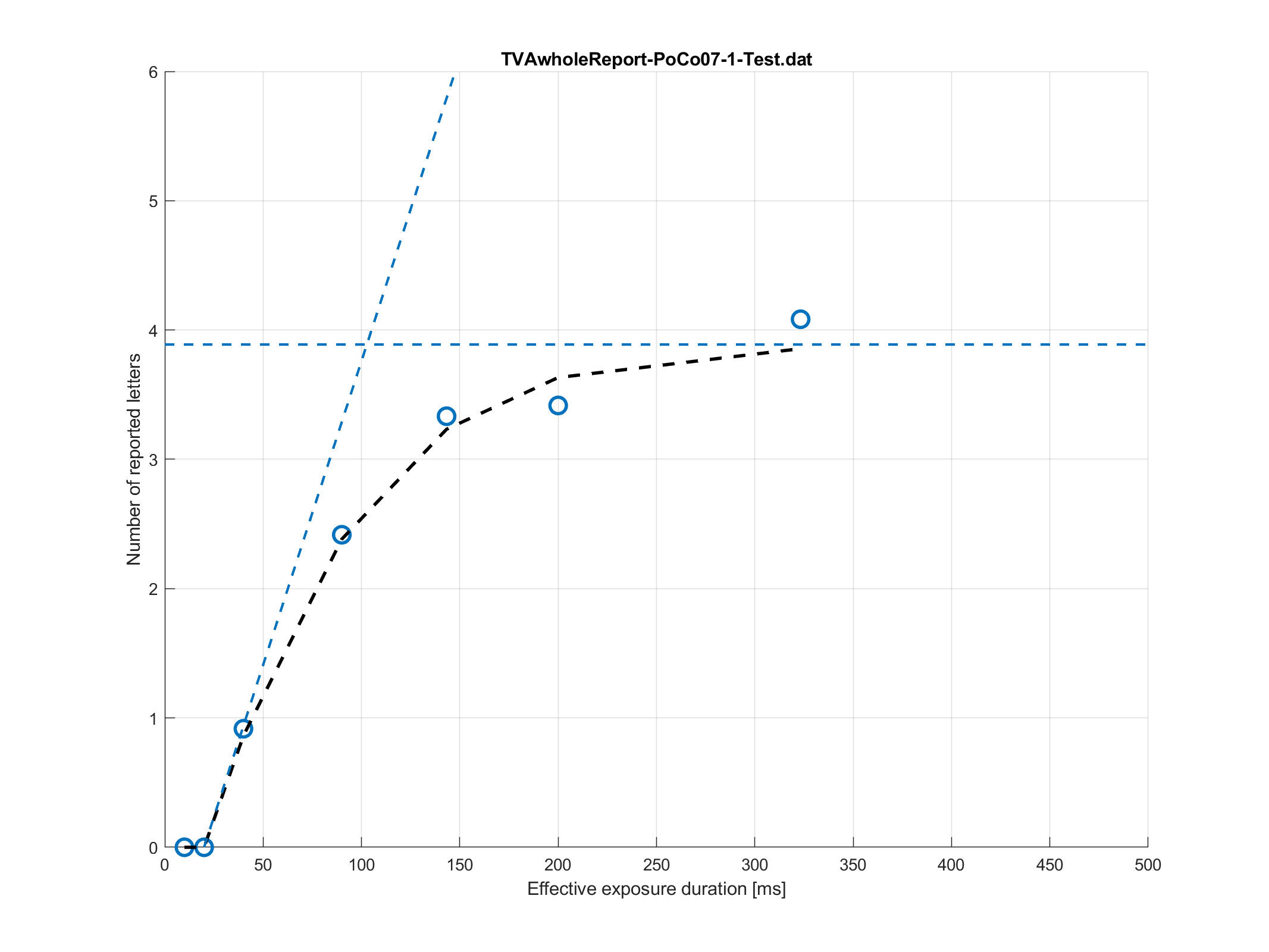

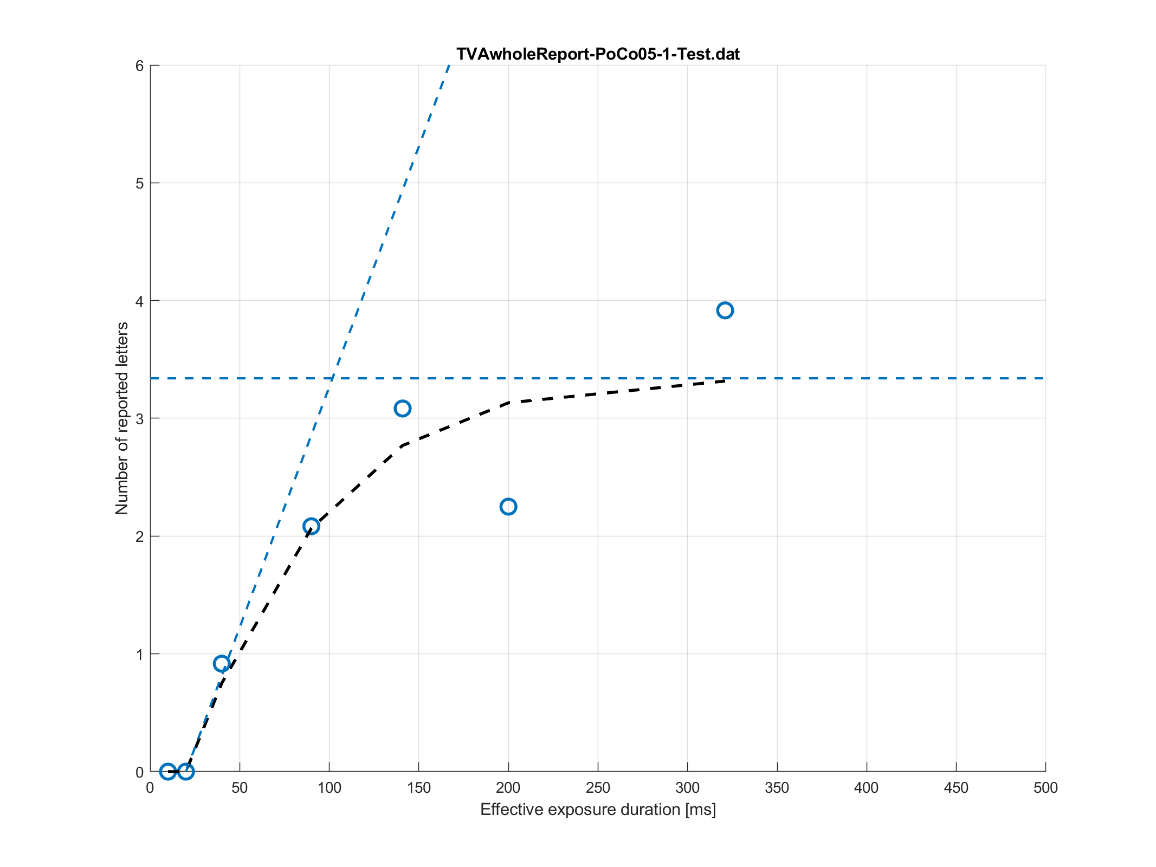

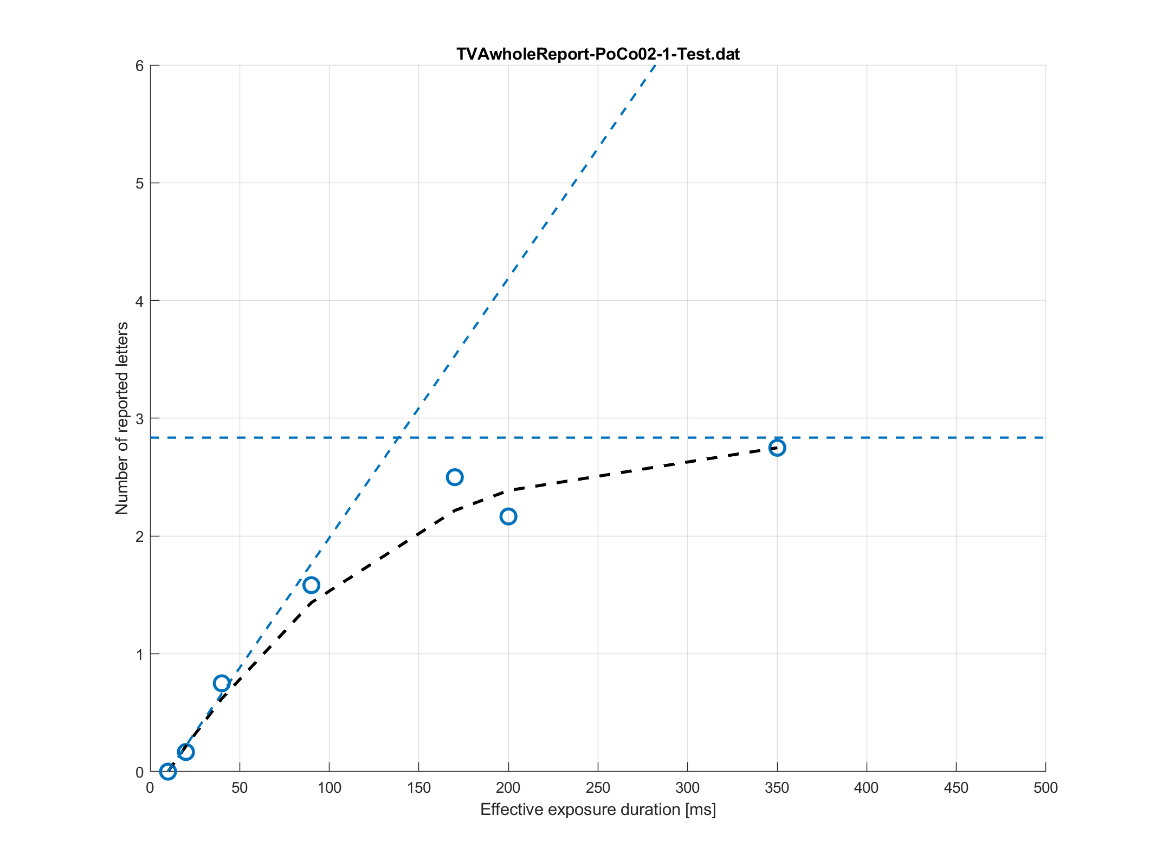

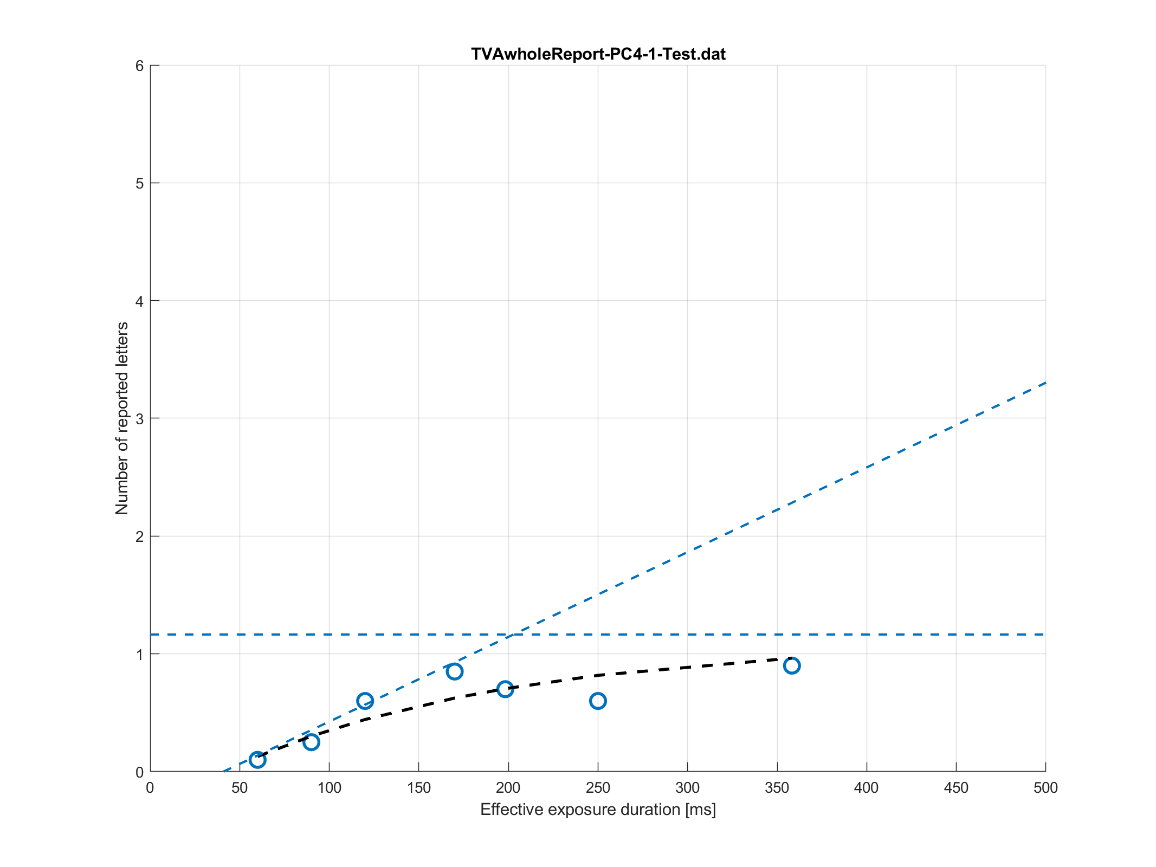

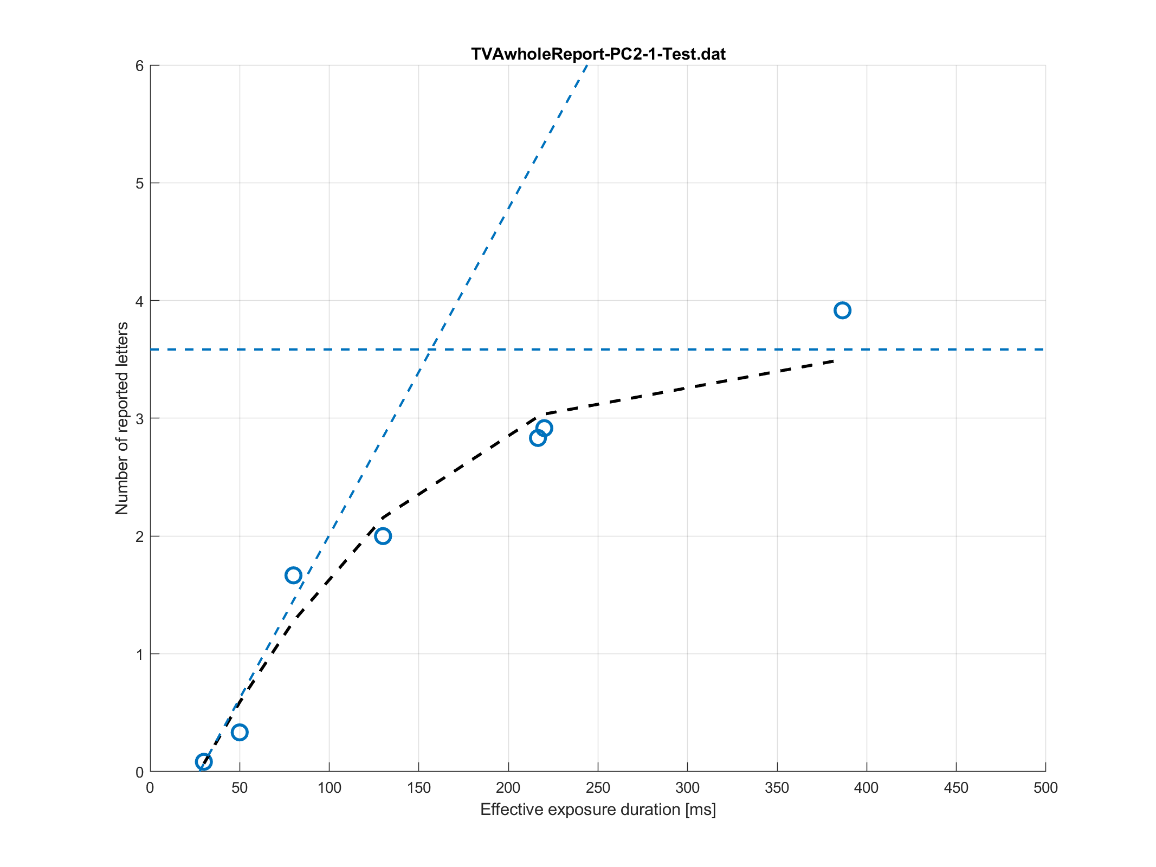

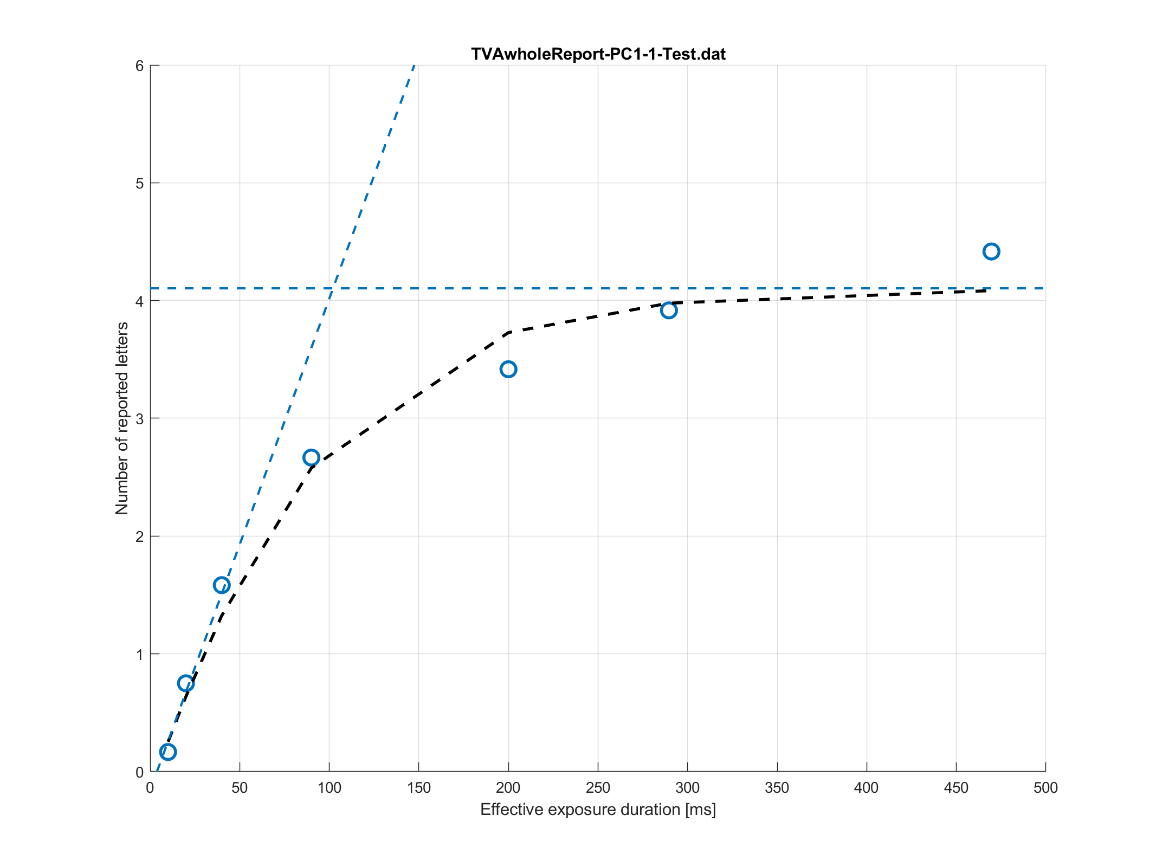

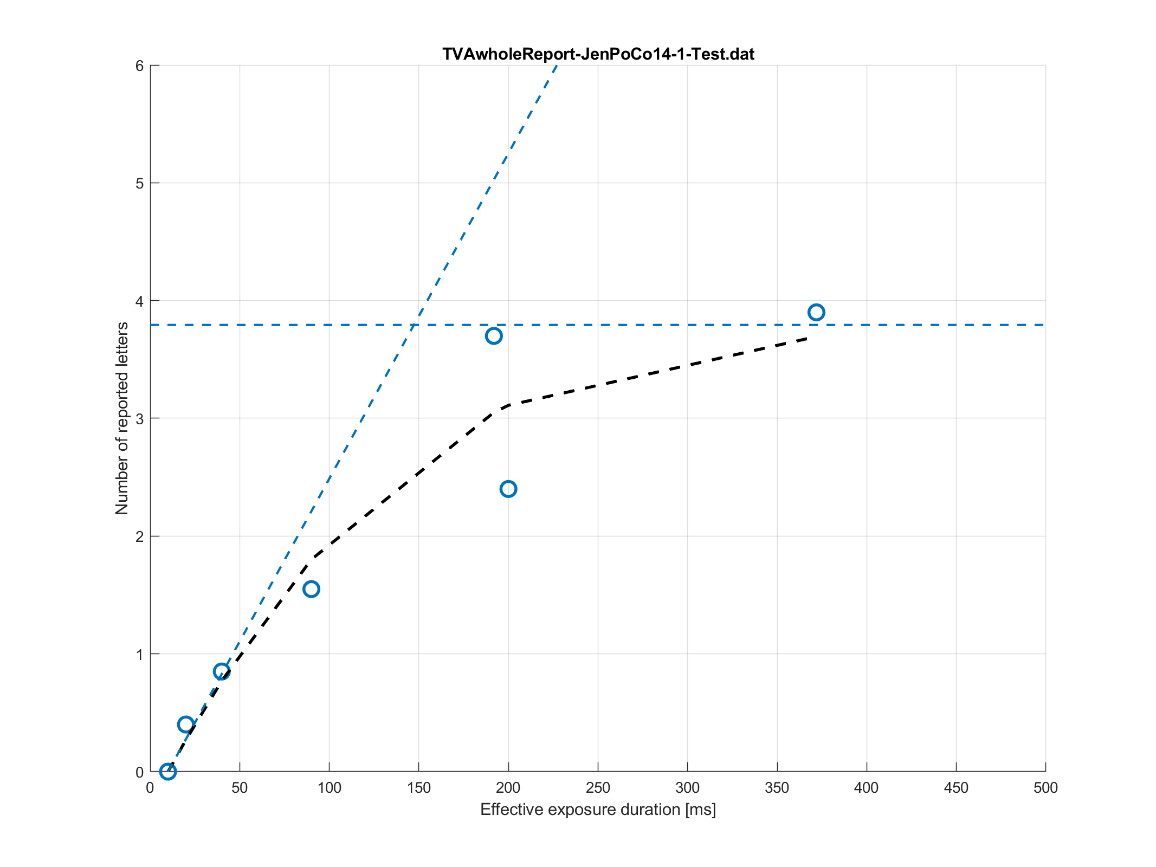

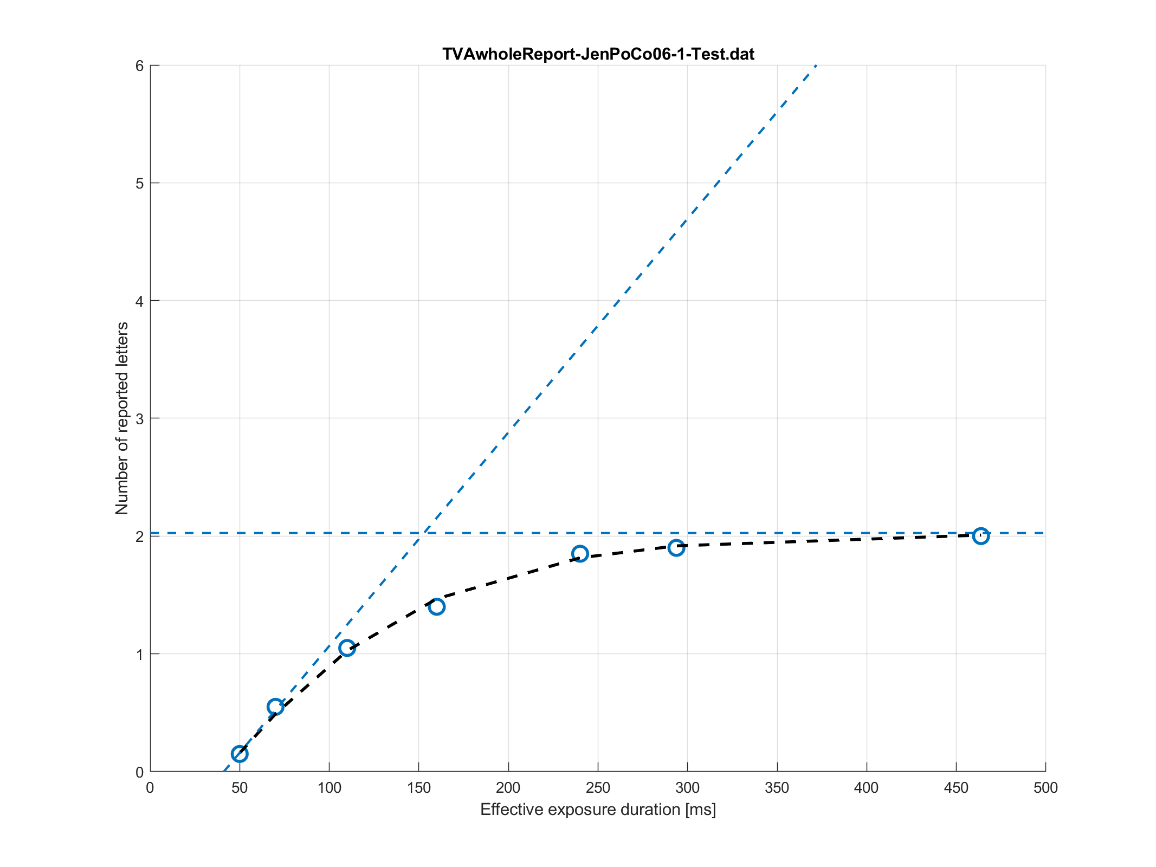

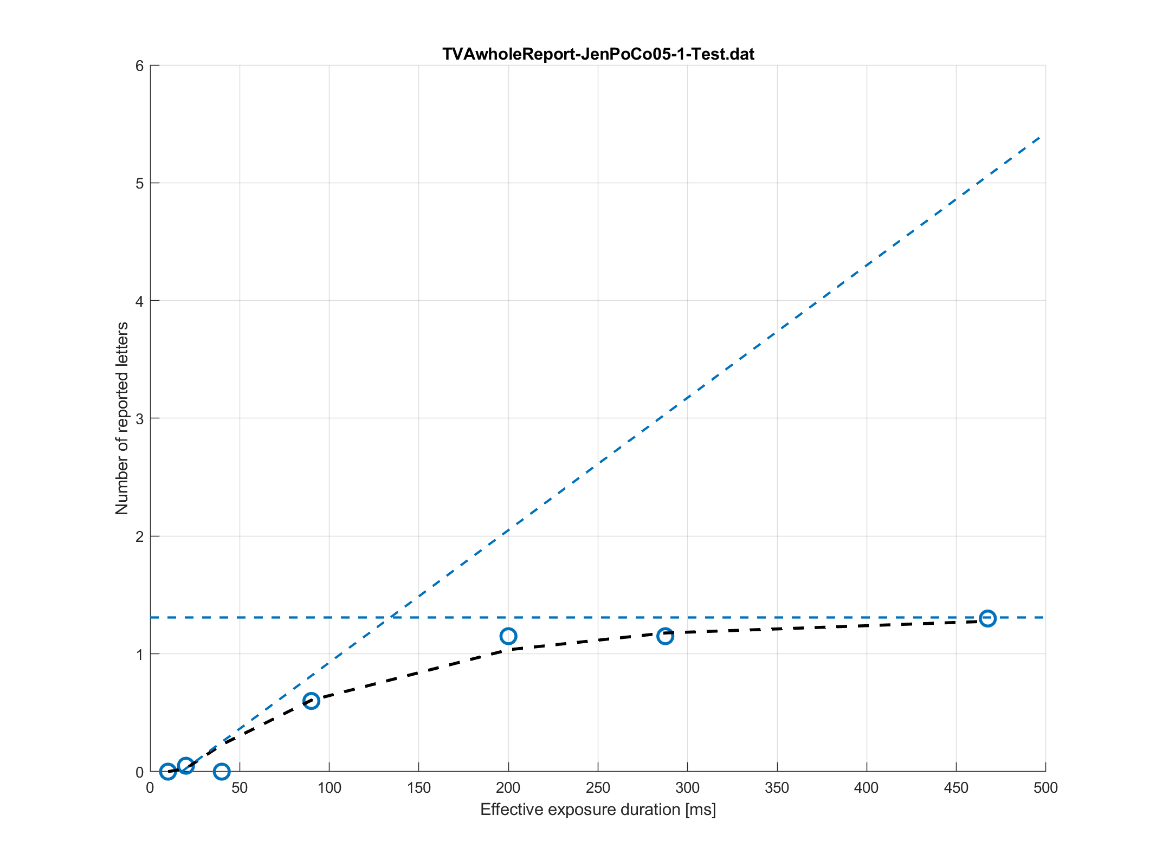

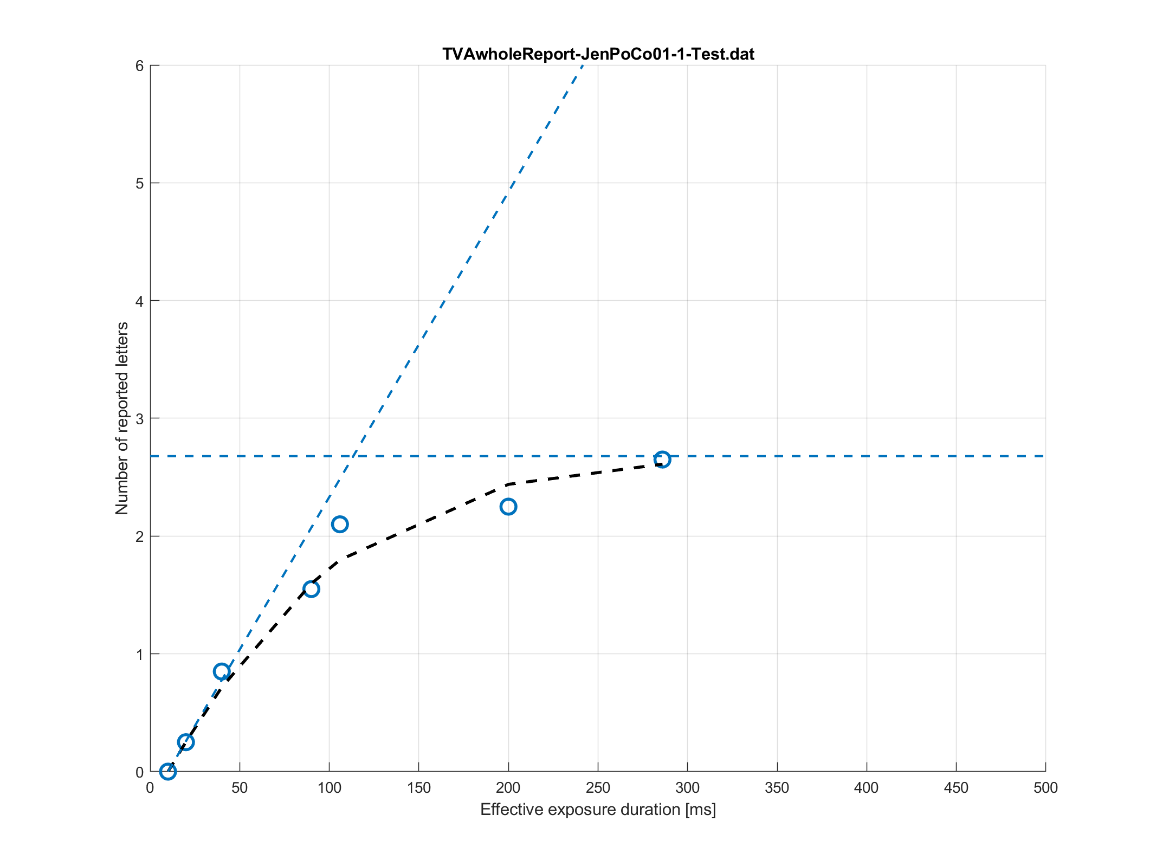


#### Fitted whole report performance healthy control participant curves


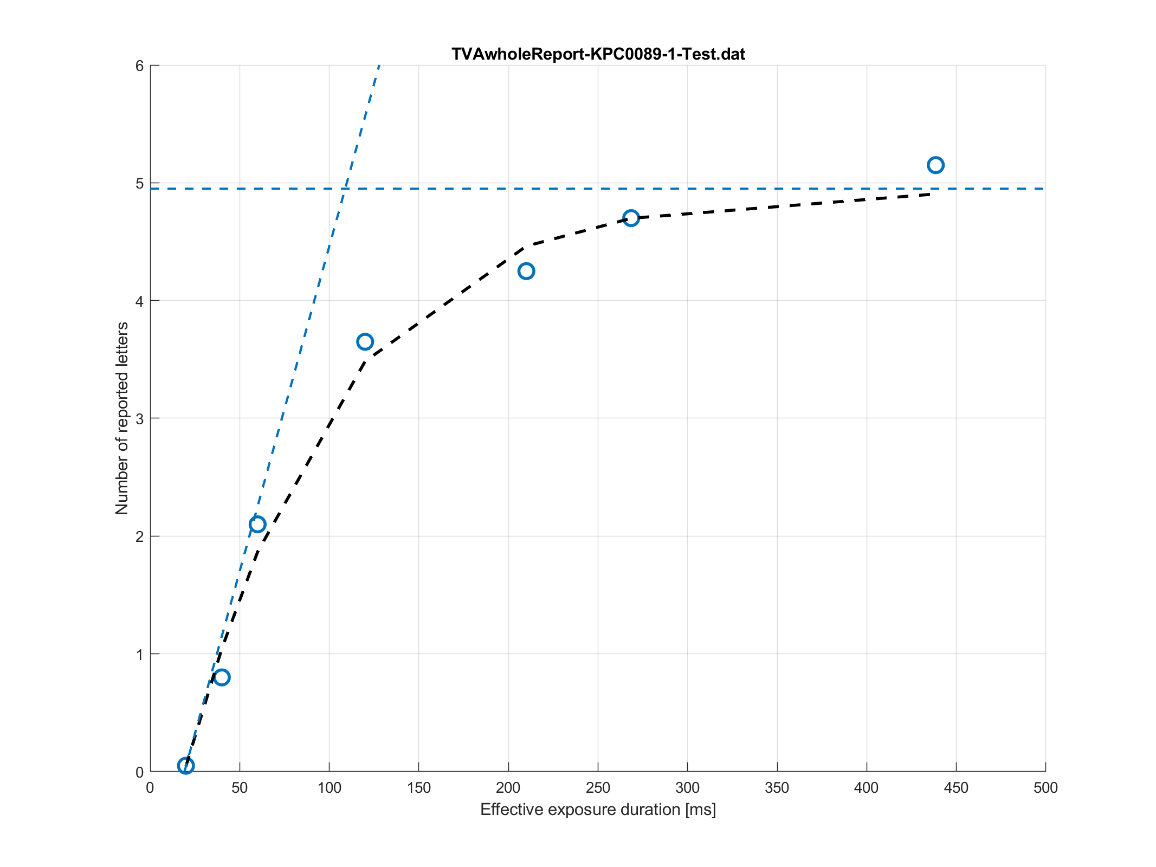

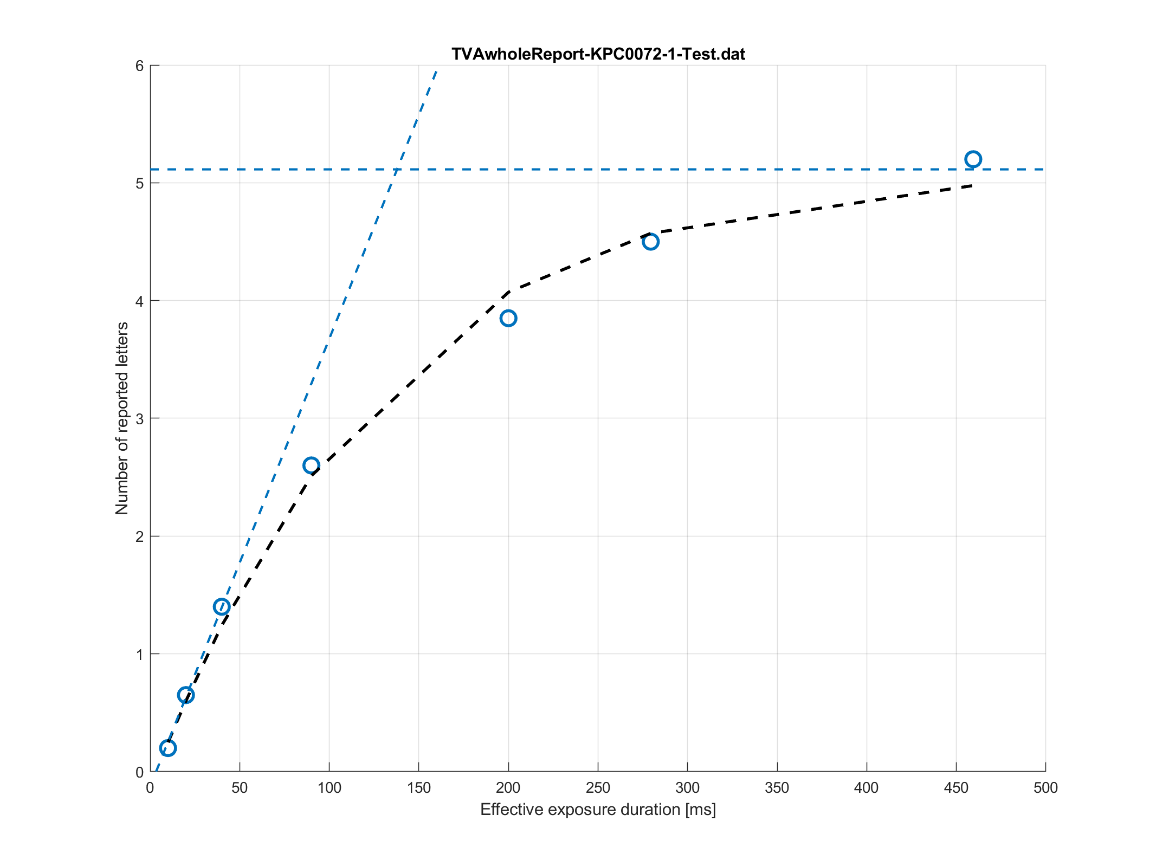

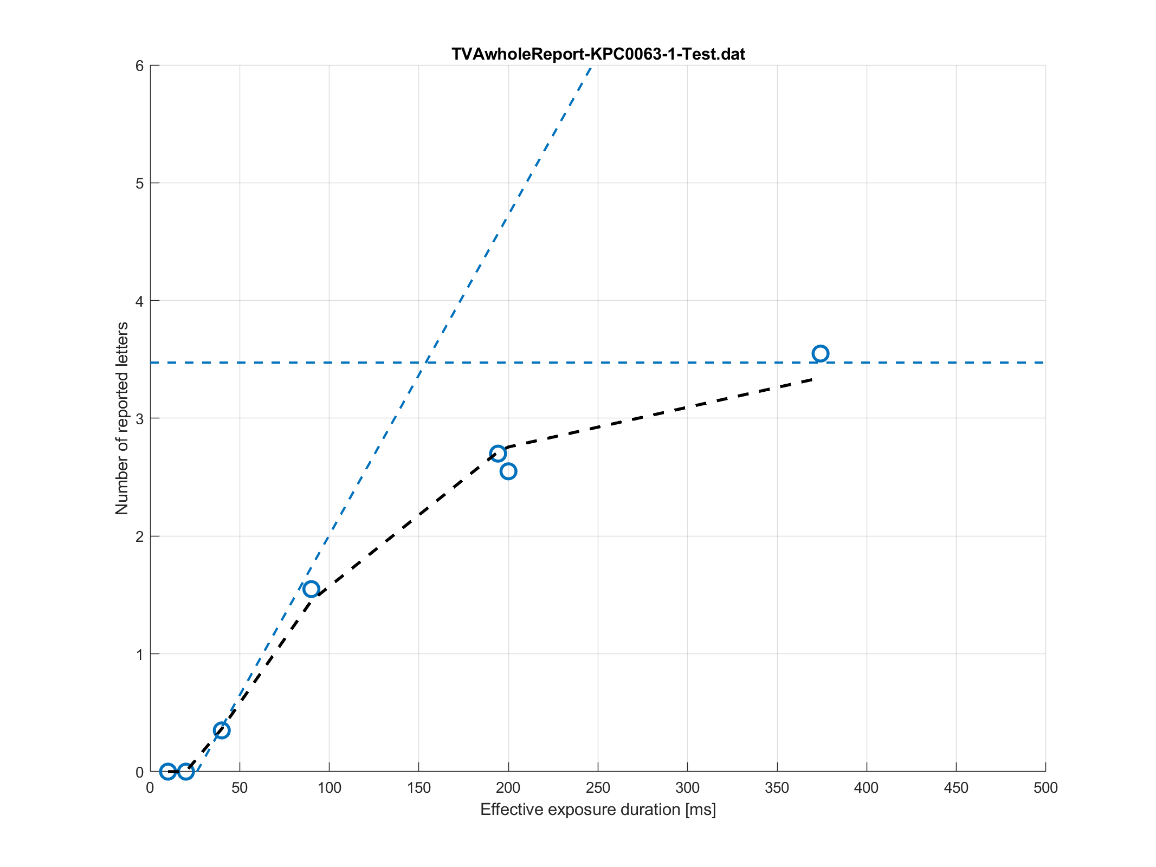


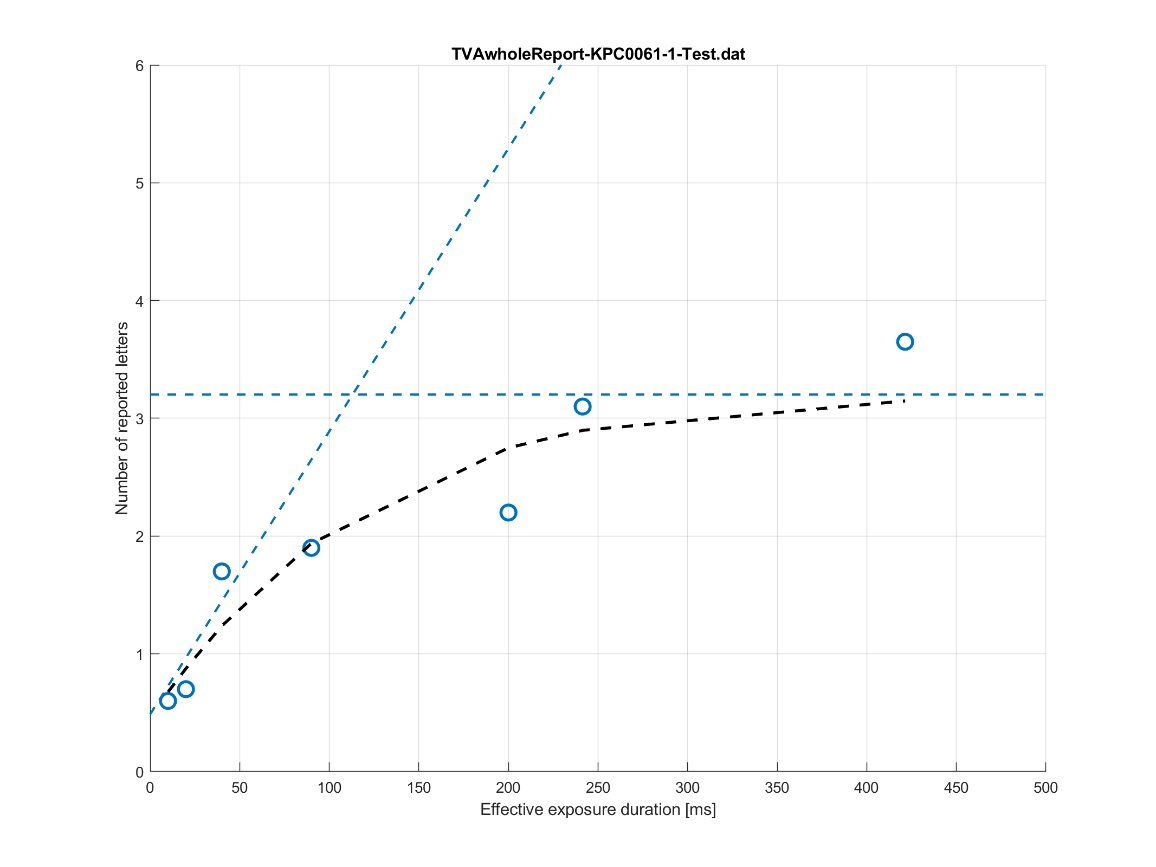

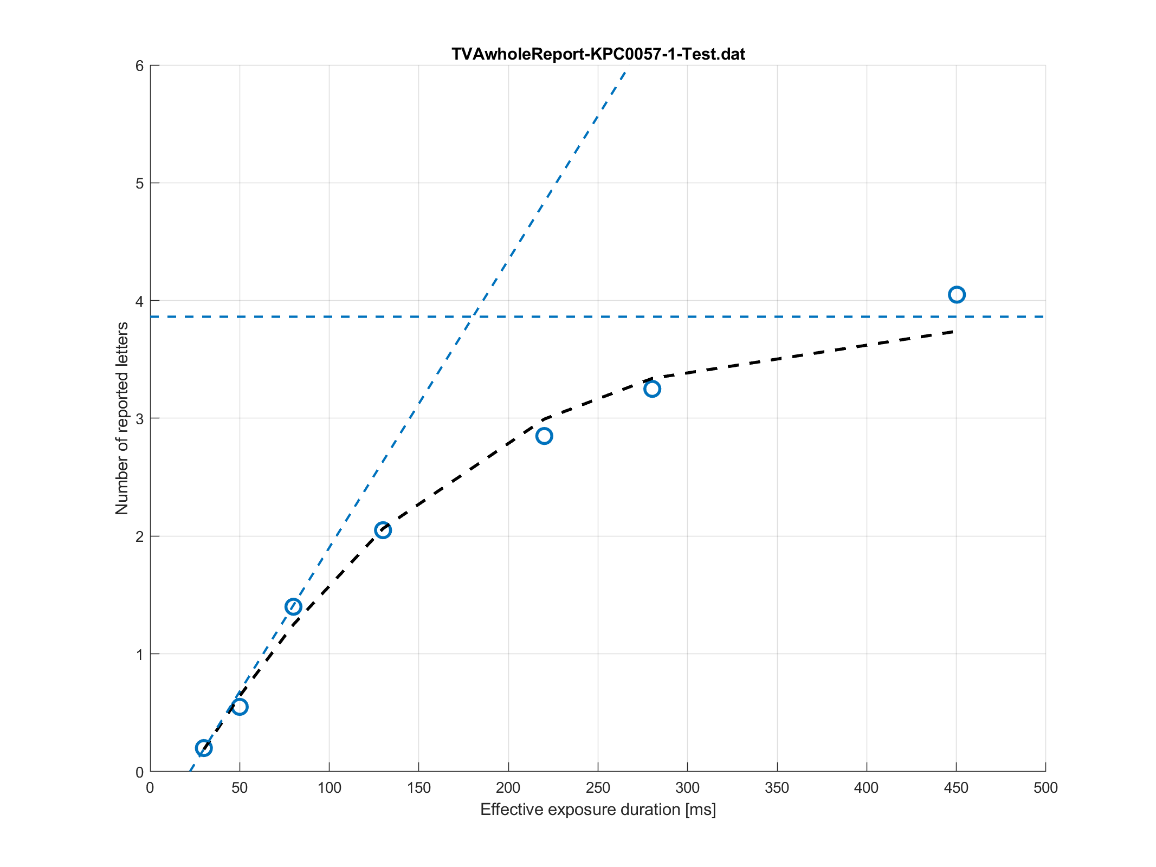


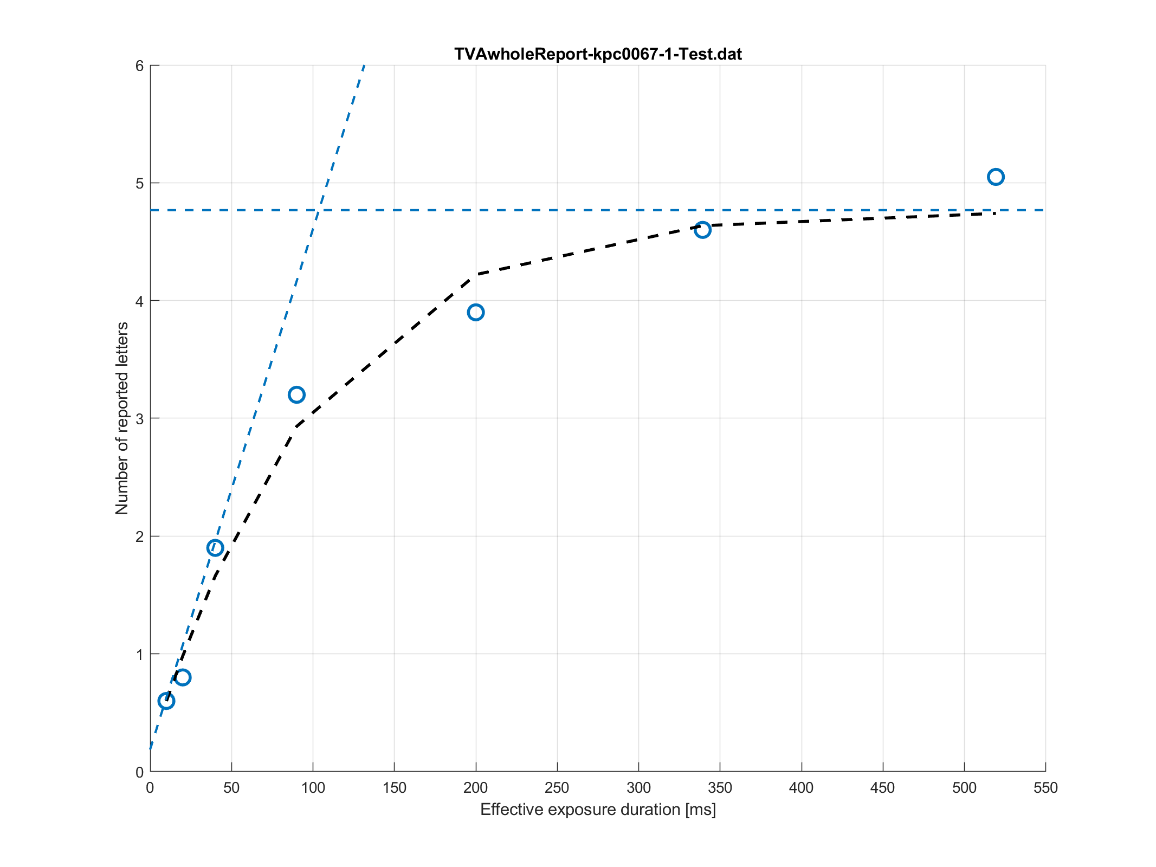

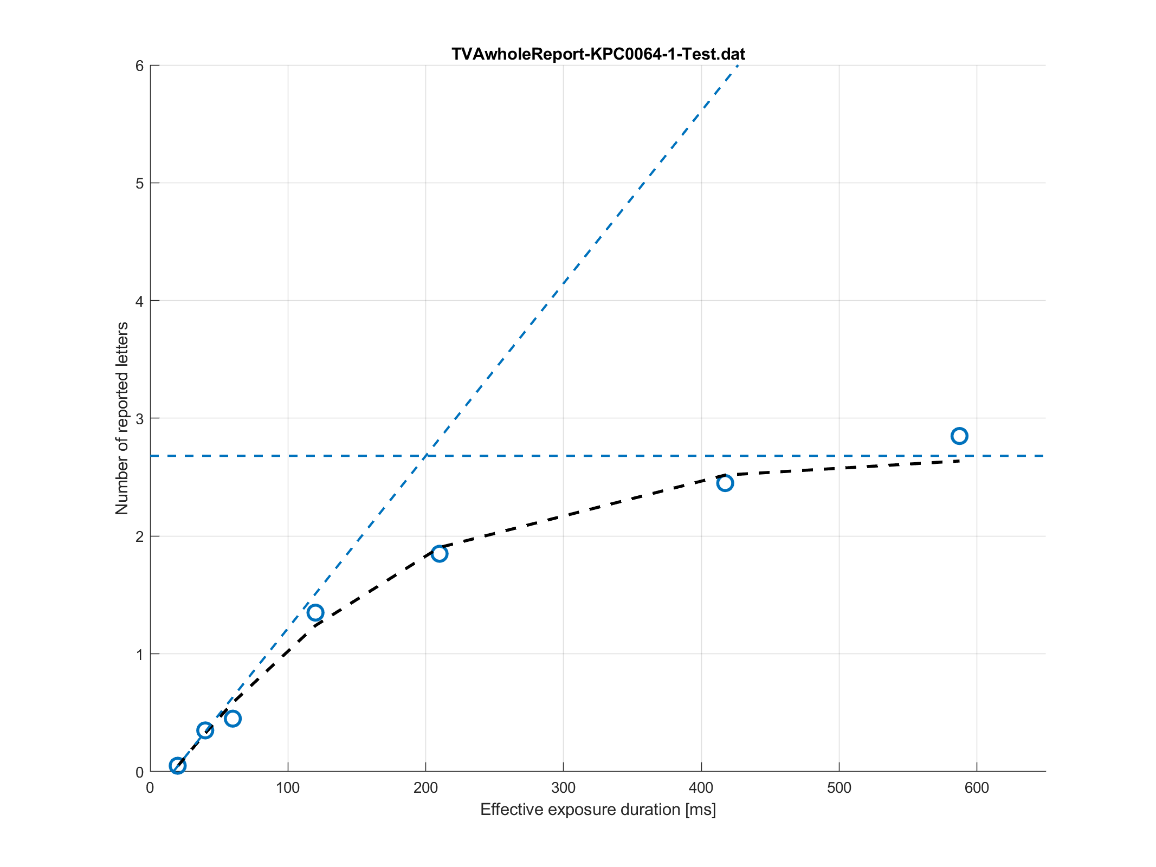

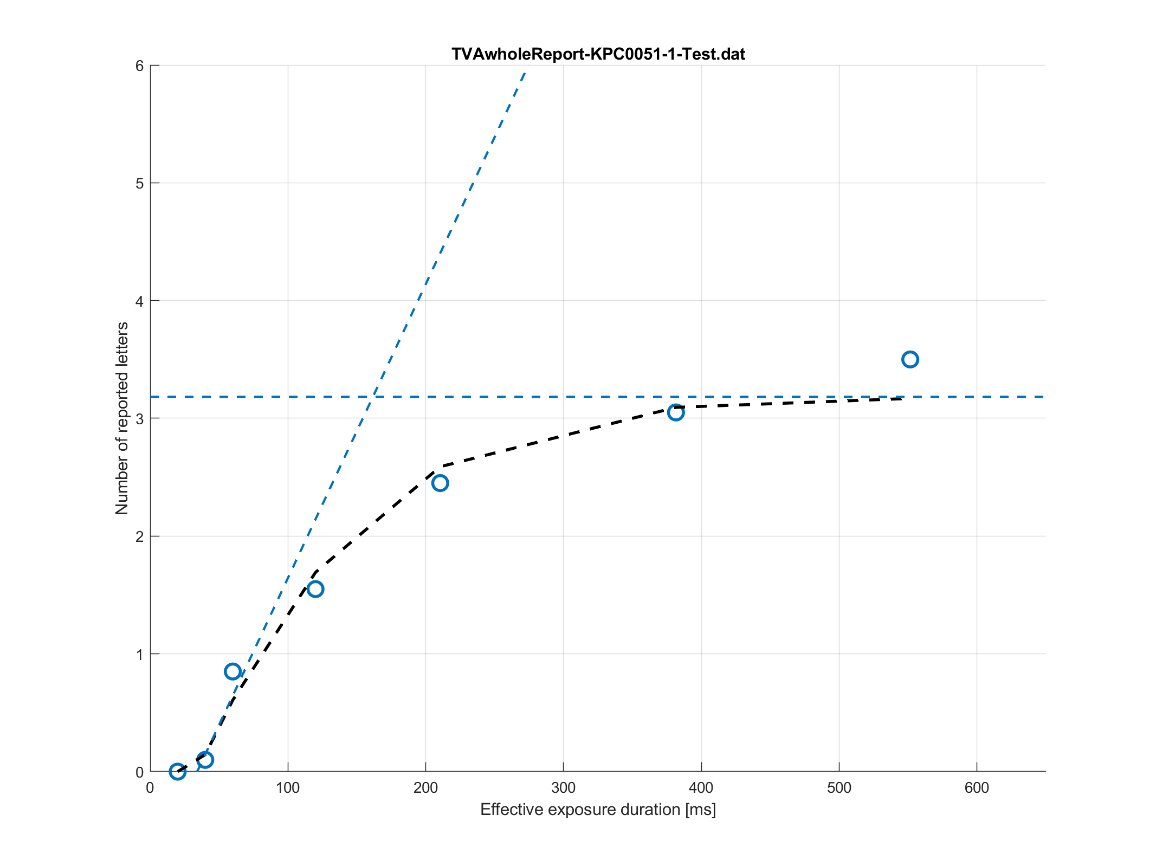

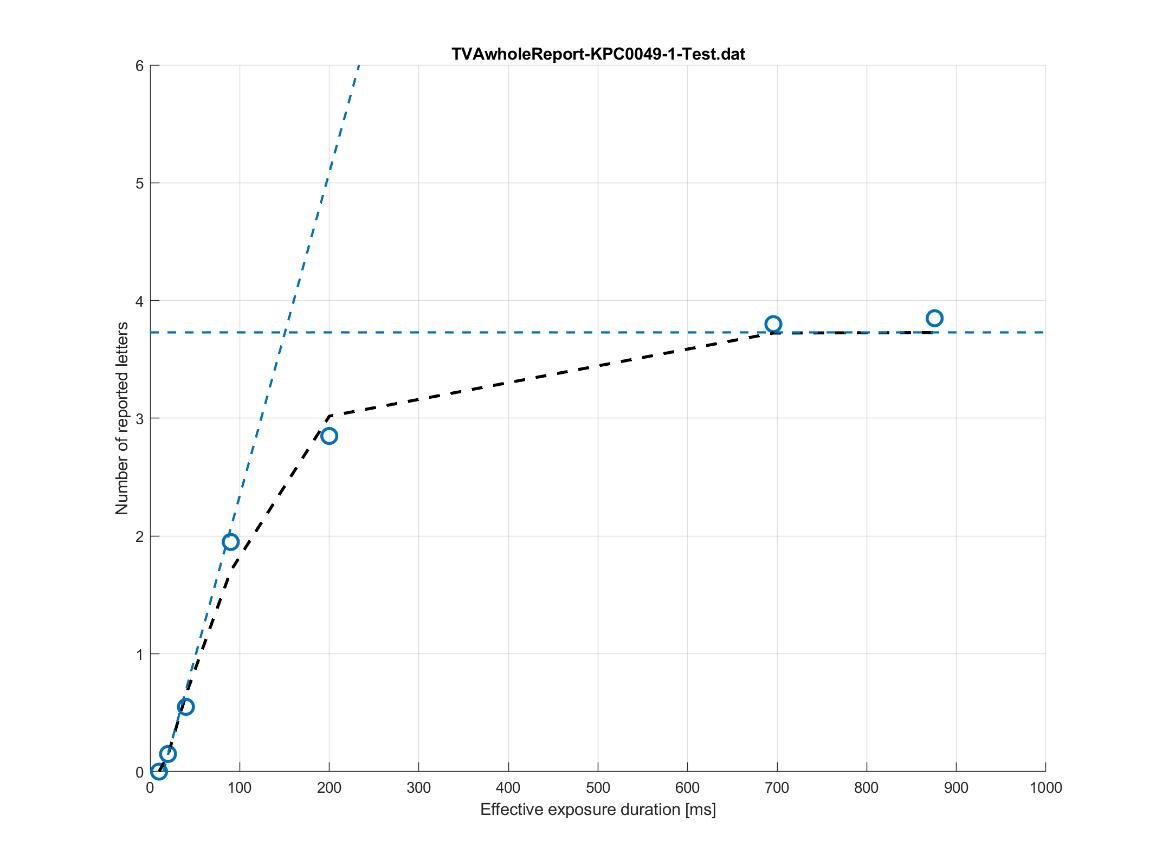

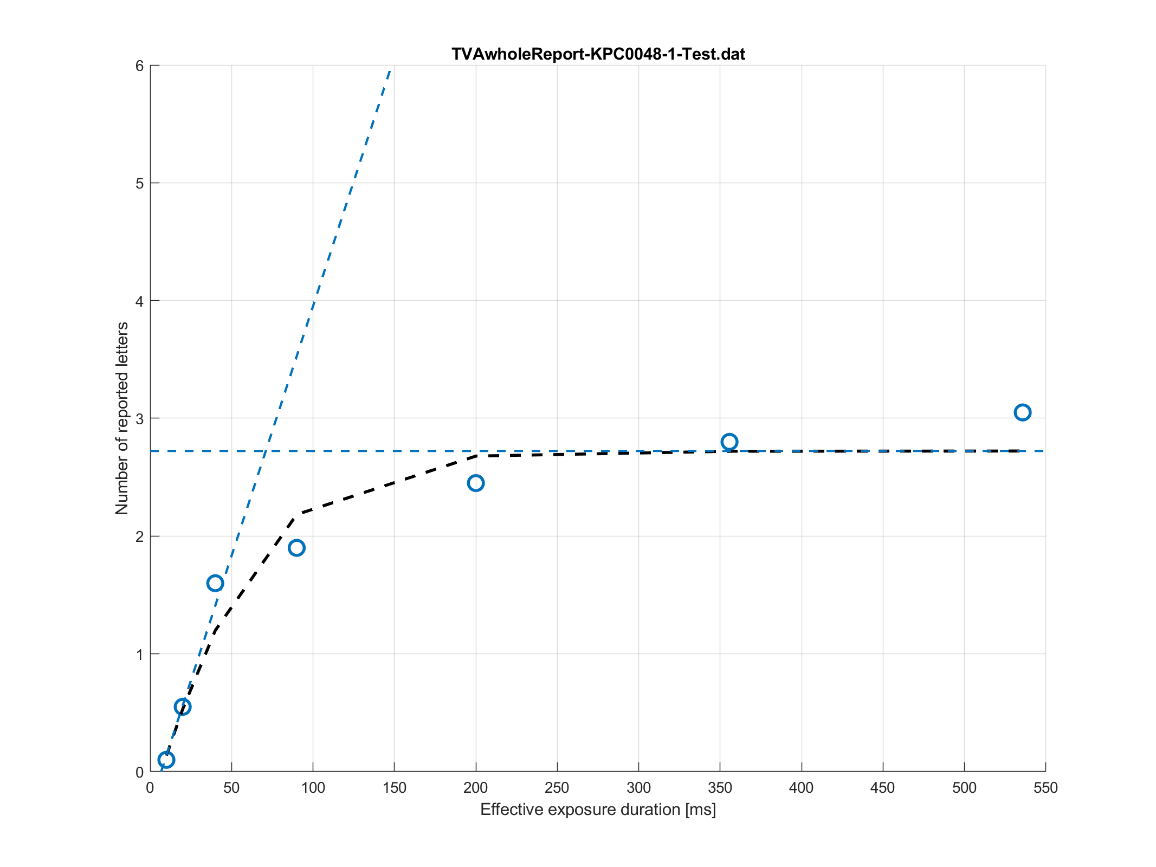

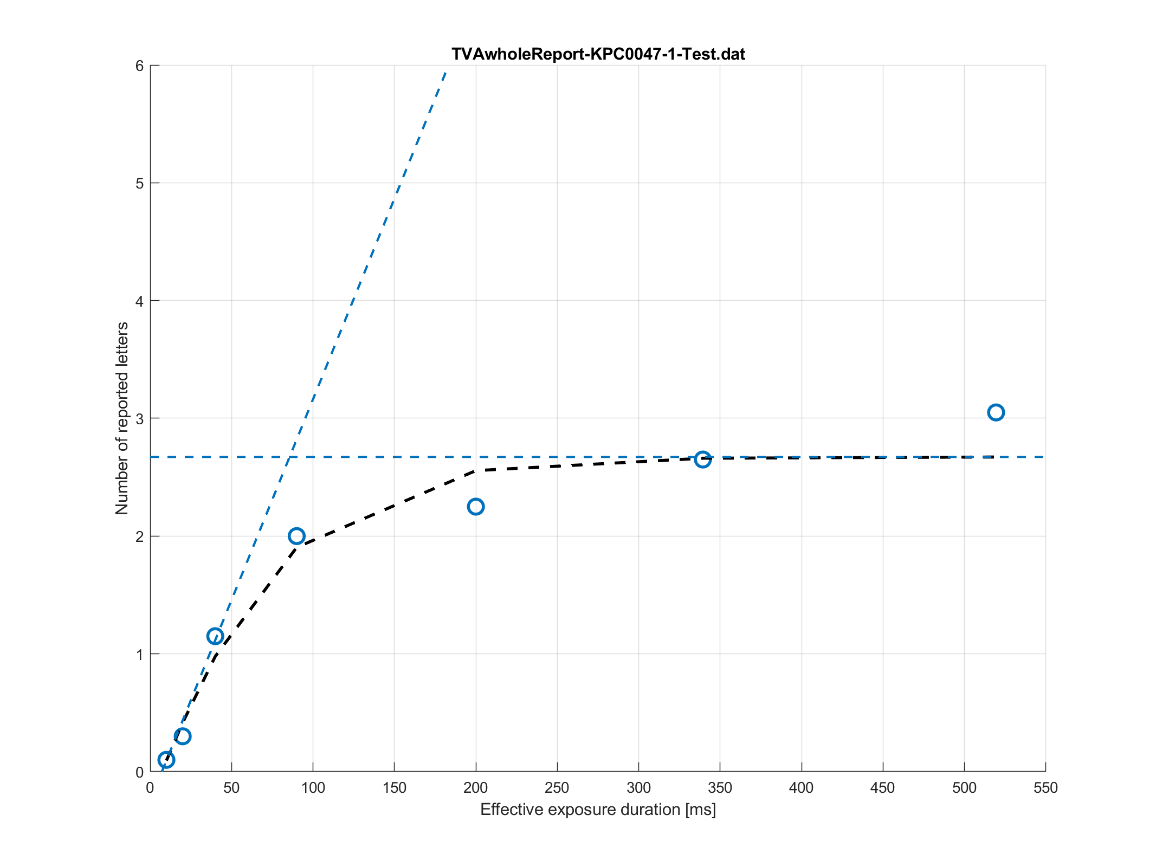

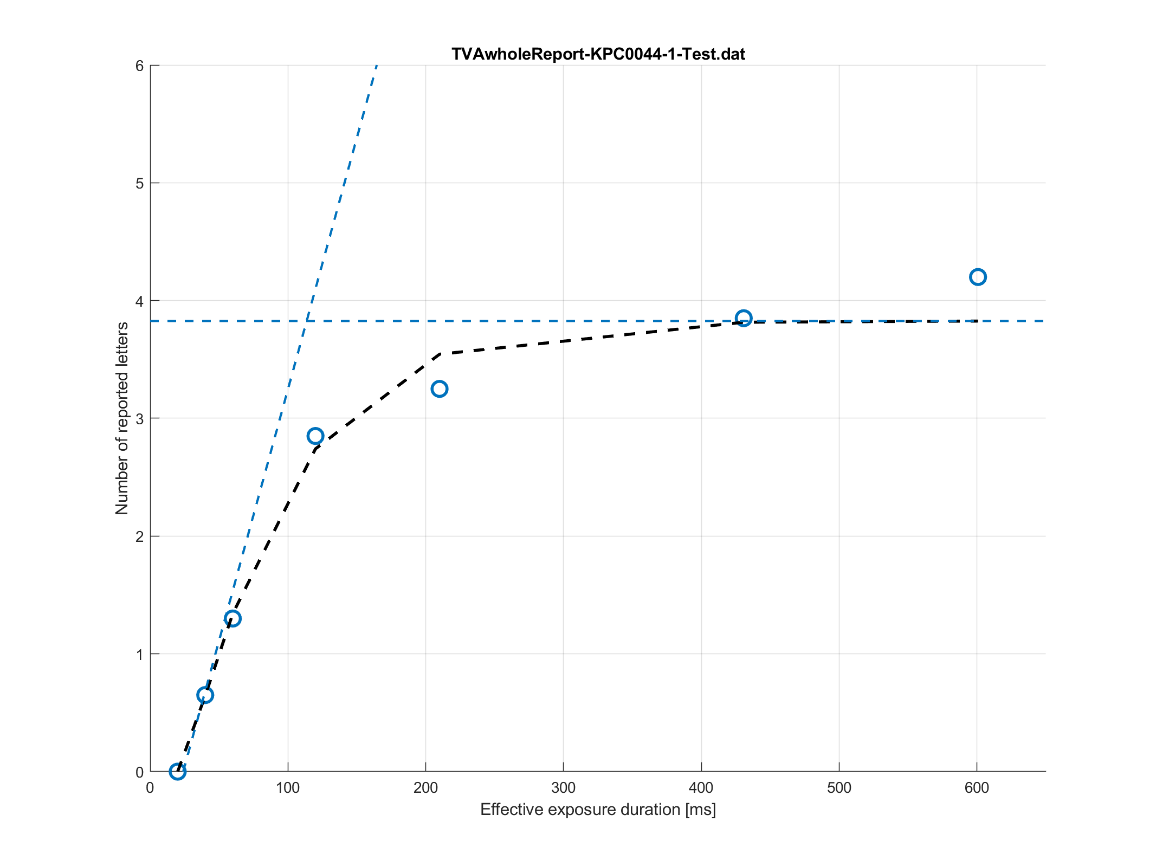

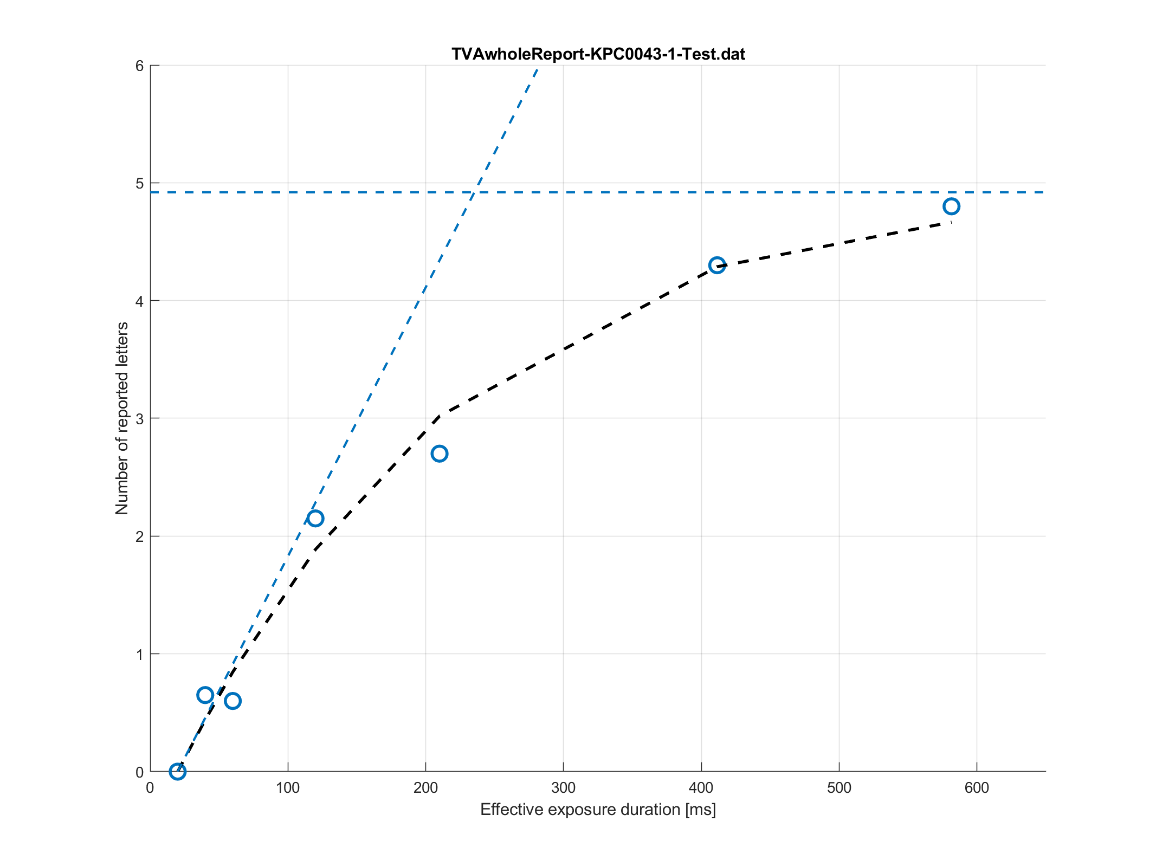

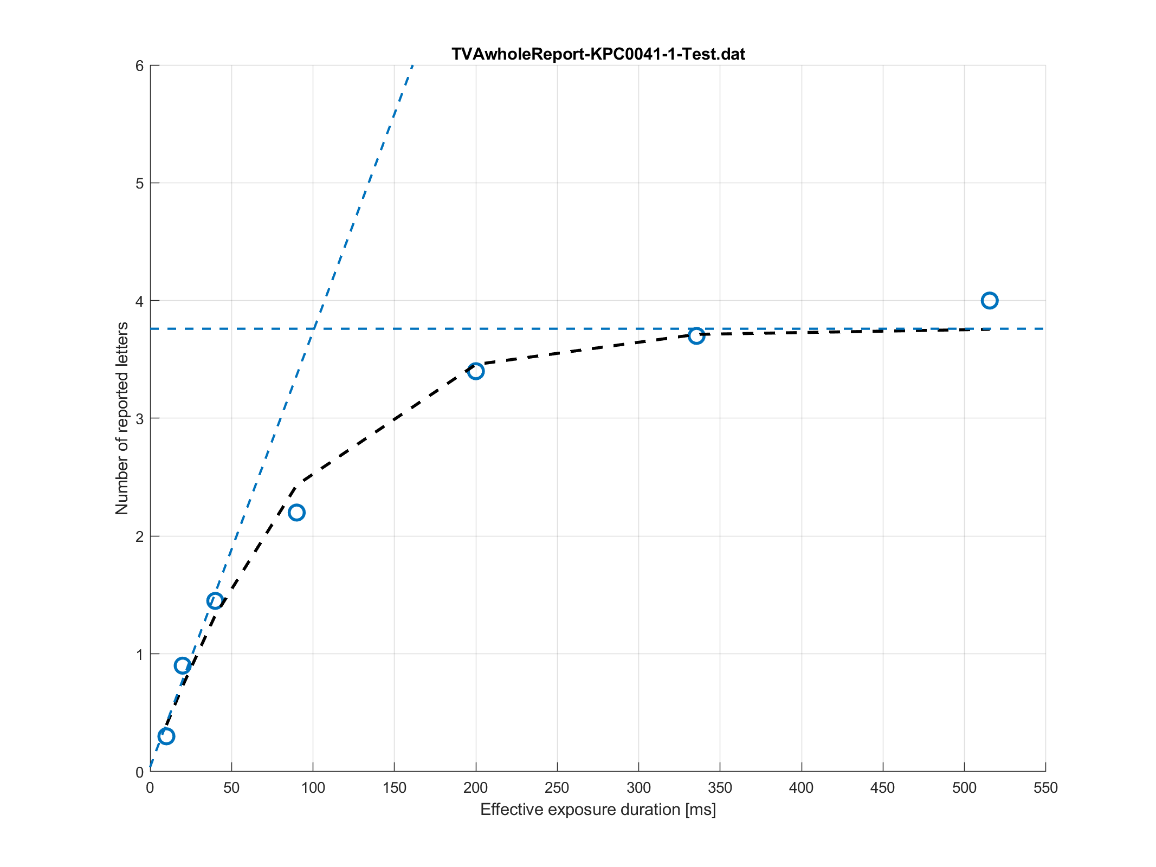

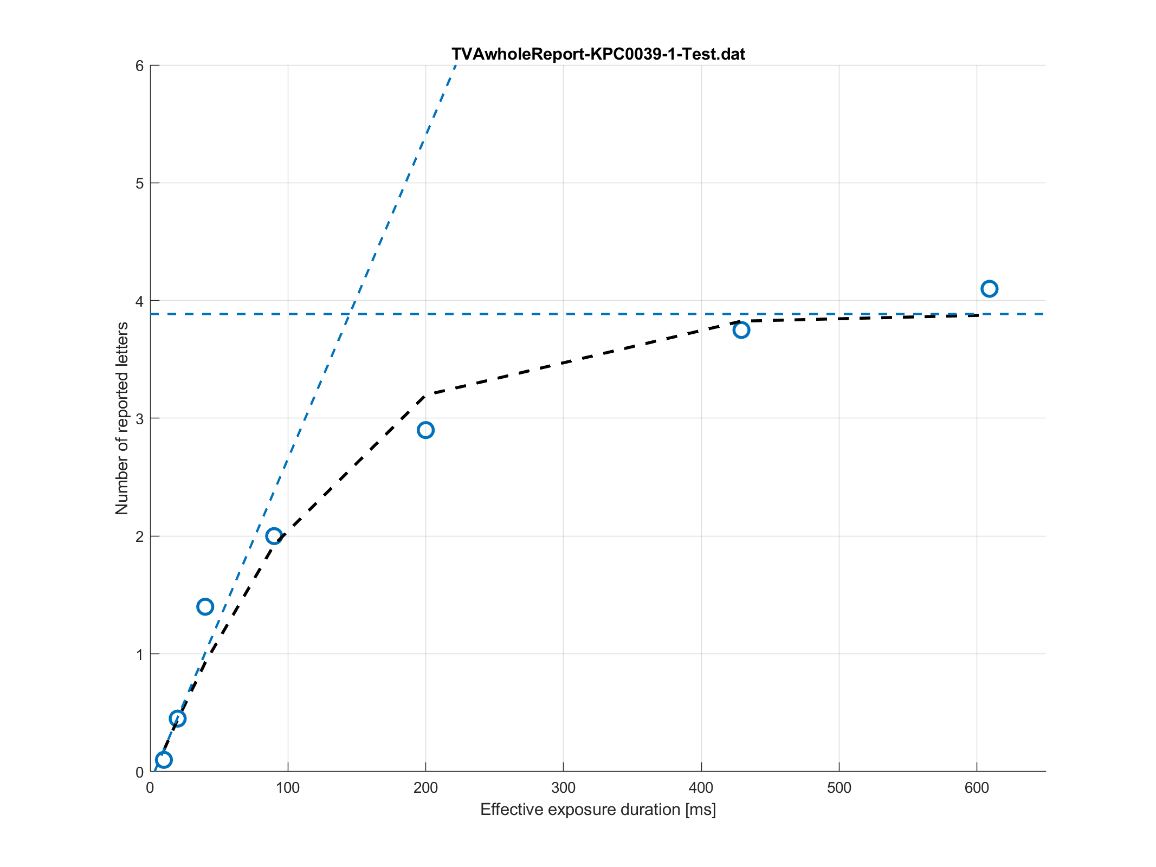

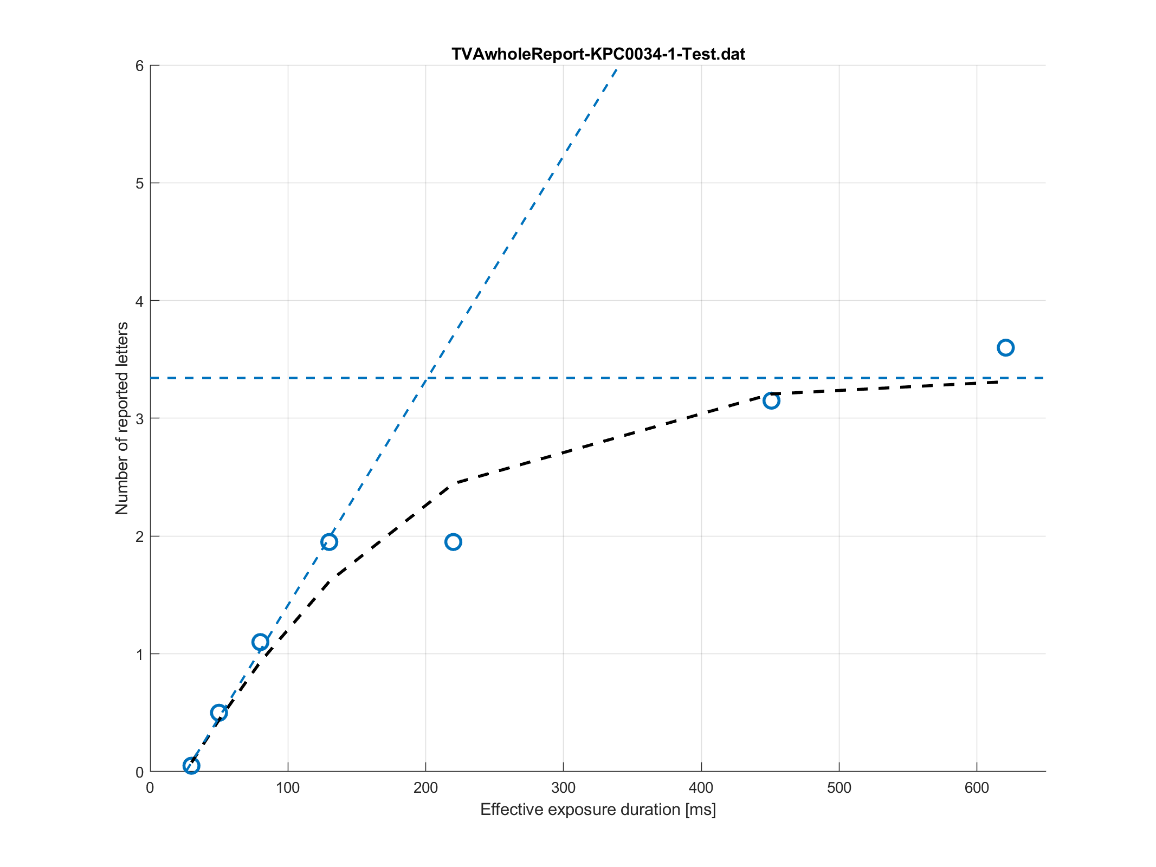

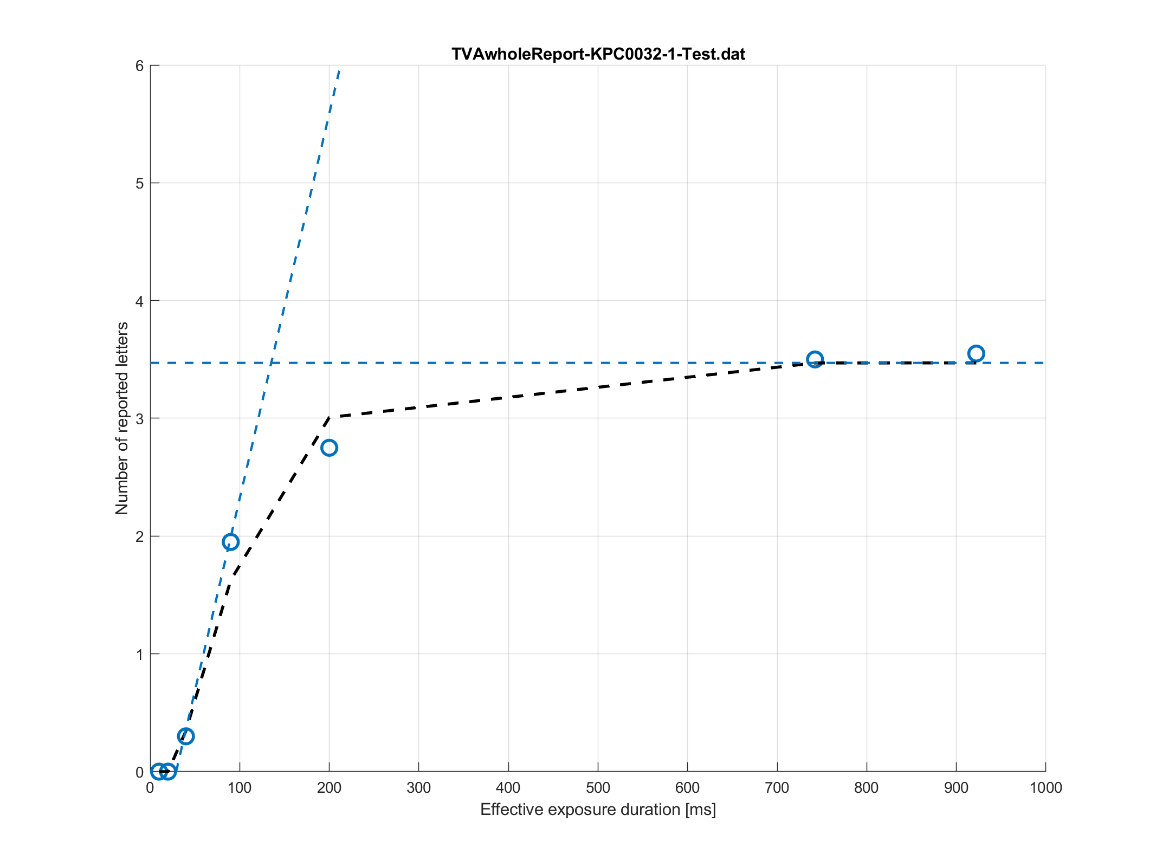

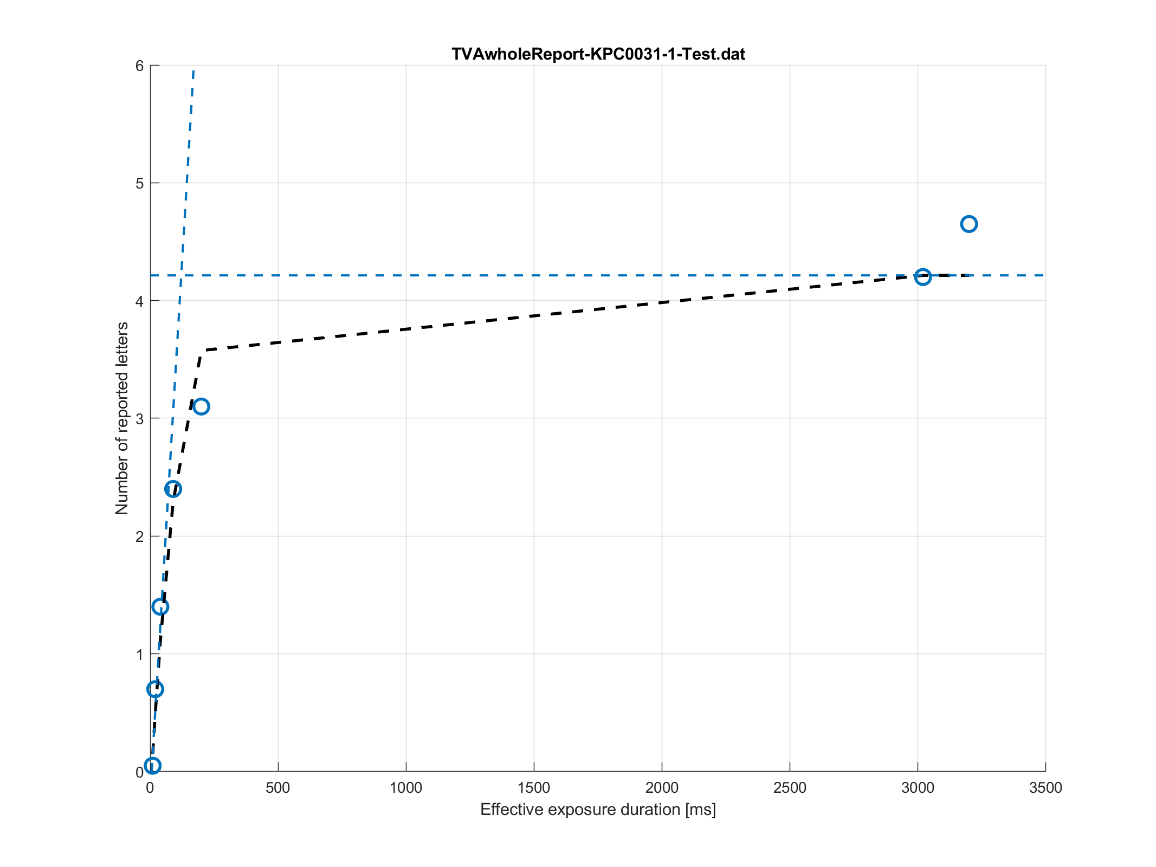

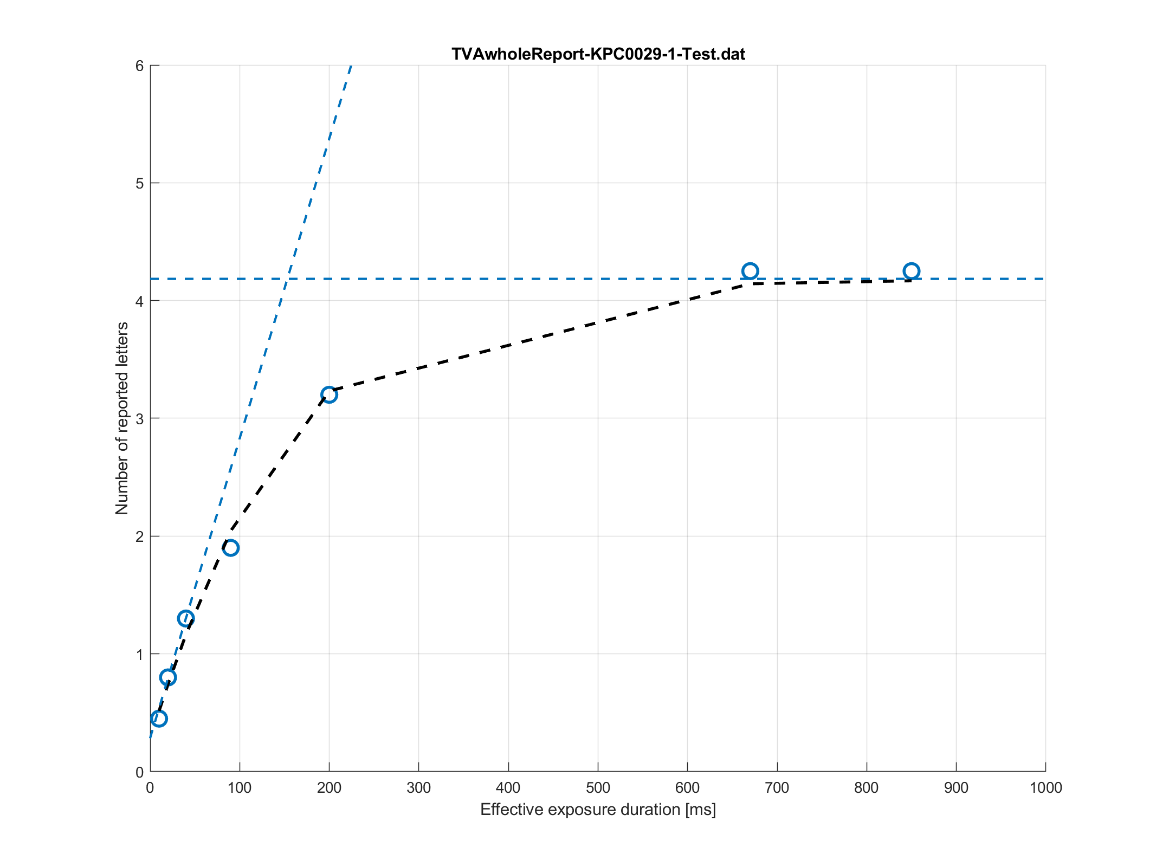

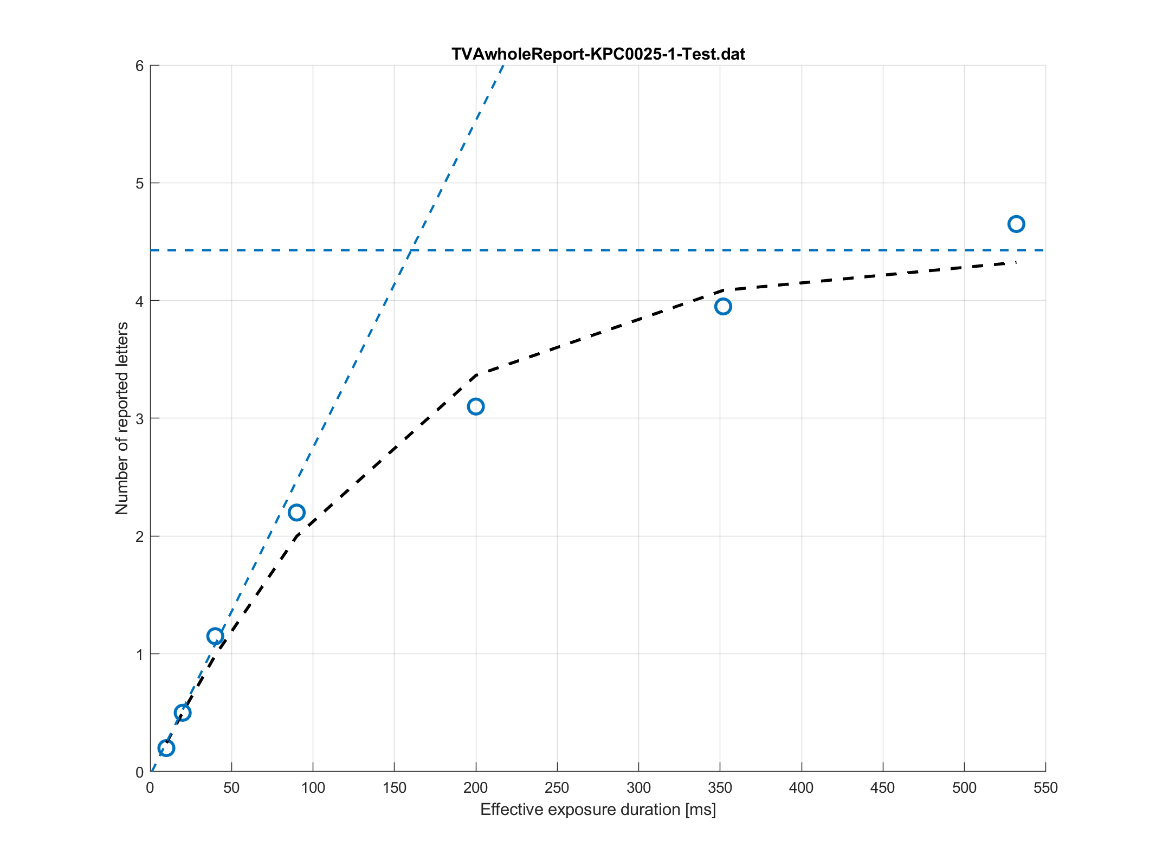

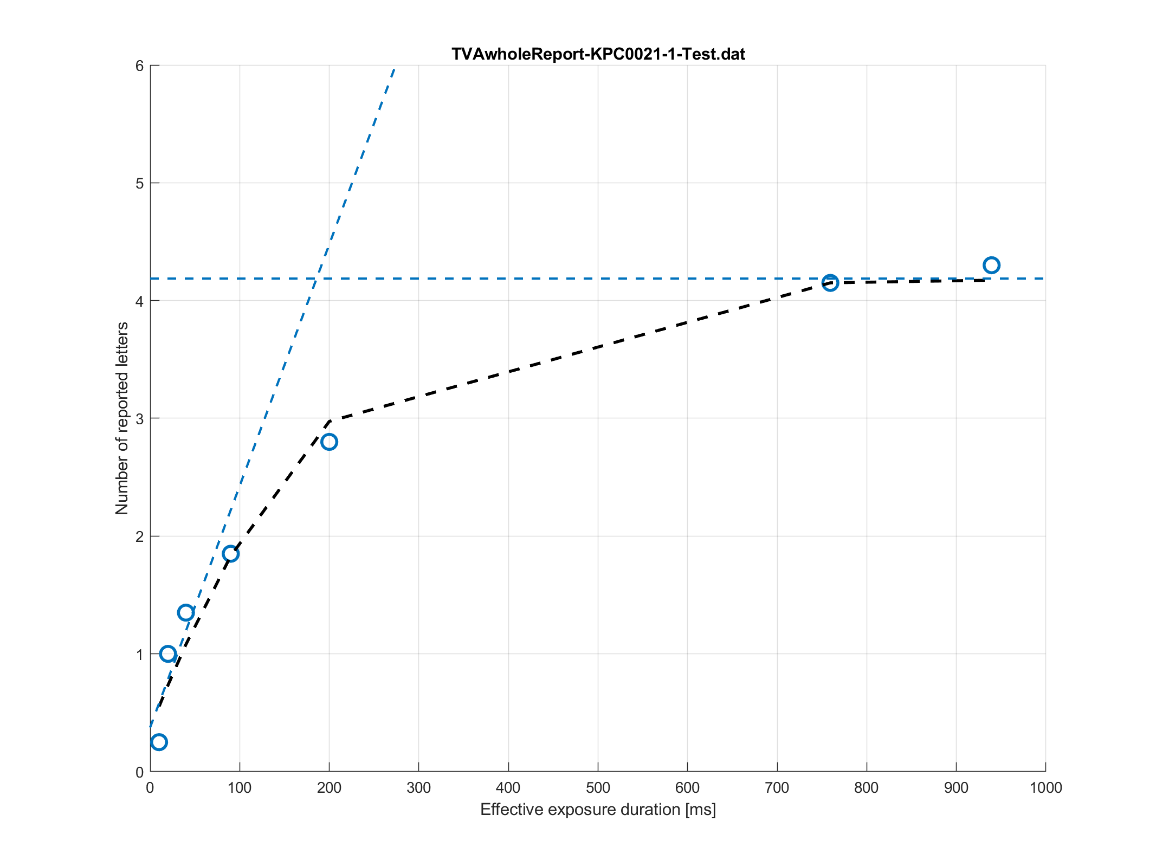

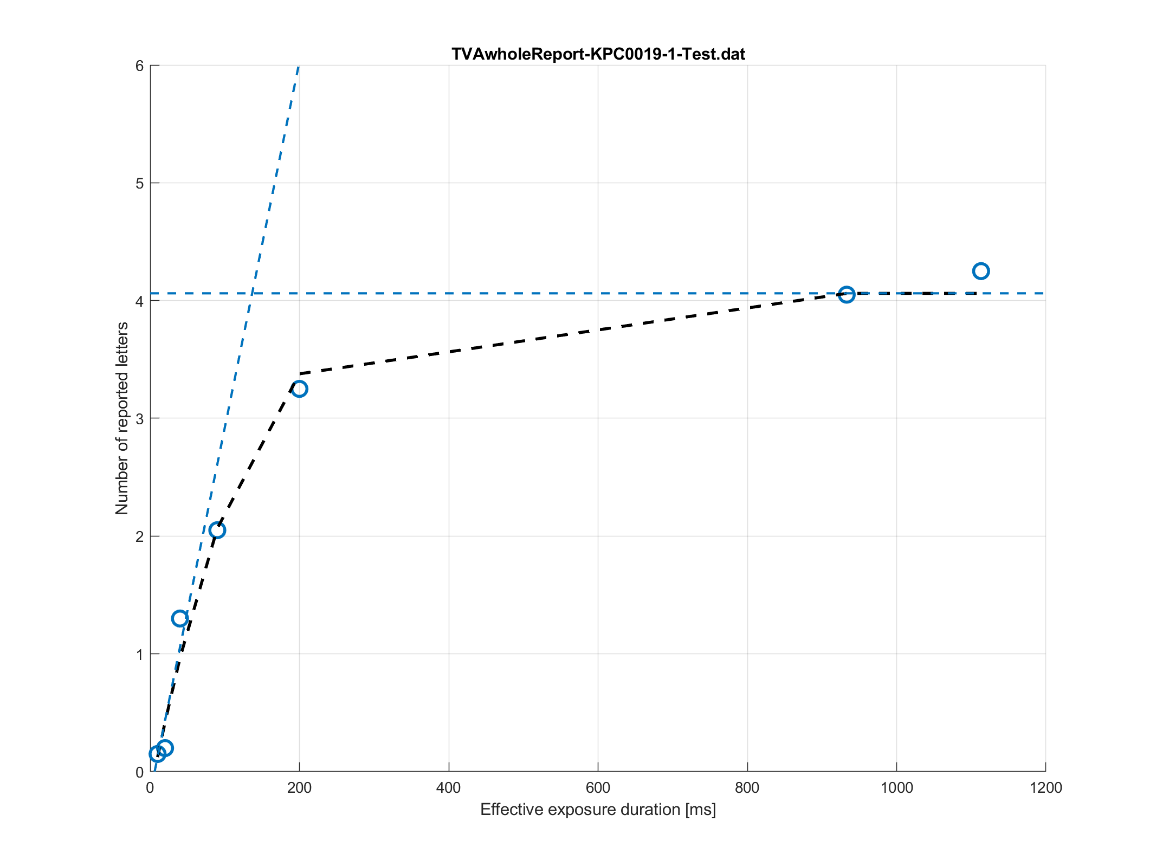

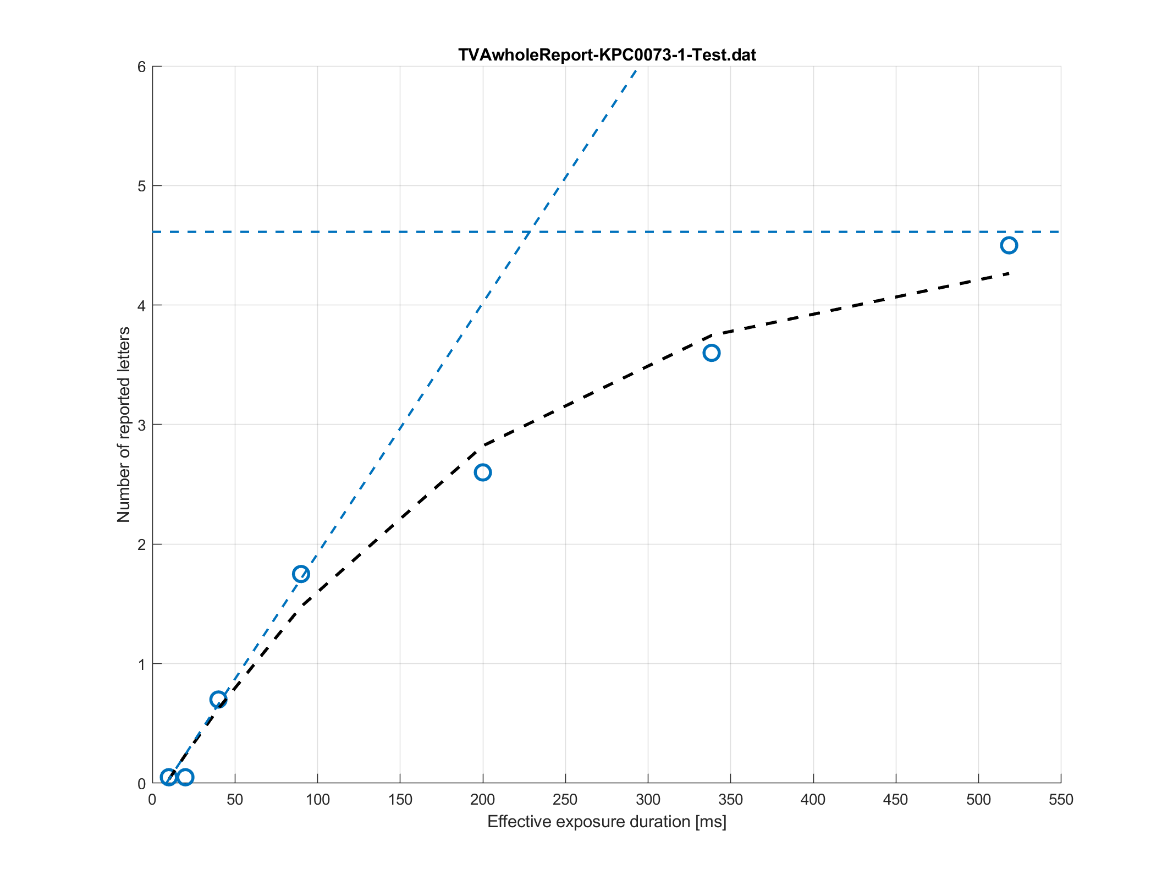

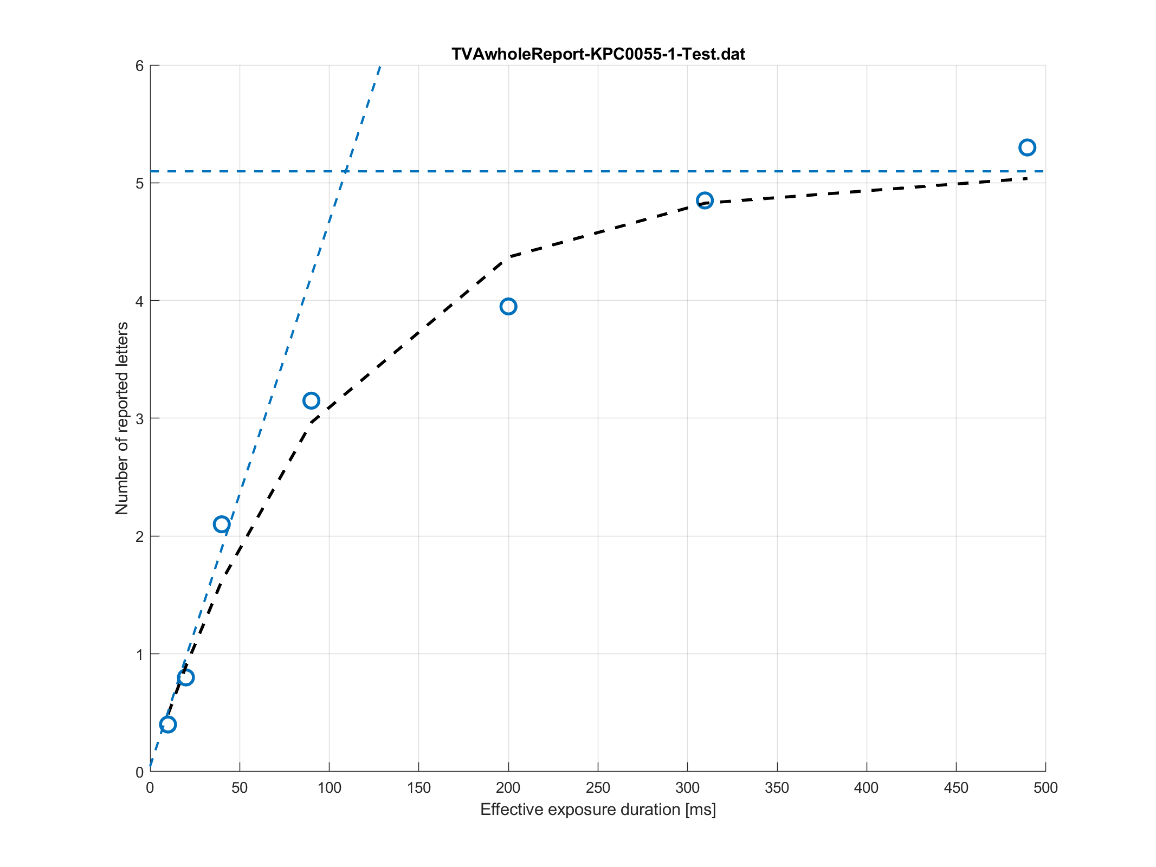

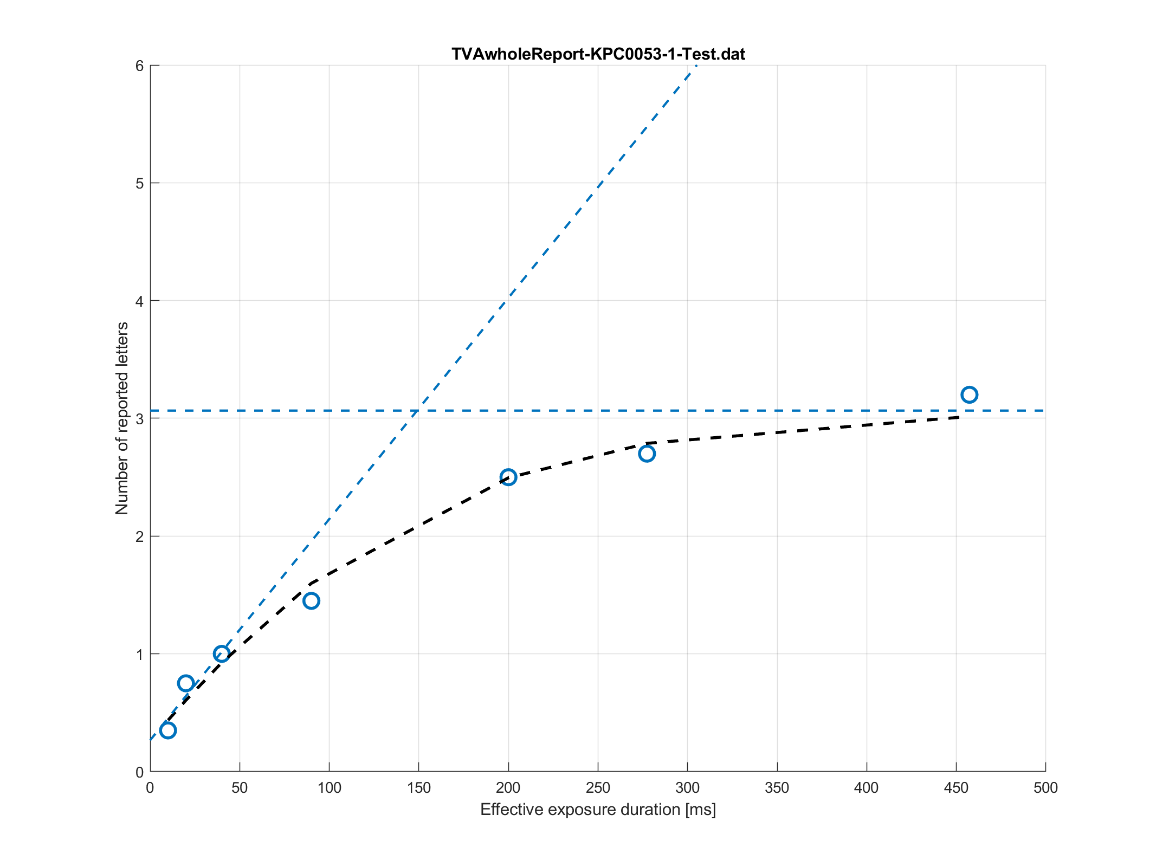

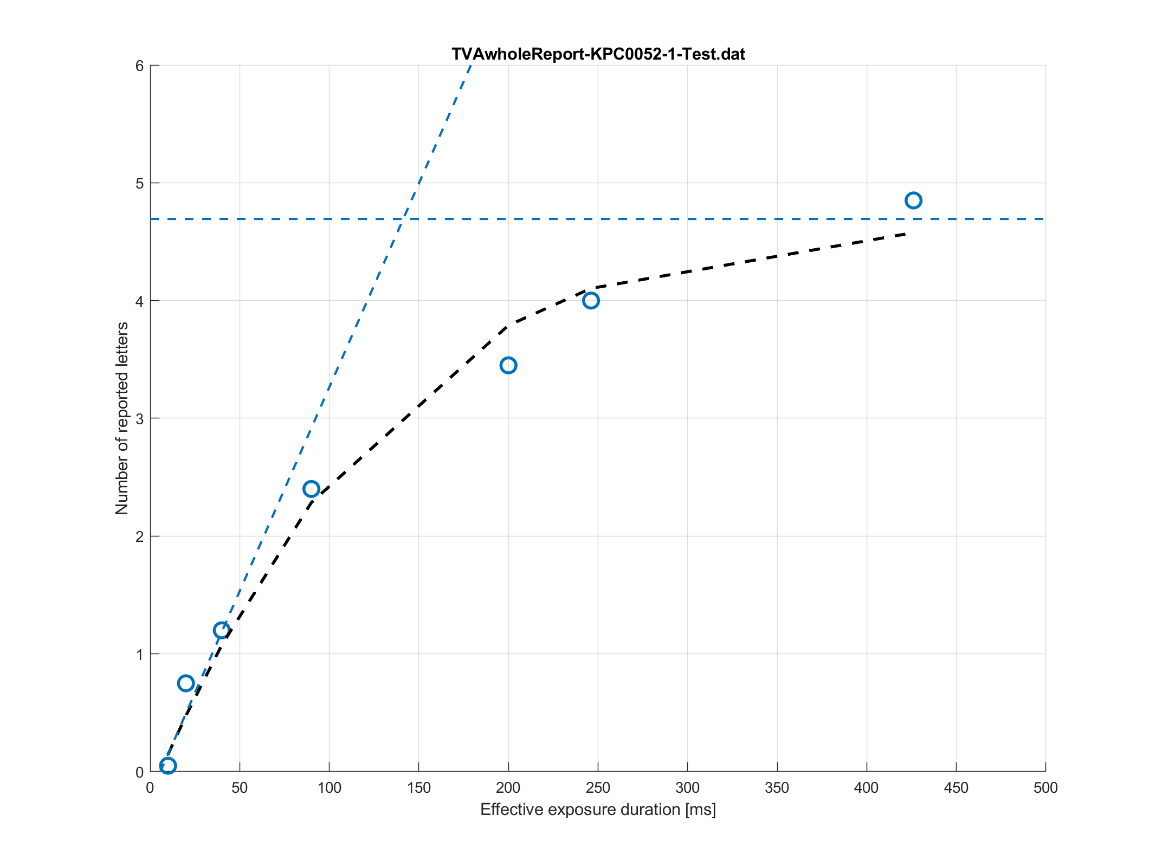

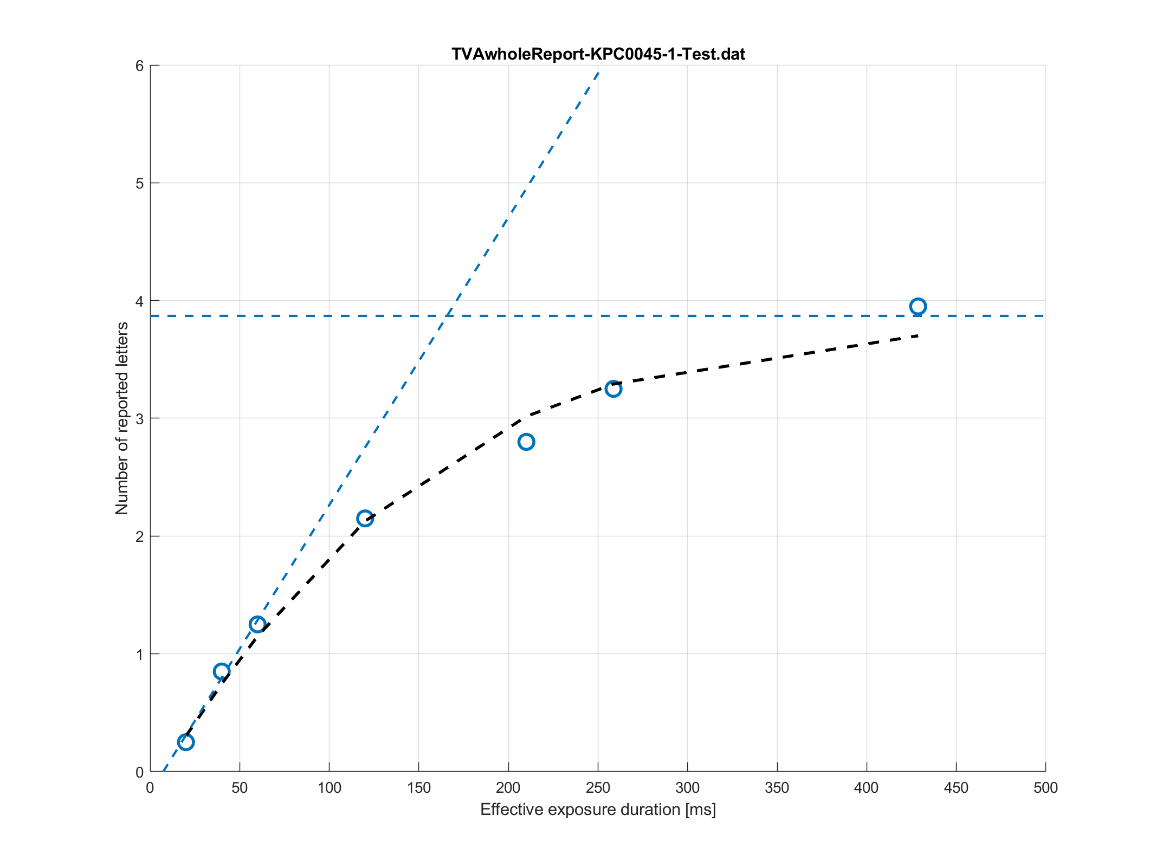

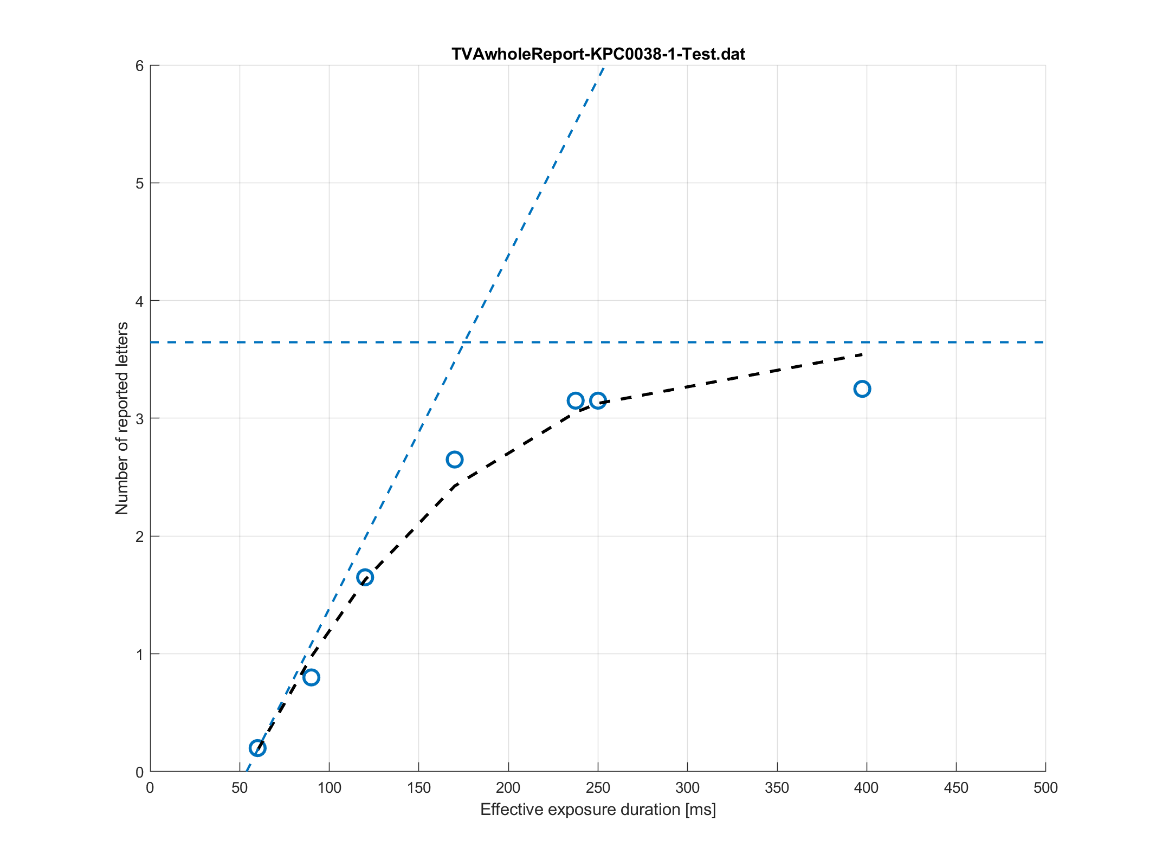

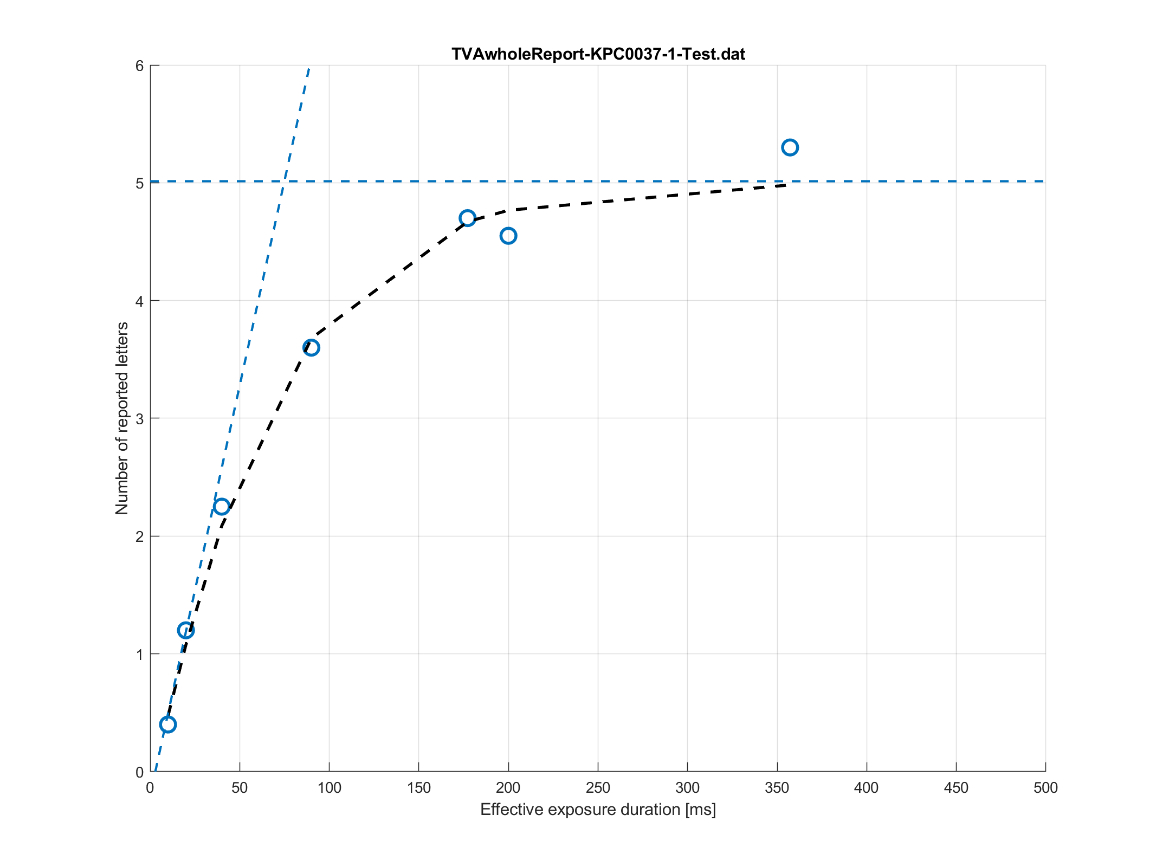

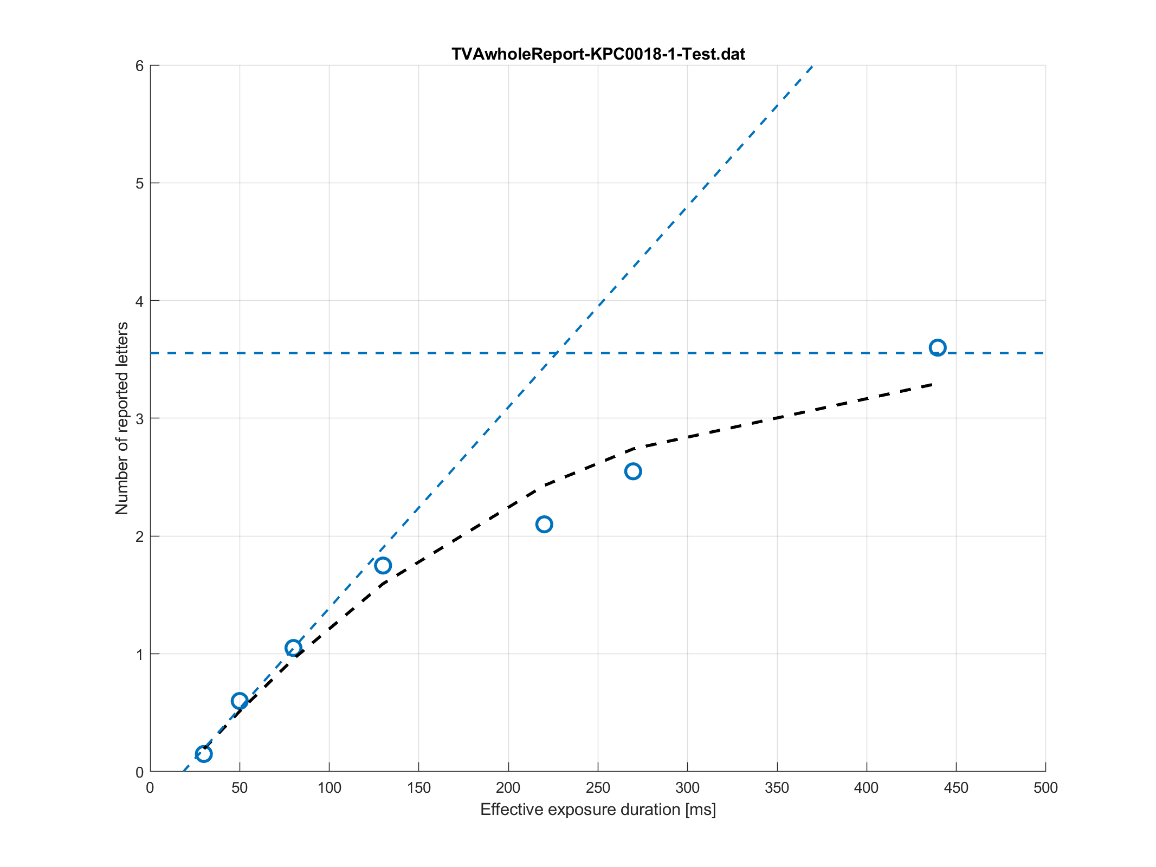

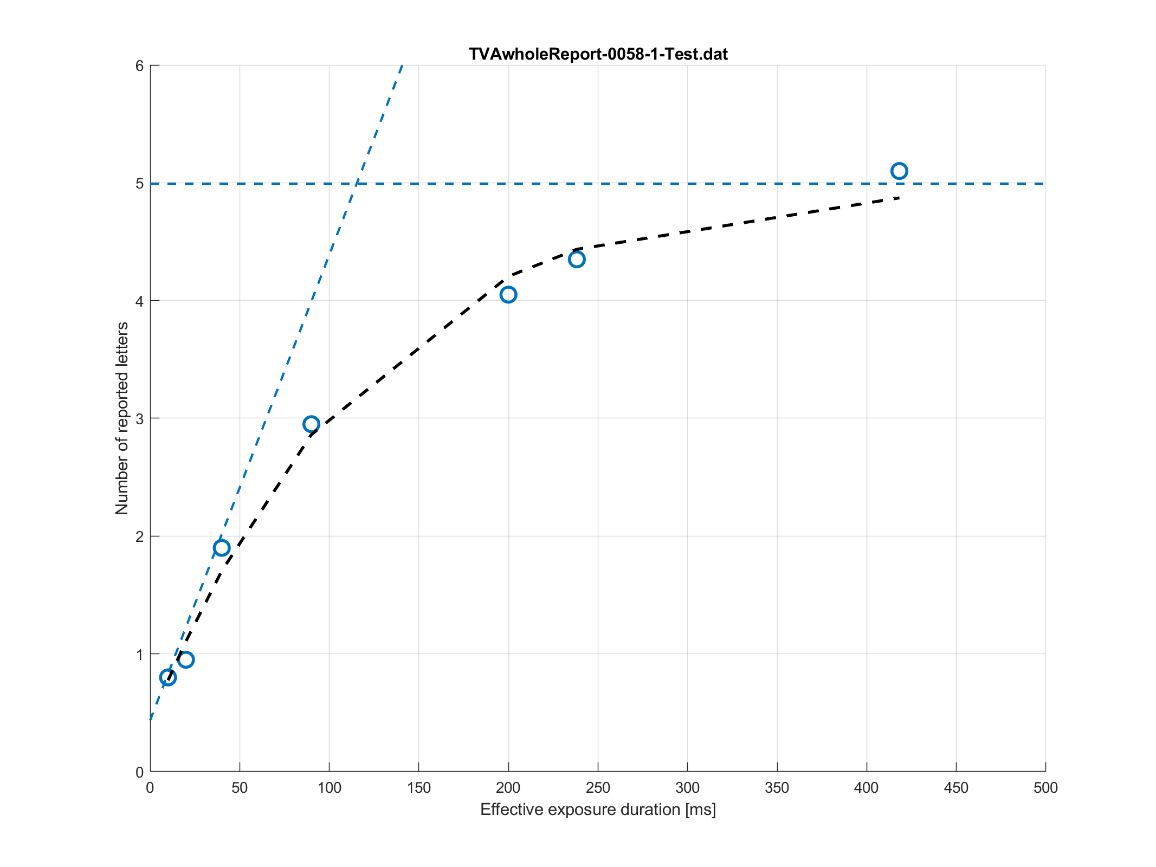

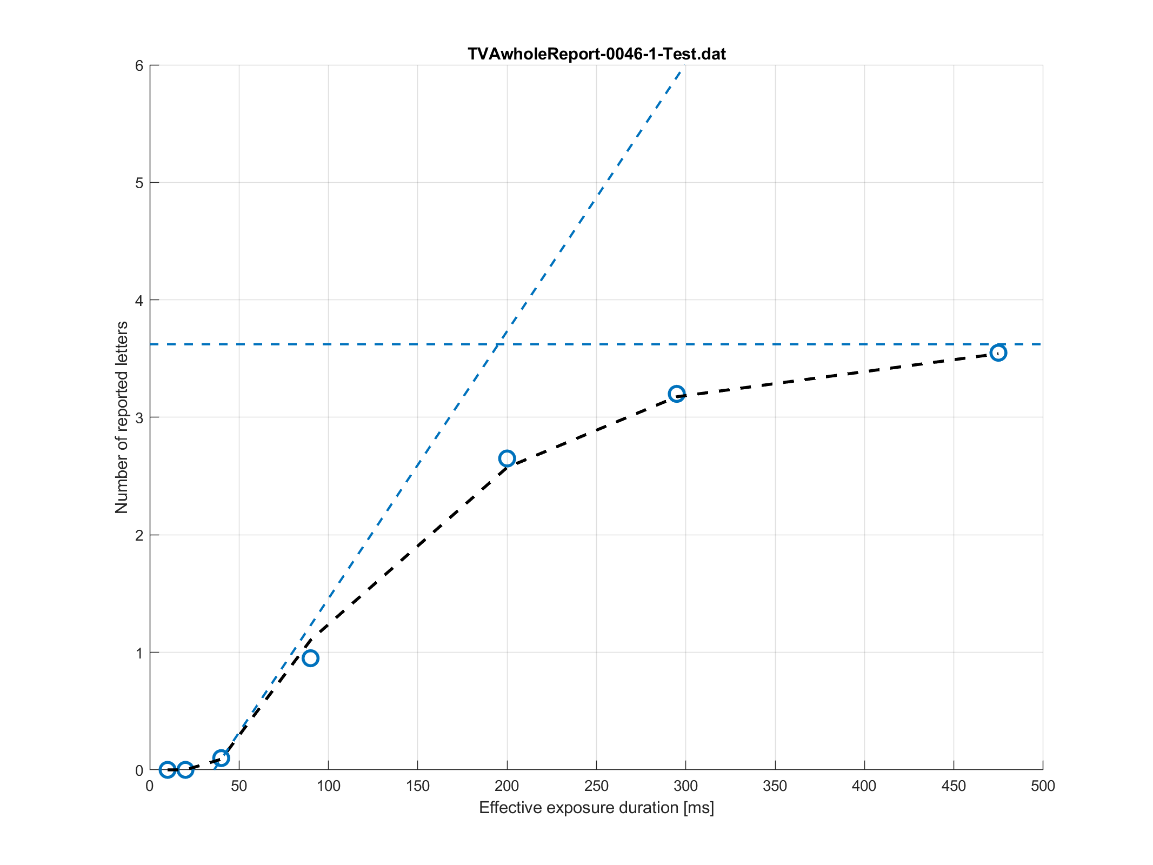

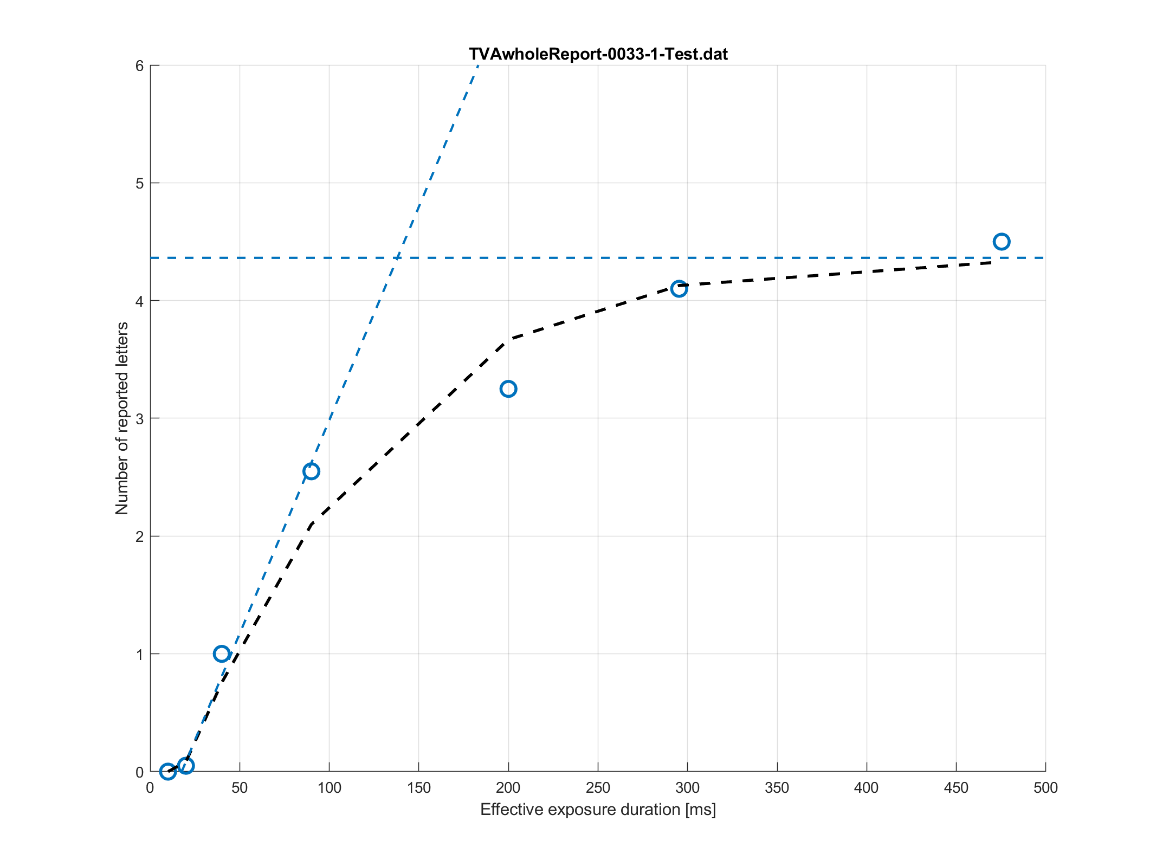

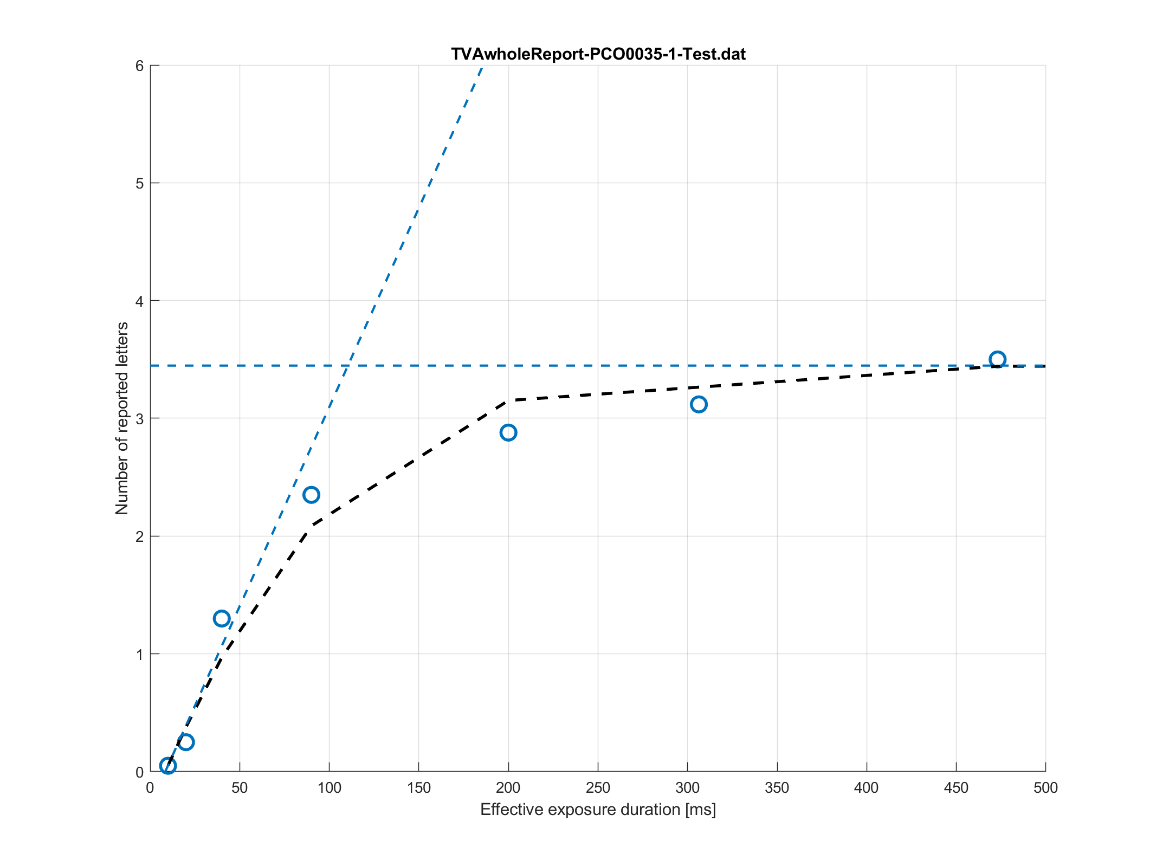

Supplement: Supplementary file 1 — Supplementary file1 (DOCX 4471 KB) [file 415_2023_11819_MOESM1_ESM.docx]
